# Resolution of deep angiosperm phylogeny using conserved nuclear genes and estimates of early divergence times

**DOI:** 10.1038/ncomms5956

**Published:** 2014-09-24

**Authors:** Liping Zeng, Qiang Zhang, Renran Sun, Hongzhi Kong, Ning Zhang, Hong Ma

**Affiliations:** 1 State Key Laboratory of Genetic Engineering and Collaborative Innovation Center for Genetics and Development, Ministry of Education Key Laboratory of Biodiversity Sciences and Ecological Engineering, Institute of Plant Biology, Institute of Biodiversity Science, Center for Evolutionary Biology, School of Life Sciences, Fudan University, 220 Handan Road, Yangpu District, Shanghai 200433, China; 2 Guangxi Institute of Botany, Guangxi Zhuang Autonomous Region and the Chinese Academy of Sciences, Guilin 541006, China; 3 State Key Laboratory of Systematic and Evolutionary Botany, Institute of Botany, Chinese Academy of Sciences, Beijing 100093, China; 4 Department of Botany, National Museum of Natural History, MRC 166, Smithsonian Institution, Washington DC 20560, USA; 5 Institutes of Biomedical Sciences, Fudan University, Shanghai 200032, China

## Abstract

Angiosperms are the most successful plants and support human livelihood and ecosystems. Angiosperm phylogeny is the foundation of studies of gene function and phenotypic evolution, divergence time estimation and biogeography. The relationship of the five divergent groups of the Mesangiospermae (~99.95% of extant angiosperms) remains uncertain, with multiple hypotheses reported in the literature. Here transcriptome data sets are obtained from 26 species lacking sequenced genomes, representing each of the five groups: eudicots, monocots, magnoliids, Chloranthaceae and Ceratophyllaceae. Phylogenetic analyses using 59 carefully selected low-copy nuclear genes resulted in highly supported relationships: sisterhood of eudicots and a clade containing Chloranthaceae and Ceratophyllaceae, with magnoliids being the next sister group, followed by monocots. Our topology allows a re-examination of the evolutionary patterns of 110 morphological characters. The molecular clock estimates of Mesangiospermae diversification during the late to middle Jurassic correspond well to the origins of some insects, which may have been a factor facilitating early angiosperm radiation.

## Abstract (web summary)

The phylogenetic relationships of Angiosperms remain uncertain. Here, the authors reconstruct well-supported phylogenetic relationships of the five major groups of Mesangiospermae and estimate divergence times and evolutionary patterns of plant morphological characters.

## Graphical abstract

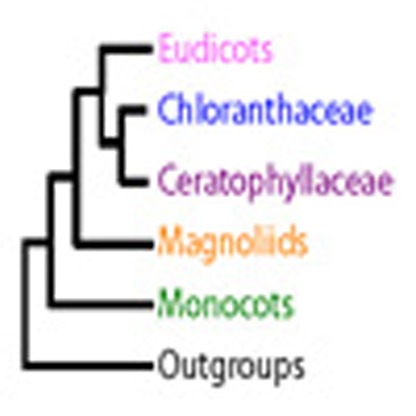

Angiosperms, that is, flowering plants, are one of the most diverse and species-rich groups on Earth and are the major components of the current terrestrial ecosystems^1^. The geologically sudden appearance of diverse angiosperm fossils could not be explained by Darwin’s evolutionary theory of gradual changes and prompted his reference of ‘abominable mystery’^2^. Such angiosperm diversity has since been extended by recent fossil discoveries of the now extinct early angiosperm *Archaefructus*, waterlilies (Nymphaeales) and a relative of buttercup (Ranunculales, sister to all other eudicots) in the Early Cretaceous (~125 million years ago) or even earlier^3^,^4^,^5^. Decades of efforts have produced an angiosperm phylogeny that defines major groups and identifies small sister lineages to the vast majority of angiosperm diversity^6^. Among the estimated 350,000 angiosperm species ( http://www.theplantlist.org/), only ~175 species form three small successive sister groups to other groups, Amborellales (a single species of understory bush found in New Caledonia, the South Pacific), Nymphaeales (waterlilies and related plants) and Austrobaileyales (star anise and relatives), collectively named the ANITA grade^7^. The remaining 99.95% of extant angiosperms form Mesangiospermae, a highly supported monophyletic group composed of five major lineages: eudicots, monocots, magnoliids, Chloranthaceae and Ceratophyllaceae^8^. Therefore, after a few early divergent branches in the ANITA grade, the highly diverse and species-rich Mesangiospermae represent the rapid expansion of early angiosperms and account for nearly all extant angiosperm diversities.

Within Mesangiospermae, eudicots and monocots are the two largest and diversified groups, containing ~75% and 20% of angiosperm species, respectively. Eudicots include many familiar fruits (for example, apple, orange and melons), beans, nuts (walnut and chestnut), vegetables (for example, tomato, lettuce and cabbage), spices and flowers (roses and carnations), whereas monocots include major grains (maize, rice and wheat) and flowers (orchids, tulip and lilies), as well as palm trees. Magnoliids, the third major group with ~9,000 species, contains some of the most ‘early angiosperms’ defined in earlier studies, such as magnolia, as well as black pepper and avocado^9^. The other two groups, Chloranthaceae and Ceratophyllaceae, are small and morphological unusual with only 77 and 6 species, respectively; however, they represent separate ancient lineages with evolutionary significance. Chloranthaceae has the simplest flowers and was once considered as the most ‘primitive’ group because of its extensive and early fossil records^9^,^10^. Ceratophyllaceae is a group of cosmopolitan aquatic plants with unusual morphologies, including inconspicuous flowers and greatly reduced roots, with an ancient origin supported by related fossils since the early Cretaceous^11^.

Resolving the relationships among these five groups will inform the order of their divergence and identify the sister groups of eudicots and monocots, the two largest angiosperm groups. The divergence order is crucial for estimating the time of the rapid angiosperm radiation and identifying possibly relevant contributing factors; moreover, knowledge of the sisters of eudicots and monocots is vital for understanding the origin and evolutionary patterns of characters. In the widely accepted Angiosperm Phylogeny Group III (APG III) system^6^, Ceratophyllaceae is sister to eudicots and they together are sister to monocots; then, Chloranthaceae and magnoliids form a clade that is sister to the (eudicots–Ceratophyllaceae)–monocots clade (Fig. 1a)^6^. According to this hypothesis, monocots separated from the clade of eudicots and Ceratophyllaceae after the divergence of a series of small lineages (that is, the ANITA grade, magnoliids and Chloranthaceae)^12^. However, the relationships among the 5 mesangiosperm groups are far from resolved, with 15 proposed topologies having low-to-moderate support, including those hypothesizing sisterhood of monocots with either eudicots or magnoliids^7^,^12^,^13^,^14^,^15^,^16^,^17^,^18^ (Fig. 1 and Supplementary Fig. 1). Therefore, the relationship of the five mesangiosperm groups has long been regarded as one of the most difficult problems remaining in angiosperm phylogeny^19^. In addition, the analyses of the order and relative time of divergence of major angiosperm groups have mainly relied on organellar genes and the results are still uncertain^20^,^21^. However, knowledge on divergence time plays important roles in understanding the evolution of angiosperms *per se* and their relation to other groups, such as ferns^22^, insects^23^, even dinosaurs^24^.

Previous angiosperm phylogenetic markers were mainly chloroplast and mitochondrial genes, as well as nuclear genes for ribosomal RNAs, with only a few protein-coding nuclear genes having been used in plant molecular phylogeny, especially above the family level^15^,^25^,^26^. Organellar genes are generally inherited uniparentally; in addition, recombination and gene conversion that have occurred in the plastid genome might also introduce biases and errors to phylogenetic reconstruction^19^. In contrast, nuclear genes are numerous and biparentally inherited; therefore, through extensive searches and selection, the use of sufficient number of appropriate nuclear genes can provide alternative evidence for relationships among early divergent angiosperms^27^. With the development of high-throughput sequencing technologies, nuclear gene sequences can be acquired cost-effectively from non-model species, as recently applied in phylogenomic studies of metazoan and fungal evolution^28^,^29^,^30^. Therefore, in this study, to resolve the relationships among the five lineages of Mesangiospermae, 26 transcriptome data sets were newly generated for phylogenetically critical species. Using a moderate number (59) of carefully selected low-copy nuclear genes, a topology with high statistical support was obtained. With this hypothesis, the divergence time of angiosperms and the evolutionary patterns of 110 morphological characters were assessed. Moreover, single-copy genes and genes from inverted repeated region (IR) of 86 plastid genomes were reanalysed extensively to identify possible causes of different topologies when using different datasets.

## Results

### Transcriptomes generated for new marker identification

Sequenced genomes of 30 angiosperm species are available (Supplementary Table 1), but they have uneven phylogenetic distribution, being concentrated in a few eudicot and monocot groups. Here, to provide a better representation of the five mesangiosperm lineages, 25 new angiosperm transcriptome data sets were generated (Table 1), including those of representatives for the three smaller groups (magnoliids, Chloranthaceae and Ceratophyllaceae), which lack sequenced genomes. In addition, representatives of small sister lineages of the majority of eudicots or monocots were especially selected because they are thought to be helpful for minimizing long-branch attraction (LBA)^31^. A transcriptome data set of the gymnosperm *Ginkgo biloba* was also generated as the outgroup. Combined with 30 angiosperms with sequenced genomes and 5 other angiosperms with large expressed sequence tag (EST) data, in total 61 species were sampled in this study, covering all or most orders of magnoliids (3/4), monocots (10/12), Chloranthaceae (1/1) and Ceratophyllaceae (1/1) (Supplementary Table 1).

### Orthologue identification and gene selection

Angiosperms have experienced several rounds of whole-genome duplications (WGDs)^32^,^33^ and subsequent gene losses, rendering some single-copy nuclear genes non-orthologous (that is, hidden paralogues) and thus possibly unsuitable for resolving the relationship among the five major groups. To identify orthologous genes and exclude potential ‘hidden paralogues’, >4,000 orthologous groups (OGs) were used as the starting gene sets for identification of phylogenetic markers. To reduce the possible effects of missing data on phylogenetic accuracy^34^, OGs were selected with putative orthologues found in ≥80% of the 26 species with newly generated transcriptome data sets (Table 1); in addition, only sequences of coding regions with the length ≥80% of the *Arabidopsis thaliana* homologue were retained for further analyses, ultimately resulting in 349 OGs (Supplementary Fig. 2). Next, 349 single-gene trees of 20 representative species with well-supported relationships (Fig. 2) were further used to determine the suitability of the genes as phylogenetic markers (Supplementary Fig. 3) and finally 54 nuclear genes were selected (Supplementary Table 2) (see details in Methods). In general, only one copy was found in the 30 sequenced angiosperm genomes, except for a few recent lineage-specific duplications (Supplementary Data 1). Orthologues of these 54 and 5 previously analysed genes (*SMC1*, *SMC2*, *MCM5*, *MSH1* and *MLH1*)^15^ were identified from 26 transcriptome data sets using HaMStR^35^ and verified by single-gene trees of the 61 species studied here. Genes with unusually long branches in single-gene trees, possibly due to sequencing errors, translation frameshift or other factors, were removed from the single-gene alignment manually. After concatenation, the aligned 59-gene supermatrix reached 25,589 amino acids and had gene coverages for species with transcriptomic and genomic data between 68.7% and 97.7% with an average of 90.9% (Supplementary Data 2).

### Mesangiosperms are divided into monocots and a dicot clade

Phylogenetic analyses produced identical topology with strong support using RAxML^36^ and MrBayes^37^ regardless of gene partition and evolutionary models (Fig. 3 and Supplementary Figs 4,5). In agreement with most previous studies, the lineages in the ANITA grade, that is, Amborellales, Nymphaeales and Austrobaileyales, were successive sisters to Mesangiospermae with strong support (Fig. 3)^7^,^16^. Furthermore, Mesangiospermae, each of its five major lineages and core eudicots were all recovered as monophyletic groups with 100% support. Most relationships within eudicots or monocots were congruent with previous studies, except for a few that were uncertain in earlier studies (such as the position of Vitaceae^16^,^38^ and the relationships among Liliales, Asparagales and the combined clade of Dioscoreales and Pandanales^39^,^40^).

**Figure 1:**
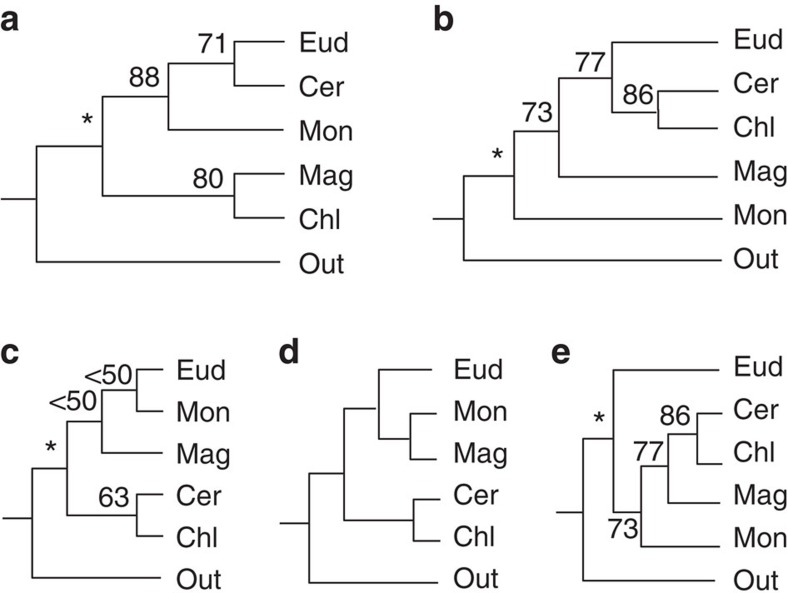
Five recently published representative topologies among eudicots (Eud), monocots (Mon), magnoliids (Mag), Ceratophyllaceae (Cer) and Chloranthaceae (Chl). Shown are topologies derived from: (**a**) refs 6, 12 and 16; (**b**) ref. 17; (**c**) ref. 40; (**d**) ref. 43; and (**e**) ref. 15. Out stands for the outgroup, an asterisk indicates a BS value of 100%.

Unlike previous studies, four of the five major mesangiosperm lineages, except monocots, form a strongly supported monophyletic clade, which we propose to be tentatively named ‘Mesodicots’ for its inclusion of 99.94% of extant dicot species (Fig. 3). Among Mesodicots, Chloranthaceae is sister to Ceratophyllaceae; then these two together are sister to eudicots, with magnoliids being the next group, with 99%, 98% and 94% bootstrap values, respectively, and 1.0 Bayesian posterior probability (PP) (Fig. 3). This topology is different from the widely recognized one in APG III, but was once previously recovered using the highly conserved plastid inverted repeat regions^17^ (Fig. 1b), albeit with low-to-moderate supports and not emphasized there. In addition, a recent study based on EST data sets from 101 taxa lacking both Chloranthaceae and Ceratophyllaceae also supported a topology with monocots being sister to a clade containing eudicots and magnoliids^41^. Furthermore, approximately unbiased test analyses of all 105 potential topologies for these 5 groups suggested that all 13 other previously reported topologies inferred by other molecular markers were rejected significantly (Table 2)^42^, although 6 alternative topologies could not be rejected significantly (Supplementary Data 3); one of these 6 was from an analysis of morphological characters^43^ and the others have not been well supported by previous analyses.

### Using <50 genes could resolve the deep angiosperm phylogeny

To investigate the minimal number of genes required to resolve the mesangiosperm relationships, we sampled various numbers of genes from 2 to 58 with increments of 2; for each number, 20 replicates of randomly selected genes were performed and a total of 580 matrices were generated. Next, the proportion of gene trees supporting the above hypothesis as shown in Fig. 3 was determined (see details in Methods). The proportion of gene trees that recovered the Chloranthaceae–Ceratophyllaceae (CC), eudicots–(CC) and magnoliids–(eudicots–(CC)) (that is, Mesodicots) relationships increased steadily with increasing gene numbers (Fig. 4a). The minimal numbers of genes required for 100% recovery of the CC, eudicots–(CC) and Mesodicots clades were 34, 40 and 48, respectively (Fig. 4a), suggesting that the most difficult angiosperm phylogenetic problems could be resolved by using fewer than 50 genes instead of hundreds of or more genes often used in many phylogenomic analyses.

**Figure 2:**
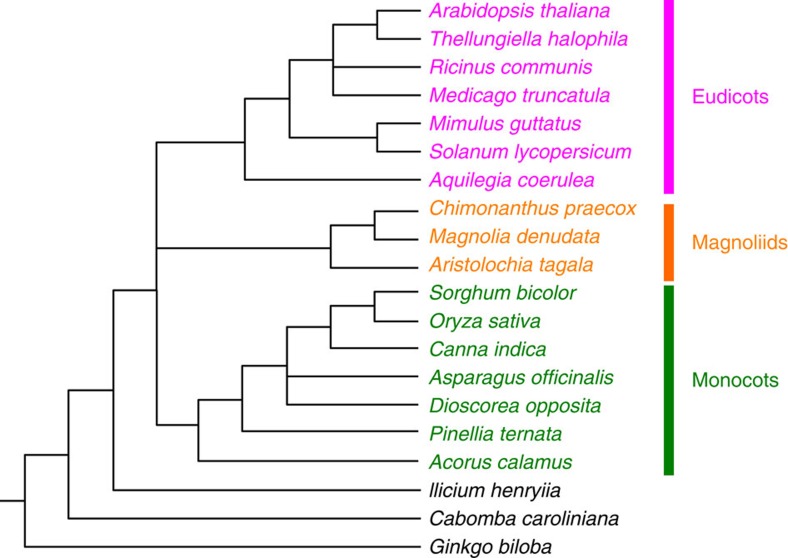
A cladogram depicting established relationships of 20 representative species. This tree was used as the reference for selecting suitable nuclear gene markers, with uncertain relationships collapsed.

As the amount of phylogenetic information varied among these 59 genes, we also ranked them based on the extent of the congruence between the single-gene tree of 20 representative species and the corresponding species tree (Fig. 2). Starting from the highest ranked gene (Supplementary Data 4), more genes were concatenated successively. Phylogenetic analyses suggested that only 16 genes (8,977 amino acids) were needed to resolve the mesangiosperm relationships, with the bootstrap value (BS) and PP for these 5 groups being at least 82% and 1.0, respectively (Fig. 4b and Supplementary Fig. 6). Therefore, even fewer carefully selected genes from sufficiently large taxon sampling can resolve some of the most difficult problems in angiosperm phylogeny. Furthermore, the use of low-to-moderate number of genes for phylogenetic analyses can decrease the time and cost of analysis, and is thought to reduce the chance of systematic errors^44^.

### The newly proposed topology is robust to sequence biases

It is possible that highly supported phylogeny could be in part due to artificial signals from species or genes with long branches and genes with saturated mutation^45^. To investigate effects of possible sequence biases in our phylogenomic data set on the topology, several related tests were performed using a software of the TreSpEx package^46^, which was recently developed for detecting possible sequence biases, including LBA and saturation. To detect long branches, this programme defined a measure called Long Branch (LB) score, which is calculated from patristic distance (PD) values between taxa in a gene tree (see Methods), where PD is the sum of lengths of branches that link two nodes in a tree^47^.

**Figure 3:**
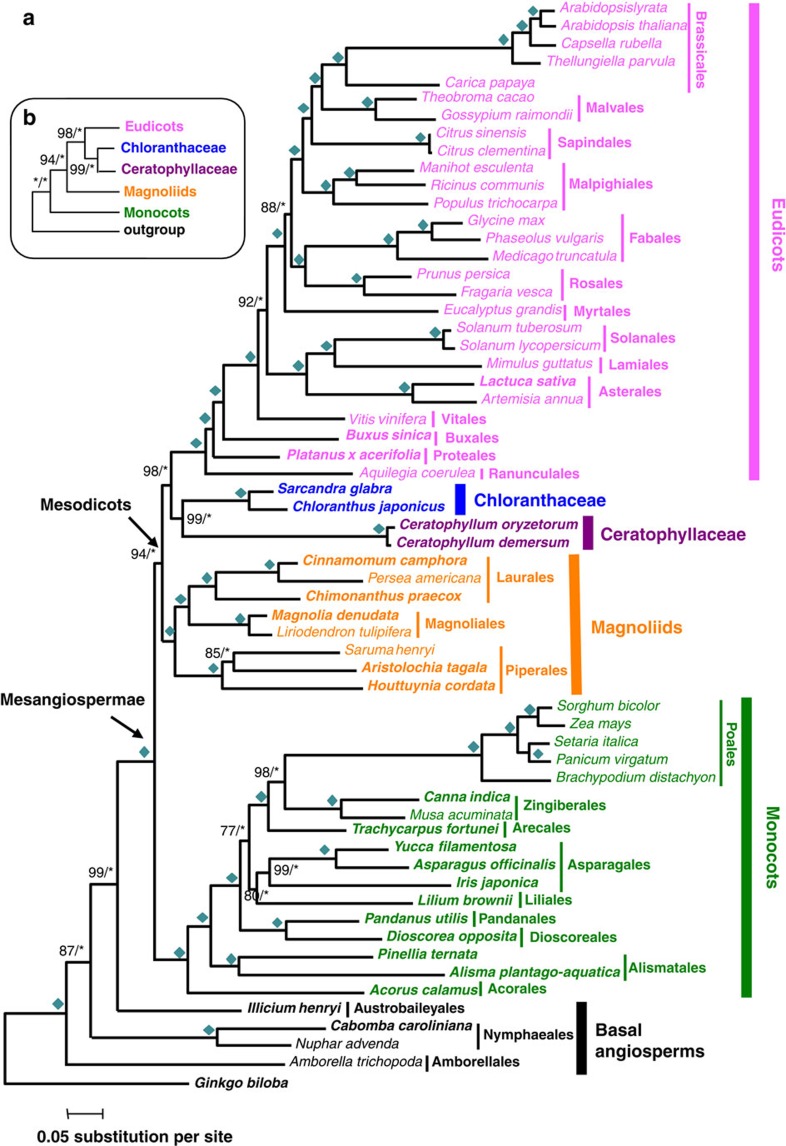
A highly supported hypothesis for relationships among the five major groups of Mesangiospermae. (**a**) A phylogeny of 61 taxa using 59 genes. Numbers associated with nodes are the bootstrap value (BS, on the left) obtained by RAxML and the posterior probability (PP, on the right) obtained by MrBayes based on concatenated 59 genes. Asterisks indicate either BS of 100% or PP of 1.0. Diamonds indicate both BS of 100% and PP of 1.0. Taxa with transcriptome data specifically generated for this study are shown in bold. (**b**) An abbreviated tree showing the relationship of the five lineages of Mesangiospermae, with associated support values (BS/PP).

**Figure 4:**
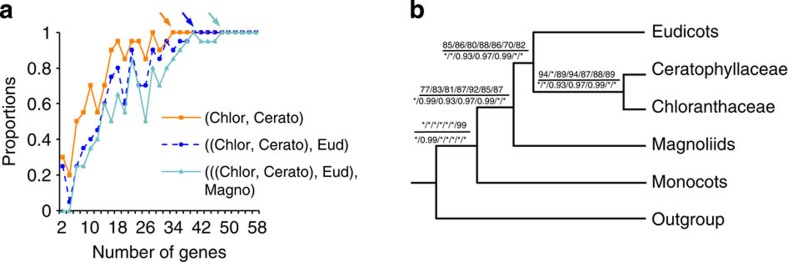
The effect of the number of genes on the phylogenetic topology. (**a**) percentages (*y* axis) of trees with the same topology as that in Fig. 3, when 20 trees were generated using the number (*x* axis) of randomly selected genes. The minimal number of genes required for 100% recovery of the (Chlor, Cerato), ((Chlor, Cerato), Eud) and (((Chlor, Cerato), Eud), Magnoliids) clades was 34, 40 and 48, respectively, as indicated by arrows. (**b**) A cladogram inferred by different numbers of ranked genes. Numbers associated with nodes are the bootstrap value (BS, above the line) obtained by RAxML and the posterior probability (PP, below the line) obtained by MrBayes with 55, 50, 46, 41, 33, 25 and 16 genes (right to left), respectively. BS of 100% or PP of 1.0 is indicated by asterisk. Chlor, Cerato, Eud, Magno, Mon and Out represent Chloranthaceae, Ceratophyllaceae, eudicots, magnoliids, monocots and outgroup, respectively.

To detect possible genes with long branches in our data set, gene-specific LB scores were calculated for each taxon and used to determine two additional parameters, which are the mean value of upper quartile and the s.d. of these LB scores. The distribution of these parameters appeared nearly normal, with a few additional small peaks (Supplementary Fig. 7). In the distribution of the mean value of upper quartile of LB scores, the skewed part contains two small peaks (marked in red; Supplementary Fig. 7a), corresponding to four genes; similarly, the distribution for the s.d. of LB scores had several small peaks in the skewed part (marked in red; Supplementary Fig. 7b), matching eight genes, which included the four genes identified in distribution shown in Supplementary Fig. 7a. The removal of only the shared four or all eight long-branch genes from the 59-gene data set resulted in the same topology as that in Fig. 3, with only slightly decreased BS values at some nodes (Supplementary Fig. 8).

To identify genes that behaved as long branches in specific taxon, a heatmap of taxon-specific LB scores for each gene was generated. Three gene sequences, one from each of three taxa (*Carica papaya*, *Amborella trichopoda* and *G. biloba*), were found to have the highest LB scores (above 150) and considered most likely to exhibit long-branch characteristics (Supplementary Fig. 9). After pruning these three sequences, the topologies of angiosperms obtained by RAxML and MrBayes were completely identical with that shown in Fig. 3 (Supplementary Fig. 10). BS values of Mesodicots and for the sisterhood between eudicots and Chloranthaceae/Ceratophyllaceae declined, respectively, from 94% to 90% and from 98% to 96%. However, BS values for the sisterhood of Chloranthaceae/Ceratophyllaceae and *A. trichopoda* being sister to all other angiosperms increased from 99% and 87% to 100% and 96%, respectively. Moreover, BS values increased for four nodes in eudicots and monocots. These results suggested that the genes or taxa with long-branch characteristics were not the primary cause of our topology.

Fast-evolving sites in gene sequences are likely to have repeated substitutions during evolution, resulting in mutational saturation, which could affect phylogenetic reconstruction^45^,^48^. The degree of gene saturation can be determined based on the linear regression of PD and uncorrected distance *p*^46^. The *p* distance counts the number of differences in sequences without considering multiple changes^47^. The linear regression of each gene was analysed either by the slope or *R*^2^ values, yielding a nearly normal distribution of the slope and *R*^2^ values of 59 genes with small peaks on the left-hand side. The two small peaks for the distribution of slopes contained five genes and the skew part of *R*^2^ comprises two genes, which were among the five genes (Supplementary Fig. 11). In addition, these two genes identified as being saturated by both the analyses of slope and *R*^2^ values were also among the four genes that exhibited long-branch characteristics in both analyses as described above (Supplementary Fig. 7). Matrices after excluding all 5 or only 2 genes from the 59-gene data set produced the same topology as the one in Fig. 3, with slightly altered BS values of the 5 major angiosperm groups (Supplementary Fig. 12).

### The topology is robust to sampling

To evaluate the influence of sampling on phylogenetic reconstruction, several analyses were performed using concatenated 59-gene from different subsets of taxa. Small sister lineages of other larger lineages within major groups are known to be crucial for phylogenetic reconstruction, as illustrated here by the removal of three Piperales species in magnoliids (Supplementary Fig. 13a) or three basal monocot species (Supplementary Fig. 13b). The matrices after such exclusions generated the same topology as shown in Fig. 3, with Bayesian PP values remaining at least 0.99. However, the BS values associated the sisterhood between eudicots and Chloranthaceae/Ceratophyllaceae decreased from 98% to 65%, indicating that sufficient taxon sampling, especially those smaller sister lineages, was vital to obtain a well-resolved phylogeny. Adding three eudicot species (*Nelumbo necifera, Eschscholzia californica* and *Meliosma arviflora*), which are sister to major eudicot groups also resulted in the same topology as shown in Fig. 3 (Supplementary Fig. 13c). Moreover, three Pinaceae species (*Picea sitchensis*, *Picea glauca* and *Pinus taeda*), representatives of the largest gymnosperm clade, were added to the outgroups and the topology of angiosperm species was identical with the one in Fig. 3, with slightly decreased BS values associated with the five major groups in Mesangiospermae (Supplementary Fig. 13d).

### Conflicting signals detected from plastid genome datasets

To investigate possible factors for the difference between the APG III topology from chloroplast markers for the five major groups and the hypothesis presented in this study, we re-examined the data sets from 83 plastid genes of 86 species^16^ and 40 genes from the plastid IR of 244 species^17^. As mentioned above, topologies inferred by these two plastid data sets were different, with the former (83 genes of 86 species) (Supplementary Fig. 14a) congruent with the APG III one and the latter (40 IR genes of 244 species) identical to the one in this study. To facilitate the comparison between these two data sets, the matrix composed of the 40 IR genes of the same 86 species sampled in the study of 83 plastid genes was analysed. First, the percentage of parsimony informative (PI) sites of each gene was estimated and found to vary dramatically, ranging from 2.8% (*trnl*.GAU) to 64.3% (*ycf1*) for the 40 IR genes (Supplementary Table 3) and from 11.2% (*rrn16*) to 77.3% (*matK*) for the 83 plastid genes (Supplementary Table 4). The average percentage of PI of IR gene (34.9%) was lower than that of single-copy plastid genes (46%), suggesting that IR genes as a group were more conservative than single-copy plastid genes and might be less affected by mutational saturation^17^.

We then tested whether different topologies could result from the use of plastid genes with different evolutionary rates. The 40 IR genes were classified into slower genes and faster genes using three different cutoff percentages of PI, that is, 30%, 40% and 50% (Supplementary Table 5), generating six matrices: IR_less_30, IR_greater_30, IR_less_40, IR_greater_40, IR_less_50 and IR_greater_50. We found that for IR genes, in spite of large differences between the average percentages of PI of slower versus faster genes, that is, 16.6% versus 47.9% (IR_less_30 versus IR_greater_30), 21.1% versus 51.2% (IR_less_40 versus IR_greater_40) and 26.6% versus 54.6% (IR_less_50 versus IR_greater_50), the topologies are completely identical with the one using all 40 IR genes regarding the relationships among the 5 major groups (Supplementary Table 5 and Supplementary Fig. 14b), suggesting that the single-gene percentage of informative sites did not affect the topology. Nevertheless, the supporting values generally increased when more PI sites were included (Supplementary Table 5).

Similarly, 6 matrices were generated from 83 plastid genes using 3 cutoff values of the percentage of PI of 40%, 50% and 60%, respectively, and were named SC_less_40, SC_greater_40, SC_less_50, SC_greater_50, SC_less_60 and SC_greater_60. For these 83 plastid genes, the topology changed when using different gene sets. Specifically, when conserved genes (with lower PI values) were used, eudicots, Ceratophyllaceae and monocots grouped together with moderate support values (77% to 88% BS), but the relative positions of Chloranthaceae and magnoliids varied (Supplementary Fig. 14c–e). However, relatively rapidly evolving genes (higher PI values) gave different results: 17 (SC_greater_60) and 37 (SC_greater_50) genes yielded a sisterhood of Chloranthaceae and Ceratophyllaceae with the BS values of 25% and 79%, respectively (Supplementary Fig. 14f,g), but 49 genes (SC_greater_40) resulted in Chloranthaceae being sister to magnoliids with the BS value of 50% (Supplementary Fig. 14h). These results suggested that conflicting phylogenetic signals exist among these 83 genes.

To further investigate the source of conflicting signals, two new data sets were generated and were named SC_40_PI_50 (for genes with PI values between 40 and 50) and SC_50_PI_60. The SC_40_PI_50 matrix yielded a topology with eudicots, Ceratophyllaceae and monocots in a clade with 69% BS support (Supplementary Fig. 14i), but the SC_50_PI_60 matrix grouped Chloranthaceae and Ceratophyllaceae together with a BS value of 85% (Supplementary Fig. 14j). We also concatenated 40 IR genes and 72 plastid single-copy genes obtained by excluding 11 overlapping IR genes from 83 plastid gene data set; the length of the alignment reached 76,014 bp, yielding a topology of the 5 major groups that was congruent with that was generated using the 83 plastid genes (Supplementary Fig. 14k), but the BS values decreased slightly, again suggesting that conflict signals exist between single-copy genes and IR genes, and that IR genes and single-copy plastid genes possibly have different evolutionary histories. Therefore, when conflicting signals exist, simply increasing the number of genes could not resolve the difficult question of mesangiosperm phylogeny.

### Morphological characters revisited using the new topology

**Figure 5:**
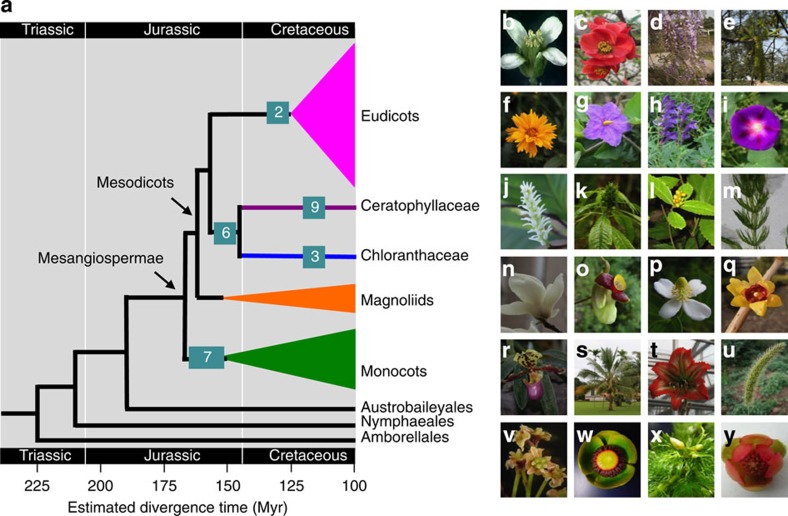
Estimated divergence times of Mesangiospermae and shared morphological characters. (**a**) The estimated divergence time for each node is shown relative to the geological time scale below the cladogram. Numbers in the rectangles on some branches indicate the numbers of morphological characters shared by the corresponding lineages. (**b**–**y**) Plant photographs show the diversities of angiosperms; (Rosids: *A. thaliana* (**b**), *Chaenomeles cathayensis* (**c**), *Wisteria sinensis* (**d**), *Juglans regia* (**e**); Asterids: *Coreopsis basalis* (**f**), *Solanum wrightii* (**g**), *Scutellaria baicalensis* (**h**), *Pharbitis purpurea* (**i**); Chloranthaceae: *Chloranthus japonicus* (**j**), *Hedyosmum orientale* (**k**), *Sarcandra glabra* (**l**); Ceratophyllaceae: *Ceratophyllum demersum* (**m**); Magnoliids: *Magnolia denudata* (**n**), *Aristolochiae heterophyllae* (**o**), *Houttuynia cordata* (**p**), *Chimonanthus praecox* (**q**); Monocots: *Paphiopedilum henryanum* (**r**), *Cocos nucifera* (**s**), *Hippeastrum rutilum* (**t**), *Setaira viridis* (**u**); Basal angiosperms: *A. trichopoda* (**v**), *Nuphar advena* (**w**), *Cabomba caroliniana* (**x**), *Schisandra sphenanthera* (**y**).

Recently, Endress and Doyle^43^ reconstructed the angiosperm phylogeny using a morphological data set composed of 110 characters, and they proposed that Ceratophyllaceae might be sister to Chloranthaceae (Fig. 1d). The topology with high support values uncovered here provides a new opportunity to examine the evolution of morphological characters. Compared with the minimum (775) steps required for the Endress and Doyle’s^43^ topology obtained by morphological analyses (Fig. 1d), 777 steps were needed for our best maximum likelihood (ML) tree (Table 2), suggestive of the strong agreement between our topology and the morphological data set, whereas 785 steps were required for the topology accepted by APG III (Fig. 1a), 10 more steps than the most parsimony one. The sisterhood of Chloranthaceae and Ceratophyllaceae was supported by six characters originated before their diversification (Fig. 5 and Supplementary Fig. 15) in the context of our topology. In contrast, only one (dry fruit wall) supported the alternative hypothesis of Ceratophyllaceae and eudicots being sisters^43^ and only the loss of cambium associated Ceratophyllaceae with monocots. The Chloranthaceae–Ceratophyllaceae sisterhood was also uncovered using other molecular data sets, albeit with low support values^15^,^17^,^40^. In addition, seven characters including absence of cambium (4), parallel major venation (17), boat-shaped pollen (61) and one cotyledon (110), originated before the origin of monocots but after the diversification of monocots from Mesodicots, enabling relatively easy differentiation between these groups (Fig. 5 and Supplementary Fig. 16) and consistent with the well-known distinctive morphologies of monocots. Two, three and nine morphological characters could be interpreted as novel before the origin of, respectively, the eudicots, Chloranthaceae and Ceratophyllaceae (Fig. 5 and Supplementary Figs 17–19). In contrast, no novel character was found for the magnoliids, providing an explanation for the previous idea that magnoliids were early angiosperms in classical taxonomy mainly according to morphological characters.

The plicate and completely sealed postgenital carpel (75 and 76) (Supplementary Fig. 20) might have originated before the origin of mesangiosperms, which could have enabled mesangiosperm species to produce more seeds or better protect the developing seeds when compared with most ANITA species with only ascidiate and not postgenitally sealed carpels. On the other hand, in the context of the topologies obtained from morphological data (Fig. 1d) or plastid genome data sets (Fig. 1a), this morphological novelty would have originated not before but after the origin of mesangiosperms (Supplementary Figs 21 and 22). These differences suggest that the phylogenetic relationship is critical for inferring ancestral characters and the topology here provides a potential new framework to investigate the evolution of these and other morphological, developmental and physiological characters.

### Possibly early origins of angiosperms and mesangiosperms

**Figure 6:**
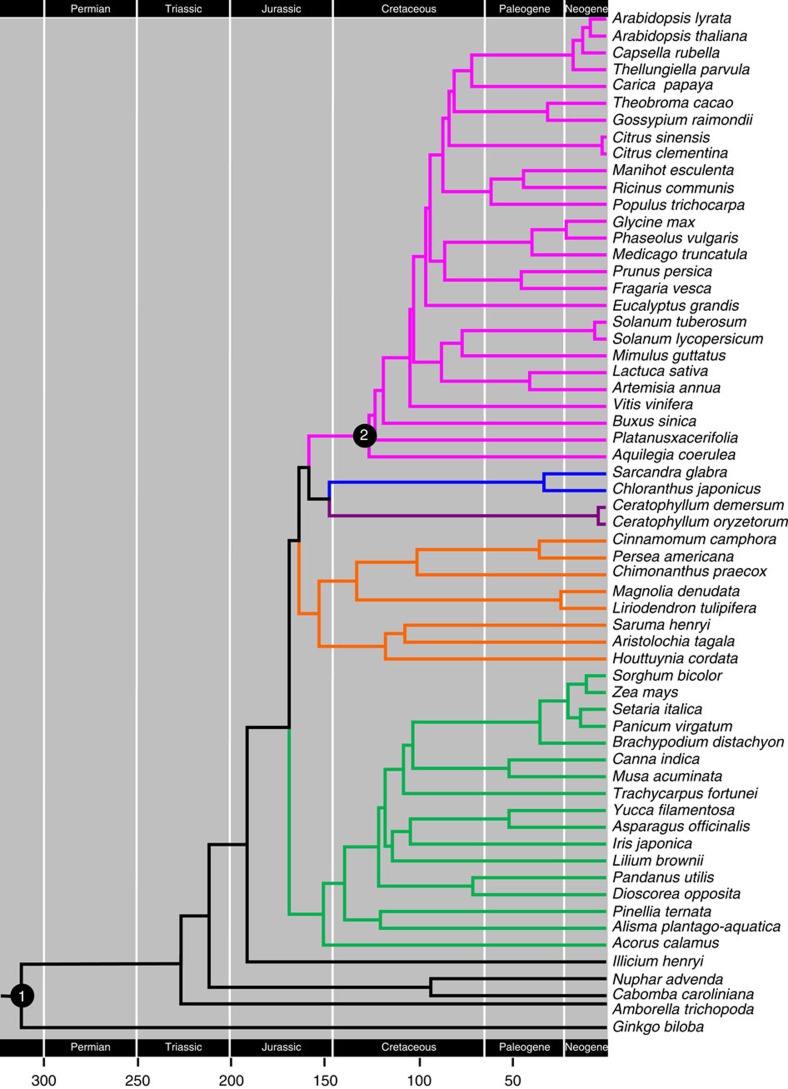
A chronogram showing the angiosperm divergence times as estimated by the BEAST using 59 genes. Two fossil calibration points: (1) the earliest gymnosperm fossils (*ca*. 290–310 Myr) and (2) the earliest fossil tricolpate pollen (~125 Myr) were marked with two solid circles.

According to the well-supported mesangiosperm topology and other deep relationships, a framework is proposed for estimating the divergence times of angiosperms, particularly for the five mesangiosperm groups, providing possible geological contexts of their rapid radiation. In general, times inferred by r8s^49^ were somewhat earlier (~10 million years, hereafter Myr) than those obtained by BEAST^50^ and only small differences were detected when using different codon positions (Supplementary Table 6), suggesting that time estimations were robust to methodology and data used. The origin of angiosperm was estimated to be 225–240 Myr, that is, in the Late-to-Middle Triassic (Figs 5 and 6, and Supplementary Table 6), considerably earlier than the previously accepted 140–180 Myr^21^, but in agreement with those recently reported independently^20^,^51^. An earlier angiosperm origin further expands the large gap between the origin of angiosperm and the earliest undisputed angiosperm fossil found in Hauterivian^52^; thus, possibly the oldest crown angiosperm fossils are yet to be discovered. Recently, angiosperm-like pollen grains were found in the Middle Triassic, consistent with our hypothesis of earlier origin of flowering plants; however, such an early origin is still controversial because of the lack of unequivocal meso-macro fossils (for example, fossilized flowers) at that time^53^. The diversification of Mesangiospermae was estimated to have initiated in the Jurassic (154–191 Myr) (Figs 5 and 6, and Supplementary Table 6), *ca*. 60 Myr after the angiosperm origin, consistent with an early origin of Mesangiospermae tentatively proposed by Smith *et al.*^20^ and Magallon^51^, but earlier than the 144 Myr estimated using whole plastid genome data^12^. Since the origin of Mesangiospermae, during an ~20-Myr period (instead of the 4 Myr based on plastid genomes^12^), the five major groups diversified successively as indicated by the well-resolved topology here, making the radiation less rapid than previously thought.

Strikingly, the time of angiosperm origin estimated here overlaps with the origin of several insect lineages. Curculionoidea (weevils and bark beetles) and Chrysomeloidea (leaf beetles and long-horned beetles), two important groups of plant feeders, were estimated to have originated ~230 Myr; meanwhile, fossils of Diptera (flies) and Hymenoptera (bees and wasps), the most important pollinators, were also found in Late Triassic^20^,^54^. Moreover, the rapid expansion of Mesangiospermae is congruent with the radiation of Lopidoptera (butterflies and moths), Hymenoptera (bees, ants and pollen wasps) and many kinds of flies from Late Jurassic to Early Cretaceous^20^,^23^,^54^. The coincidence in geological time of the mesangiosperm lineages and pollinating insects might provide opportunities for investigating the diversification of Mesangiospermae. We also noted that the extant crown eudicots diversified since ~35 Myr after their separation from the clade of Chloranthaceae and Ceratophyllaceae, suggesting potential extinctions of the stem relatives or a relatively long period of ‘stasis’ before their rapid diversification into the most successful plant group.

## Discussion

In this study we showed that the combination of a moderate number of carefully evaluated nuclear genes with appropriate sampling could provide robust and highly supported relationships among deep lineages of Mesangiospermae. These relationships differed substantially from those accepted by the APG III system. The topology here provides a new phylogenetic framework for ancestral character reconstruction, molecular clock estimates of divergence times and other studies, suggestive of the necessity and importance of conserved low-copy nuclear genes for evolutionary studies.

The topology obtained here is different with the one inferred by using single-copy plastid genes but congruent with the one obtained by highly conserved genes from the plastid IR region, indicating that the evolutionary histories of IR and single-copy plastid genes might be different. The evidence presented here for conflicting signals among single-copy plastid genes and between single-copy and IR plastid genes further suggests that some of the single-copy plastid genes might be unsuitable for resolving the deep relationships of angiosperms, as almost all single-copy genes have been used and conflicting signals exist. Conflicts between plastid and nuclear genome about the position of Malpighiales, Cornales and Ericales also suggested that evidence from nuclear genes is necessary^15^. With rapid advances in sequencing technologies and decreasing cost, nuclear genes will probably be used more and more in molecular phylogeny.

The early origin and diversification of angiosperms proposed here were also supported by two other recent independent studies^20^,^51^; all of these proposed origins were earlier than previous estimates, providing a new temporal framework for the evolution of angiosperms and ecologically related organisms. The possible coincidental origins and divergence of major angiosperm lineages with those of major pollinator insects provide a possible environmental factor that might have contributed to the rapid diversification of mesangiosperm lineages referred to by Darwin as the ‘abominable mystery’. In addition, our results demonstrate that ‘bushes’ in the tree of life from rapid radiations can be resolved by using a moderate number of nuclear genes^55^, which can be identified by careful screening of probable orthologues from transcriptomes of representative taxa, a strategy generally applicable to other phylogenetic questions.

## Methods

### Taxon sampling and data collection

Young leaves or flower buds of 26 species (see details in Supplementary Tables 1 and 2) were collected and frozen at −80 °C. Total RNA was extracted by a modified CTAB method^15^ and then paired-end reads of 2 × 100 were generated using the Illumina technology with HiSeq2000 (Table 1). Short reads were assembled into longer contigs *de novo* using Trinity^56^ (trinityrnaseq_r2012-06-08) with default parameters. For longer and more complete complementary DNA sequence, TGICLv2.1 (ref. 57) was also used with the parameter being –*P*=0.98, −l=40 and −v. Thirty sequenced genomes and five EST data sets were respectively retrieved from http://www.phytozome.net/search.php and ftp://ftp.ncbi.nih.gov/repository/UniGene/ (Supplementary Table 1).

### Orthologue identification and gene selection

To identify probable orthologous genes for phylogenetic analyses HaMStR was used, as it performs well in identification of orthologues from EST and RNA-seq data^35^, and its utility has been tested in previous phylogenomic studies of plants^32^ and animals^30^. First, we downloaded from the Deep Metazoan Phylogeny ( http://www.deep-phylogeny.org/hamstr/) 4,180 OGs, which were previously generated by comparing 9 angiosperm species with sequenced genome (*A. thaliana*, *Glycine max*, *Medicago truncatula*, *Populus trichocarpa*, *Solanum lycopersicum*, *Vitis vinifera*, *Oryza sativa*, *Sorghum bicolor* and *Zea mays*)^35^. These 4,180 OGs were then compared with 1,989 OGs identified from the analysis of seven whole-sequenced genomes using OrthoMCL (*A. thaliana*, *P. trichocarpa*, *Prunus persica*, *Mimulus guttatus, V. vinifera*, *S. bicolor* and *O. sativa*), resulting in 931 OGs that overlapped between these two data sets. HMM files of 931 OGs distributed with HaMStR were used to search for corresponding orthologues from other species with the parameter being -est, -hmmset=magnoliophyta_hmmer3, -relaxed, -eval_limit=0.01. To identify marker genes with sufficient coverage among the taxa, OGs were selected with putative orthologues found in 80% of the 26 species with newly generated RNA-seq datasets (Table 1); in addition, only sequences of coding regions with the length 80% of the *A. thaliana* homologue were retained for further analyses, ultimately resulting in 349 OGs.

Angiosperms have probably experienced a number of WGDs and subsequent gene losses^32^,^33^, making it difficult to identify orthologues. Because of the recently identified WGD before the divergence of all extant angiosperms and all seed plants, strictly defined orthologues that never experienced any duplication probably do not exist in angiosperms. Nevertheless, those genes that experienced rapid loss of one paralogue before the divergence of the species of interest can be considered as orthologues operationally. Therefore, we searched for low-copy genes and follow well-supported established organismal history. To minimize the effect of hidden paralogues^58^ and identify the most probable orthologues, 349 single-gene trees were reconstructed using RAxML^36^ with protein sequences of 20 representative species with well-supported relationships (Fig. 2), with the evolutionary model for each gene estimated by ProtTestv2.4 (ref. 59). Next, these gene trees were compared with the species tree.

As the informative sites of one gene are limited, it was difficult to resolve relationships among low-level taxonomic hierarchies using only one gene. Therefore, if genes of the same OG from species of a monophyletic organismal group (that is, eudicots, monocots and magnoliids) form a monophyletic gene clade, as they should, the gene was selected for further analyses; in contrast, if genes of the same OG from species of different monophyletic groups are in a supported gene clade, then this OG was excluded (see examples of ‘selected’ and ‘excluded’ genes in Supplementary Fig. 3). After careful examination, 54 genes were selected for further analyses. Combined with five genes (*SMC1*, *SMC2*, *MCM5*, *MSH1* and *MLH1*) effectively used previously^15^, a total of 59 genes was used for investigating the relationship among Mesangiospermae. Characteristics of these 59 genes, including functional annotation and percentages of PI sites are shown in Supplementary Table 2. Gene copy number detected in each species with whole sequenced genomes is listed in Supplementary Data 1; in species with more than one copy, the paralogues were found to represent terminal branches from recent duplications. The length of protein sequences encoded by orthologous genes in each species is listed in Supplementary Data 2.

### Phylogenetic analyses

Amino acid sequences of each OG were aligned using MUSCLE v3.8.31 (ref. 60) with default settings, the alignments were manually inspected to delete sequences of low quality, then the poorly aligned regions were further trimmed by using trimAl v1.2 (ref. 61). Single-gene trees were reconstructed with RAxML using the fittest evolutionary model inferred by ProtTest v2.4 (ref. 59). In species with two or more copies in one OG, the paralogues from recent duplication formed adjacent terminal branches in the gene tree; thus, only the gene with the shortest branch was retained for further analyses. Finally, amino acid sequences of 59 genes from 61 species were concatenated by SeaView^62^ and the length of the concatenated 59-gene amino acid matrix reached 25,589 amino acids.

ML and Bayesian trees based on the 59 protein sequences of 61 species were inferred by RAxML and MrBayes 3.2.1 (ref. 37), respectively. For ML analysis, the model was specified as JTT+I+G based on the results of ProtTest and fast BS analyses were replicated for 100 times. For Bayesian analysis, one cold and three incrementally heated Markov chain Monte Carlo chains were run simultaneously with the JTT model. The Markov chain Monte Carlo convergence in Bayesian phylogenetic inference was monitored by AWTY ( http://ceb.csit.fsu.edu/awty)^63^. Trees were sampled per 100 generations. The first 25% trees were discarded as burnin, with the remaining trees being used for generating the consensus tree.

To determine statistic support for other possible alternative relationships among the five major groups of Mesangiospermae (eudicots, monocots, magnoliids, Chloranthaceae and Ceratophyllaceae), all 105 potential topologies were tested using our data set (Table 2 and Supplementary Data 3). First, per site log likelihoods for each topology were estimated by RAxML under the JTT+I+G model, and then approximately unbiased test was conducted using CONSEL v1.20 (ref. 42).

To explore the minimal number of genes needed to resolve the relationship among the five major clades of Mesangiospermae, the relationship between the number of genes and the proportion of gene trees supporting the topology shown in Fig. 3 was studied. The number of genes ranged from 2 to 58, with increments of 2; for each number, 20 replicates of randomly selected genes were performed using the *sample* function implemented in R, generating a total of 580 matrices. Gene tree was inferred by RAxML with the model being JTT+I+G and the fast bootstrap replicate was set to 100.

As the phylogenetic information varies among the 59 genes, we also ranked them based on the extent of the congruence between the single-gene tree of 20 representative species and the corresponding species tree (Fig. 2). First, these single-gene tree was treated as condensed tree with the cut-off BS values being 50%; if the position of one species in single-gene tree is congruent, conflict or uncertain with the species tree, it was scored as 1, −1 and 0, respectively. Then, scores of all nodes from one single-gene tree were summed up and then 59 single-gene trees were ranked by their scores, with the gene with the highest being considered the best (Supplementary Data 4). Starting with 16 genes that had the highest scores, additional genes were added successively with total scores from high to low, resulting in matrices composed of 16, 25, 33, 41, 46, 50 and 55 gene sequences (Supplementary Data 4); finally, ML and Bayesian trees were inferred using RAxML and MrBayes, respectively, with same settings as described above.

To evaluate the effects of different evolutionary models on the species topology, ML trees using models other than the fittest one (that is, JTT+CAT, JTT+G, WAG+CAT and DAYHOFF+CAT) were inferred by RAxML.

### Detection of possible sequence biases

To investigate possible effects of sequence biases in our phylogenomic data set on the phylogenetic reconstruction, several related tests were performed using TreSpEx, which was recently developed for detection of possible sequence biases, including LBA and saturation^46^.

To detect long branches, this programme defined a parameter called LB score based on PD values between taxa in a gene tree, where PD is the sum of lengths of branches that link two nodes in a tree. For each gene, the mean pairwise PD of taxon i to all other taxa and the average pairwise PD across all taxa in the single-gene tree were estimated. The LB score of taxon i (LB_i_) in each gene was then determined by the relative value of the mean PD of taxon i to the average PD across all taxa. Fifty-nine single-gene trees were used as input files for TreSpEx. TreSpEx then provided two parameters derived from LB score for each gene: the average of upper quartile of LB scores and the s.d. of LB scores for comparisons between genes. Density plots (distribution) of these two parameters of 59 genes were generated with the R programme and shoulder areas deviated from the normal distribution were filled with red. Genes whose mean values of upper quartile or the s.d. of LB scores were found in right shoulders were considered as having long branches. Next, these long-branch genes were excluded from the 59-gene amino acid matrix and the ML tree was inferred by RAxML as mention above. In addition, to identify genes that behaved as long branches in a specific taxon, taxon-specific LB scores for each gene were calculated and a heatmap of these scores was generated with hierarchical clustering. Genes with the highest LB scores (above 150), were pruned from our data set and the remaining sequences were used to reconstruct the ML tree using RAxML with the model being JTT+I+G.

The degree of saturation of each gene can be determined using the linear regression of PD and uncorrected distances *p*. The *p* distance is the number of difference in sequences without considering multiple changes^47^. The linear regression of each gene was estimated either by the slope or *R*^2^ values. Fifty-nine single-gene trees and alignments of 61 species were used as input files for TreSpEx. First, PD matrix and *p* matrix of each gene were respectively calculated and the linear regression of them was generated for each gene. Second, distributions of the slope or *R*^2^ values were plotted with the aid of R. The rationale of TreSpEx is that the better the fit to linear regression, or in another way, the larger the slope or *R*^2^ value, the less saturated the data. Genes located in left shoulders of the slope or *R*^2^ graphs, respectively, were pruned from the 59-gene data set and the ML tree was reconstructed with RAxML.

### Taxon sampling analyses

To evaluate the influence of taxa sampling on phylogenetic reconstruction, several analyses were performed using concatenated 59 genes from different subsets of taxa. Basal lineages of major groups are known to be crucial for reconstructing phylogeny^31^,^64^; hence, three Piperales species in magnoliids or three basal monocot species were deleted. ML and Bayesian trees using the pruned matrices were reconstructed. To test whether additional representatives of small eudicot groups that are sisters to major eudicot groups could affect the topology, we added orthologous genes from the recently sequenced genome of *N. necifera* (Proteales)^65^, publicly available transcriptome of *E. californica* (Ranunculales) from NCBI ( http://www.ncbi.nlm.nih.gov/SRA) and the transcriptome of *M. arviflora* (Sabiaceae) that we recently obtained.

Moreover, three Pinaceae species (*P. sitchensis*, *P. glauca* and *P. taeda*), representatives of the largest clade of gymnosperms, were included. EST data sets of these three species were downloaded from NCBI ( ftp://ftp.ncbi.nih.gov/repository/UniGene/). Orthologues of 59 genes from these three species were obtained by HaMStR as described above, then ML and Bayes tree were inferred using RAxML and MrBayes, respectively.

### Reconstruction of angiosperm phylogeny using plastid genomes

To investigate possible factors on the difference between the APG III topology from chloroplast markers for the five major groups and the hypothesis presented in this study, we re-examined the data sets from 83 plastid genes of 86 species and 40 genes from the plastid IR of 244 species. First, the percentage of PI sites of each gene was estimated by PAUP^66^. Next, ML trees using plastid genes with different percentage of informative sites were inferred by RAxML with the model being GTRCAT, and the BS analysis was repeated 100 times.

### Re-analysis of 110 morphological characters

One hundred and ten morphological characters sorted by Endress and Doyle^43^ were reinvestigated with Mesquite (version 2.75)^67^. As the five major groups of Mesangiospermae were also sampled in their study, we simply tested alternative relationships among the five major lineages based on their samplings. First, the minimum step required for each of 105 possible topologies was inferred using the parsimony model. Second, ancestral states of each character were inferred using the likelihood model in the context of our topology (Fig. 3) to detect potential synapomorphies. As polymorphic or uncertain taxa are not supported by likelihood model, those taxa with uncertain character and missing data were removed from the matrix for each character. Then, ancestral states with probabilities were obtained. To detect the novel character specific to one group, for example, the ancestral state of the eudicots was compared with the one of the upper node connecting both eudicots and the clade of Chloranthaceae and Ceratophyllaceae. If the ancestral states of these two nodes are supported significantly and represent different state, then there was a novel character before the origin of eudicots. To test whether different topologies could affect the ancestral reconstruction of morphological characters, the ancestral state of each character was also inferred in the context of other two topologies, that is, the one obtained by Endress and Doyle^43^ based on morphological data (Fig. 1d) and the one accepted by APG III (Fig. 1a).

### Divergence time estimation

For divergence time estimation, besides 61 species used for phylogenetic analyses, *Selagenella moellendorffii*, a basal vascular plant with a sequenced genome, was also included. *S. moellendorffii* is helpful for determining the crown node of seed plants, which is critical as a deep fossil constrained node and has been widely used in previous dating analyses^12^. The *S. moellendorffi*i orthologues of the 59 genes were retrieved using HaMStR as described above.

We estimated the divergence times based on three sets of data partitions, data consisting of the first codon positions, the first and second positions and all three codon positions, respectively. Penalized likelihood (PL) implemented in r8s (v.1.7.1)^49^, and Bayesian relaxed clock in BEAST (v.1.7.5)^50^ were used to estimate the divergence times of extant angiosperm lineages, as constant substitution rate across the phylogenetic tree was rejected (*P*<0.01) for all three data partitions by likely ratio test conducted in PAUP 4.0 beta10 (ref. 66).

For the PL method, the ML tree with branch length generated by RAxML was used as the input tree. The topologies inferred by these three nucleotide matrices are different from the one shown in Fig. 3 (data not shown) regarding the relationships among the five major clades of Mesangiospermae, possibly because of mutation saturation and homoplasy. Therefore, when inferring the ML tree by RAxML, the constraint tree was given, which forced the relationships among the five major clades of Mesangiospermae as shown in Fig. 3.

The outgroup *Selagenellia* was pruned as required by r8s. Cross-validation was tested to determine the best smoothing value for our data. After testing a range of smoothing parameters from 0.01 to 320 (cvstart=−2; cvinc=0.5; cvnum=10), the smoothing parameter of all three codon positions, the first and second positions and the first codon position was set to 3.5, 10 and 10, respectively. These low smoothing values also indicate a large deviation from the strict molecular clock hypothesis. One hundred BS trees with branch length were also generated using RAxML, which were used as input trees to calculate the confidence time intervals. The s.e. and 95% confidence time interval of a few nodes of interest were estimated and summarized across the 100 BS trees. The algorithm of TN was used and all other parameters were set as default in all above PL analysis.

For the BEAST analysis, (UCLN) was used with nucleotide substitution model being GTR+I+Γ and Yule speciation was specified for all three matrices. Two independent replications each with 60,000,000 generations were run with sampling every 5,000 generations. The stationary of the chains and convergence of the two runs was monitored by Tracer (v. 1.5), determining whether the effective sample size of all parameters was larger than 200 as recommended. The files from two independent runs were combined using LogCombiner (v. 1.7.5). The chronogram with nodal heights and 95% confidence time intervals was generated with TreeAnnotator (v. 1.7.5), with the first 5,000 trees being discarded as burnin; finally, the chronogram was displayed by Figtree (v. 1.0).

The earliest gymnosperm fossils (*ca*. 290–310 Myr) assigned to cycads^68^ and conifers^69^, and the earliest fossil tricolpate pollen (~125 Myr) associated with eudicots^70^ were used as two calibration points. For the PL analysis, the node of crown seed plants was constrained with minimum age of 290 Myr and maximum age of 310 Myr, while the crown eudicots was treated as the fixed and the minimum age of 125 Myr, respectively. For the BEAST analysis, the crown seed plants was constrained using uniform distribution with lower bound of 290 Myr and upper bound of 310 Myr; the node for crown extant eudicots was constrained using a prior of exponential distribution with offset of 125 Myr and mean value of 1, respectively. Our major fossil constraints are comparable to those used for calibrating the chloroplast genome data in previous studies, except that several different additional internal fossils were also included in previous studies^12^,^16^.

## Author contributions

N.Z. and H.M. designed this study. N.Z. and L.Z. collected plant materials, extracted total RNA for next-generation sequencing and drafted the manuscript. L.Z., Q.Z., R.S. and N.Z. performed phylogenetic reconstruction and morphological analyses. H.M. and H.K. revised the manuscript. All authors contributed and approved the final manuscript.

## Additional information

**Accession Codes**: The nucleotide sequences of the 59 genes used in this study have been deposited in GenBank nucleotide database, with the accession codes KM397373 to KM400584. In addition, the nucleotide and amino acid sequence alignments of 59 genes have been deposited in the TreeBASE under accession code S16175.

**How to cite this article:** Zeng, L. *et al.* Resolution of deep angiosperm phylogeny using conserved nuclear genes and estimates of early divergence times. *Nat. Commun.* 5:4956 doi: 10.1038/ncomms5956 (2014).

## Supplementary Material

Supplementary Figures and Tables Supplementary Figures 1-22 and Supplementary Tables 1-6

Supplementary Data 1 Gene copy numbers of 59 OGs in 30 species with sequenced genomes.

Supplementary Data 2 The length of predicted proteins encoded by the 59 genes from each species.

Supplementary Data 3 AU tests for 105 alternative topologies among the five major clades of Mesangiospermae, p values > 0.05 are in bold.

Supplementary Data 4 Information of single gene trees and the genes used for reconstructing trees based on different number of ranked genes.

## Figures and Tables

**Table 1:** Information of 26 transcriptome data generated in this study.

**Species**	**Order**	**Major lineage**	**Tissue**	**Number of raw reads**	**Size of data**	**Number of contigs**
*Ginkgo biloba*	Ginkgoales	Gymnosperms	Leaf	25,756,816	2.09 G	89,648
*Cabomba caroliniana*	Nymphaeales	Basal angiosperm	Leaf	35,843,440	2.87 G	20,254
*Illicium henryi*	Austrobaileyales	Basal angiosperm	Leaf	28,445,320	2.28 G	144,464
*Acorus calamus*	Acorales	Monocots	Leaf	64,399,584	8.26 G	182,444
*Alisma plantago-aquatica*	Alismatales	Monocots	Leaf	52,452,528	4.39 G	94,685
*Pinellia ternata*	Alismatales	Monocots	Leaf	72,493,048	6.04 G	304,976
*Dioscorea opposita*	Dioscoreales	Monocots	Leaf	30,682,680	2.47 G	114,265
*Pandanus utilis*	Pandanales	Monocots	Leaf	54,034,776	4.49 G	118,755
*Lilium brownii*	Lilales	Monocots	Flower	66,861,752	5.39 G	157,629
*Asparagus officinalis*	Asparagales	Monocots	Inflorescence	33,527,664	2.75 G	139,497
*Iris japonica*	Asparagales	Monocots	Leaf	22,938,240	5.89 G	114,374
*Yucca filamentosa*	Asparagales	Monocots	Flower buds	29,266,264	2.40 G	191,522
*Trachycarpus fortunei*	Arecales	Monocots	Leaf	21,050,560	1.68 G	118,595
*Canna indica*	Zingiberales	Monocots	Leaf	33,063,440	2.67 G	171,513
*Houttuynia cordata*	Piperales	Magnoliids	Leaf	33,672,032	1.58 G	170,239
*Aristolochia tagala*	Piperales	Magnoliids	Flower buds	31,282,944	3.03 G	95,015
*Magnolia denudata*	Magnoliales	Magnoliids	Flower buds	22,911,816	1.79 G	211,180
*Chimonanthus praecox*	Laurales	Magnoliids	Flower buds	30,844,864	2.43 G	183,567
*Cinnamomum camphora*	Laurales	Magnoliids	Leaf	38,036,976	3.12 G	147,307
*Ceratophyllum demersum*	Ceratophyllales	Ceratophyllales	Leaf	48,430,944	3.81 G	225,862
*Ceratophyllum oryzetorum*	Ceratophyllales	Ceratophyllales	Leaf	17,721,456	4.55 G	119,258
*Chloranthus japonicus*	Chloranthales	Chloranthales	Inflorescence	24,913,272	1.98 G	158,266
*Sarcandra glabra*	Chloranthales	Chloranthales	Inflorescence	28,810,376	2.30 G	117,357
*Buxus sinica*	Buxales	Basal eudicots	Leaf	25,719,032	2.10 G	111,089
*Platanus* x *acerifolia*	Proteales	Basal eudicots	Leaf	29,498,128	2.34 G	164,918
*Lactuca sativa*	Asterales	Core eudicots	Leaf	59,666,810	2.90 G	107,051

**Table 2:** AU test and the minimum steps of 110 characters required by 15 published topologies.

**Tree**	**Topology**	**−lnL**	**Diff –lnL**	**AU**	**Steps**
Our tree	(((((Cerato,Chlor),Eud),Magnol),Mono),Out)	**−**801884.5732	(best)	**0.965**	777
2	(((Cerato,Chlor),((Mono,Magnol),Eud)),Out)	**−**801932.3642	51	**0.203**	775
3	(((Cerato,Chlor),((Mono,Eud),Magnol)),Out)	**−**801963.9212	83.1	0.036	775
4	(((((Cerato,Mono),Eud),Magnol),Chlor),Out)	**−**802010.0536	139.1	0.023	783
5	((((Cerato,Chlor),(Eud,Magnol)),Mono),Out)	**−**801945.2438	67.5	0.019	777
6	((((Cerato,Chlor),Magnol),(Mono,Eud)),Out)	**−**801982.6426	109.5	0.018	776
7	(((Cerato,Eud),((Chlor,Mono),Magnol)),Out)	**−**802062.7199	208.2	0.009	784
8	(((((Cerato,Chlor),Magnol),Mono),Eud),Out)	**−**801979.7486	105.9	0.008	776
9	(((((Cerato,Eud),Mono),Chlor),Magnol),Out)	**−**802061.6555	206.1	0.003	787
10	((((Cerato,Eud),Mono),(Chlor,Magnol)),Out)	**−**802038.5455	186.7	0.002	785
11	(((((Cerato,Eud),Magnol),Chlor),Mono),Out)	**−**802019.2713	158.1	0.001	787
12	(((Cerato,Mono),(Chlor,(Eud,Magnol))),Out)	**−**801995.197	139.3	5.00E−04	783
13	(((((Cerato,Eud),Magnol),Mono),Chlor),Out)	**−**802045.461	166.5	3.00E−04	784
14	((((Cerato,Mono),Chlor),(Eud,Magnol)),Out)	**−**802023.879	168.7	1.00E−06	782
15	(((Cerato,(Mono,Eud)),(Chlor,Magnol)),Out)	**−**802060.8392	210.8	3.00E−47	782

AU, approximately unbiased test; Cerato, Ceratophyllaceae; Chlor, Chloranthaceae; Eud, eudicots; Magnol, magnoliids; Mono, monocots.

*P*-values>0.05 are in bold.

## References

[b1] Judd W. S., Campbell C. S., Kellogg E. A., Stevens P. F. & Donoghue M. J. Plant Systematics: a Phylogenetic Approach Sinauer Associates (1999).

[b2] Friedman W. E. The meaning of Darwin’s ‘abominable mystery’. Am. J. Bot. 96, 5–21 (2009). 21628174 10.3732/ajb.0800150

[b3] Sun G., Dilcher D. L., Wang H. & Chen Z. A eudicot from the Early Cretaceous of China. Nature 471, 625–628 (2011). 21455178 10.1038/nature09811

[b4] Friis E. M., Pedersen K. R. & Crane P. R. Fossil evidence of water lilies (Nymphaeales) in the Early Cretaceous. Nature 410, 357–360 (2001). 11268209 10.1038/35066557

[b5] Sun G., Dilcher D. L., Zheng S. & Zhou Z. In search of the first flower: a Jurassic angiosperm, Archaefructus, from northeast China. Science 282, 1692–1695 (1998). 9831557 10.1126/science.282.5394.1692

[b6] Bremer B. *et al.* An update of the Angiosperm Phylogeny Group classification for the orders and families of flowering plants: APG III. Bot. J. Linn. Soc. 161, 105–121 (2009).

[b7] Qiu Y. *et al.* The earliest angiosperms: evidence from mitochondrial, plastid and nuclear genomes. Nature 402, 404–407 (1999). 10586879 10.1038/46536

[b8] Cantino P. D. *et al.* Towards a phylogenetic nomenclature of Tracheophyta. Taxon 56, 1E–44E (2007).

[b9] Cronquist A. An Integrated System of Classification of Flowering Plants Columbia University Press (1981).

[b10] Friis E. M., Pedersen K. R. & Crane P. R. Diversity in obscurity: fossil flowers and the early history of angiosperms. Philos. Trans. R. Soc. Lond. B Biol. Sci. 365, 369–382 (2010). 20047865 10.1098/rstb.2009.0227 PMC2838257

[b11] Dilcher D. L. & Wang H. An Early Cretaceous fruit with affinities to Ceratophyllaceae. Am. J. Bot. 96, 2256–2269 (2009). 21622341 10.3732/ajb.0900049

[b12] Moore M. J., Bell C. D., Soltis P. S. & Soltis D. E. Using plastid genome-scale data to resolve enigmatic relationships among basal angiosperms. Proc. Natl Acad. Sci. USA 104, 19363–19368 (2007). 18048334 10.1073/pnas.0708072104 PMC2148295

[b13] Qiu Y. *et al.* Phylogenetic analyses of basal angiosperms based on nine plastid, mitochondrial, and nuclear genes. Int. J. Plant Sci. 55, 837–856 (2005).

[b14] Qiu Y. *et al.* Reconstructing the basal angiosperm phylogeny: evaluating information content of mitochondrial genes. Taxon 55, 837–856 (2006).

[b15] Zhang N., Zeng L., Shan H. & Ma H. Highly conserved low-copy nuclear genes as effective markers for phylogenetic analyses in angiosperms. New Phytol. 195, 923–937 (2012). 22783877 10.1111/j.1469-8137.2012.04212.x

[b16] Moore M. J., Soltis P. S., Bell C. D., Burleigh J. G. & Soltis D. E. Phylogenetic analysis of 83 plastid genes further resolves the early diversification of eudicots. Proc. Natl Acad. Sci. USA 107, 4623–4628 (2010). 20176954 10.1073/pnas.0907801107 PMC2842043

[b17] Moore M. J. *et al.* Phylogenetic analysis of the plastid inverted repeat for 244 species: insights into deeper-level angiosperm relationships from a long, slowly evolving sequence region. Int. J. Plant Sci. 172, 541–558 (2011).

[b18] Jansen R. K. *et al.* Analysis of 81 genes from 64 plastid genomes resolves relationships in angiosperms and identifies genome-scale evolutionary patterns. Proc. Natl Acad. Sci. USA 104, 19369–19374 (2007). 18048330 10.1073/pnas.0709121104 PMC2148296

[b19] Davis C. C., Xi Z. & Mathews S. Plastid phylogenomics and green plant phylogeny: almost full circle but not quite there. BMC Biol. 12, 11 (2014). 24533863 10.1186/1741-7007-12-11 PMC3925952

[b20] Smith S. A., Beaulieu J. M. & Donoghue M. J. An uncorrelated relaxed-clock analysis suggests an earlier origin for flowering plants. Proc. Natl Acad. Sci. USA 107, 5897–5902 (2010). 20304790 10.1073/pnas.1001225107 PMC2851901

[b21] Bell C. D., Soltis D. E. & Soltis P. S. The age and diversification of the angiosperms re-revisited. Am. J. Bot. 97, 1296–1303 (2010). 21616882 10.3732/ajb.0900346

[b22] Schneider H. *et al.* Ferns diversified in the shadow of angiosperms. Nature 428, 553–557 (2004). 15058303 10.1038/nature02361

[b23] Moreau C. S., Bell C. D., Vila R., Archibald S. B. & Pierce N. E. Phylogeny of the ants: diversification in the age of angiosperms. Science 312, 101–104 (2006). 16601190 10.1126/science.1124891

[b24] Barrett P. M. & Willis K. J. Did dinosaurs invent flowers? Dinosaur-angiosperm coevolution revisited. Biol. Rev. Camb. Philos. Soc. 76, 411–447 (2001). 11569792 10.1017/s1464793101005735

[b25] Mathews S. & Donoghue M. J. The root of angiosperm phylogeny inferred from duplicate phytochrome genes. Science 286, 947–950 (1999). 10542147 10.1126/science.286.5441.947

[b26] Zimmer E. A. & Wen J. Using nuclear gene data for plant phylogenetics: progress and prospects. Mol. Phylogen. Evol. 66, 539–550 (2013). 10.1016/j.ympev.2013.01.005 23375140

[b27] Duarte J. M. *et al.* Identification of shared single copy nuclear genes in *Arabidopsis*, *Populus*, *Vitis* and *Oryza* and their phylogenetic utility across various taxonomic levels. BMC Evol. Biol. 10, 61 (2010). 20181251 10.1186/1471-2148-10-61 PMC2848037

[b28] Smith S. A. *et al.* Resolving the evolutionary relationships of molluscs with phylogenomic tools. Nature 480, 364–367 (2011). 22031330 10.1038/nature10526

[b29] Rokas A., Williams B. L., King N. & Carroll S. B. Genome-scale approaches to resolving incongruence in molecular phylogenies. Nature 425, 798–804 (2003). 14574403 10.1038/nature02053

[b30] Kocot K. M. *et al.* Phylogenomics reveals deep molluscan relationships. Nature 477, 452–456 (2011). 21892190 10.1038/nature10382 PMC4024475

[b31] Bergsten J. A review of long-branch attraction. Cladistics 21, 163–193 (2005). 10.1111/j.1096-0031.2005.00059.x 34892859

[b32] Jiao Y. *et al.* Ancestral polyploidy in seed plants and angiosperms. Nature 473, 97–100 (2011). 21478875 10.1038/nature09916

[b33] Soltis D. E. *et al.* Polyploidy and angiosperm diversification. Am. J. Bot. 96, 336–348 (2009). 21628192 10.3732/ajb.0800079

[b34] Philippe H. *et al.* Phylogenomics of eukaryotes: impact of missing data on large alignments. Mol. Biol. Evol. 21, 1740–1752 (2004). 15175415 10.1093/molbev/msh182

[b35] Ebersberger I., Strauss S. & Von Haeseler A. HaMStR: Profile hidden markov model based search for orthologs in ESTs. BMC Evol. Biol. 9, 157 (2009). 19586527 10.1186/1471-2148-9-157 PMC2723089

[b36] Stamatakis A. RAxML-VI-HPC: maximum likelihood-based phylogenetic analyses with thousands of taxa and mixed models. Bioinformatics 22, 2688–2690 (2006). 16928733 10.1093/bioinformatics/btl446

[b37] Ronquist F. & Huelsenbeck J. P. MrBayes 3: Bayesian phylogenetic inference under mixed models. Bioinformatics 19, 1572–1574 (2003). 12912839 10.1093/bioinformatics/btg180

[b38] Finet C., Timme R. E., Delwiche C. F. & Marlétaz F. Multigene phylogeny of the green lineage reveals the origin and diversification of land plants. Curr. Biol. 20, 2217–2222 (2010). 21145743 10.1016/j.cub.2010.11.035

[b39] Chase M. W. *et al.* Multigene analyses of monocot relationships: a summary. Aliso 22, 63–75 (2006).

[b40] Qiu Y. *et al.* Angiosperm phylogeny inferred from sequences of four mitochondrial genes. J. Syst. Evol. 48, 391–425 (2010).

[b41] Lee E. K. *et al.* A functional phylogenomic view of the seed plants. PLoS Genet. 7, e1002411 (2011). 22194700 10.1371/journal.pgen.1002411 PMC3240601

[b42] Shimodaira H. & Hasegawa M. CONSEL: for assessing the confidence of phylogenetic tree selection. Bioinformatics 17, 1246–1247 (2001). 11751242 10.1093/bioinformatics/17.12.1246

[b43] Endress P. K. & Doyle J. A. Reconstructing the ancestral angiosperm flower and its initial specializations. Am. J. Bot. 96, 22–66 (2009). 21628175 10.3732/ajb.0800047

[b44] Philippe H. *et al.* Resolving difficult phylogenetic questions: why more sequences are not enough. PLoS Biol. 9, e1000602 (2011). 21423652 10.1371/journal.pbio.1000602 PMC3057953

[b45] Delsuc F., Brinkmann H. & Philippe H. Phylogenomics and the reconstruction of the tree of life. Nat. Rev. Genet. 6, 361–375 (2005). 15861208 10.1038/nrg1603

[b46] Struck T. H. TreSpEx—detection of misleading signal in phylogenetic reconstructions based on tree information. Evol. Bioinform. Online 10, 51 (2014). 24701118 10.4137/EBO.S14239 PMC3972080

[b47] Fourment M. & Gibbs M. J. PATRISTIC: a program for calculating patristic distances and graphically comparing the components of genetic change. BMC Evol. Biol. 6, 1 (2006). 16388682 10.1186/1471-2148-6-1 PMC1352388

[b48] Dávalos L. M. & Perkins S. L. Saturation and base composition bias explain phylogenomic conflict in *Plasmodium*. Genomics 91, 433–442 (2008). 18313259 10.1016/j.ygeno.2008.01.006

[b49] Sanderson M. J. r8s: inferring absolute rates of molecular evolution and divergence times in the absence of a molecular clock. Bioinformatics 19, 301–302 (2003). 12538260 10.1093/bioinformatics/19.2.301

[b50] Drummond A. J. & Rambaut A. BEAST: Bayesian evolutionary analysis by sampling trees. BMC Evol. Biol. 7, 214 (2007). 17996036 10.1186/1471-2148-7-214 PMC2247476

[b51] Magallón S. Using fossils to break long branches in molecular dating: a comparison of relaxed clocks applied to the origin of angiosperms. Syst. Biol. 59, 384–399 (2010). 20538759 10.1093/sysbio/syq027

[b52] Friis E. M., Crane P. R. & Pedersen K. R. Early Flowers and Angiosperm Evolution Cambridge University Press (2011).

[b53] Hochuli P. A. & Feist-Burkhardt S. Angiosperm-like pollen and *Afropollis* from the Middle Triassic (Anisian) of the Germanic Basin (Northern Switzerland). Front. Plant. Sci. 4, 344 (2013). 24106492 10.3389/fpls.2013.00344 PMC3788615

[b54] Labandeira C. C. & Eble G. J. inGondwana Alive: Biodiversity and the Evolving Terrestrial Biosphere eds John A., de Wit M., Thackeray F. Witwatersrand University Press (2000).

[b55] Rokas A. & Carroll S. B. Bushes in the tree of life. PLoS Biol. 4, e352 (2006). 17105342 10.1371/journal.pbio.0040352 PMC1637082

[b56] Grabherr M. G. *et al.* Full-length transcriptome assembly from RNA-Seq data without a reference genome. Nat. Biotechnol. 29, 644–652 (2011). 21572440 10.1038/nbt.1883 PMC3571712

[b57] Pertea G. *et al.* TIGR Gene Indices clustering tools (TGICL): a software system for fast clustering of large EST datasets. Bioinformatics 19, 651–652 (2003). 12651724 10.1093/bioinformatics/btg034

[b58] Koonin E. V. Orthologs, paralogs, and evolutionary genomics. Annu. Rev. Genet. 39, 309–338 (2005). 16285863 10.1146/annurev.genet.39.073003.114725

[b59] Abascal F., Zardoya R. & Posada D. ProtTest: selection of best-fit models of protein evolution. Bioinformatics 21, 2104–2105 (2005). 15647292 10.1093/bioinformatics/bti263

[b60] Edgar R. C. MUSCLE: multiple sequence alignment with high accuracy and high throughput. Nucleic Acids Res. 32, 1792–1797 (2004). 15034147 10.1093/nar/gkh340 PMC390337

[b61] Capella-Gutiérrez S., Silla-Martínez J. M. & Gabaldón T. trimAl: a tool for automated alignment trimming in large-scale phylogenetic analyses. Bioinformatics 25, 1972–1973 (2009). 19505945 10.1093/bioinformatics/btp348 PMC2712344

[b62] Gouy M., Guindon S. & Gascuel O. SeaView version 4: a multiplatform graphical user interface for sequence alignment and phylogenetic tree building. Mol. Biol. Evol. 27, 221–224 (2010). 19854763 10.1093/molbev/msp259

[b63] Nylander J. A., Wilgenbusch J. C., Warren D. L. & Swofford D. L. AWTY (are we there yet?): a system for graphical exploration of MCMC convergence in Bayesian phylogenetics. Bioinformatics 24, 581–583 (2008). 17766271 10.1093/bioinformatics/btm388

[b64] Stefanović S., Rice D. W. & Palmer J. D. Long branch attraction, taxon sampling, and the earliest angiosperms: *Amborella* or monocots? BMC Evol. Biol. 4, 35 (2004). 15453916 10.1186/1471-2148-4-35 PMC543456

[b65] Ming R. *et al.* Genome of the long-living sacred lotus (*Nelumbo nucifera* Gaertn.). Genome Biol. 14, R41 (2013). 23663246 10.1186/gb-2013-14-5-r41 PMC4053705

[b66] Swofford D. L. PAUP*. Phylogenetic Analysis Using Parsimony (* and Other Methods). Version 4 Sinauer Associates (2003).

[b67] Maddison W. P. & Maddison D. R. Mesquite: a modular system for evolutionary analysis. Version 2.75, http://mesquiteproject.org (2011).

[b68] Gao Z. & Barry A. T. A review of fossil cycad megasporophylls, with new evidence of *Crossozamia* pomel and its associated leaves from the lower permian of Taiyuan, China. Rev. Palaeobot. Palynol. 60, 205–223 (1989).

[b69] Mapes G. & Rothwell G. W. Structure and relationships of primitive conifers. Neues Jahrb. Geol. Palaontol. Abh. 183, 269–287 (1991).

[b70] Hughes N. F. The Enigma of Angiosperm Origins Cambridge University Press (1994).

