# Supplementary material for: Resolution of deep angiosperm phylogeny using conserved nuclear genes and estimates of early divergence times
Source: Nat Commun. 2014 Sep 24;5:4956. doi: 10.1038/ncomms5956 (PMC4200517; doi:10.1038/ncomms5956)
Supplement: Supplementary Figures and Tables — Supplementary Figures 1-22 and Supplementary Tables 1-6 [file ncomms5956-s1.pdf]

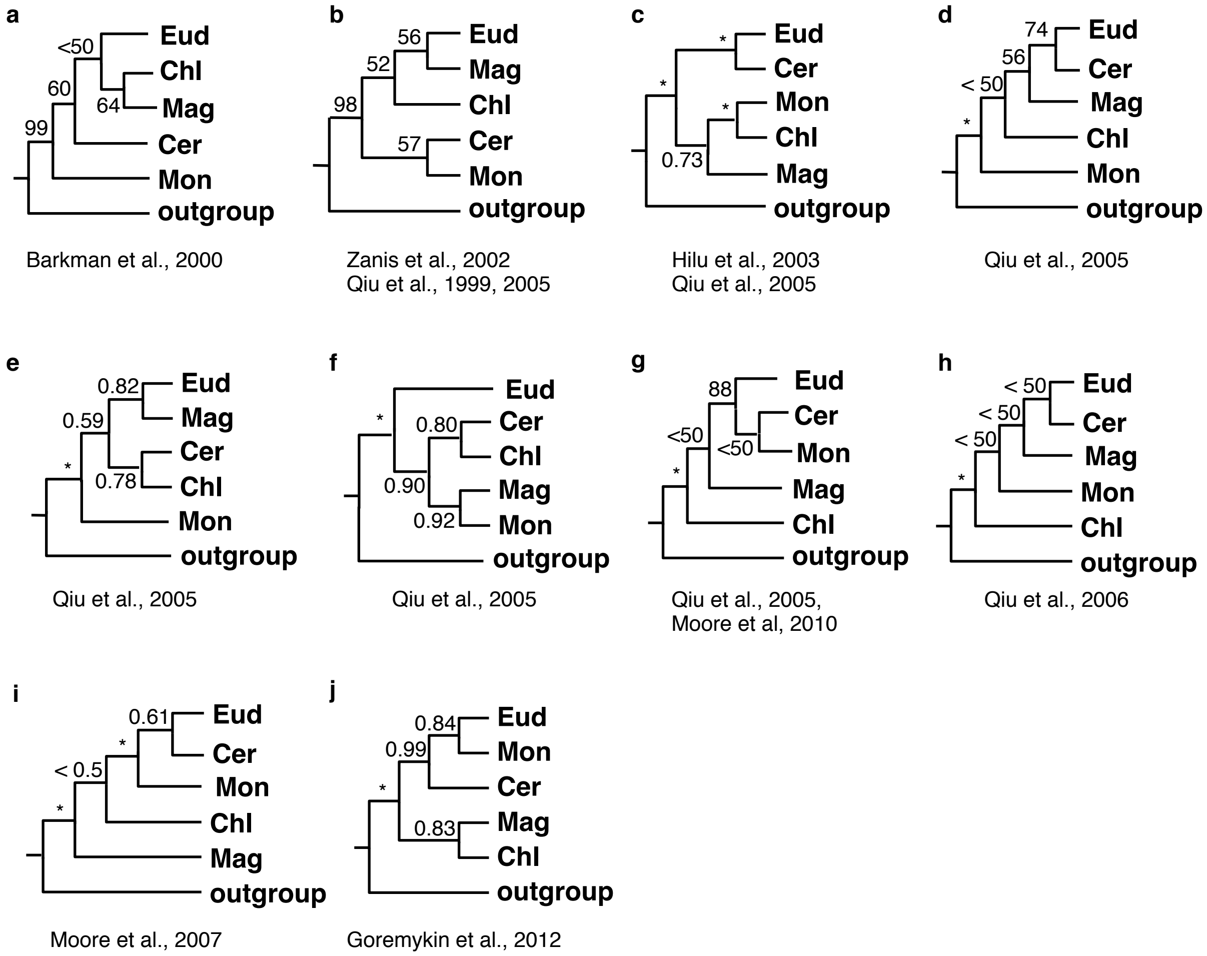

Supplementary Fig. 1. Ten additional alternative topologies among eudicots (Eud), monocots (Mon), magnoliids (Mag), Ceratophyllaceae (Cer) and Chloranthaceae (Chl) modified from previous studies. An asterisk indicates that the bootstrap value or posterior probability reached 100% or 1.0, respectively. Other bootstrap values (0<BS<100) or posterior probabilities (0<PP<1.0) are indicated.

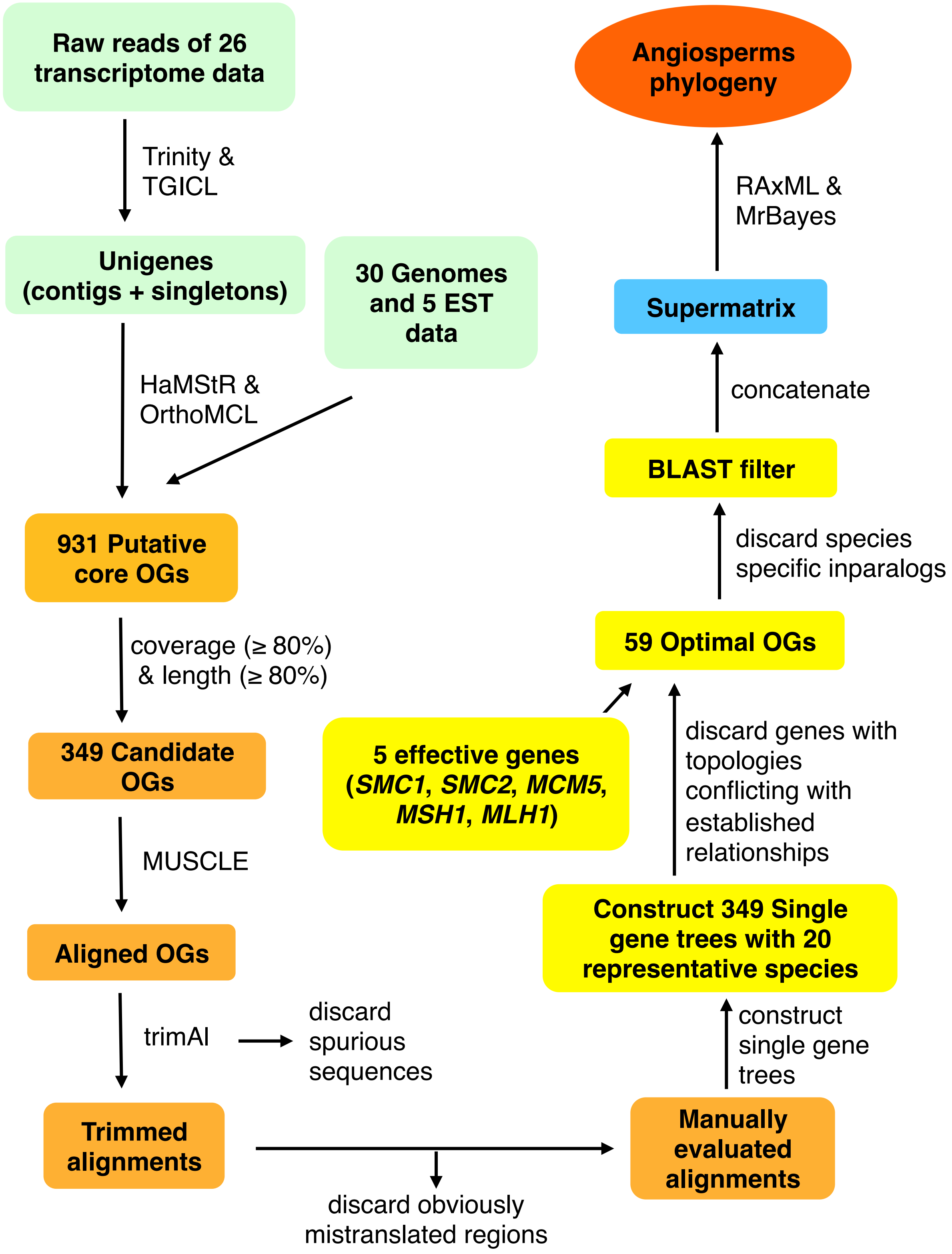

Supplementary Fig. 2. Flowchart of data analysis.

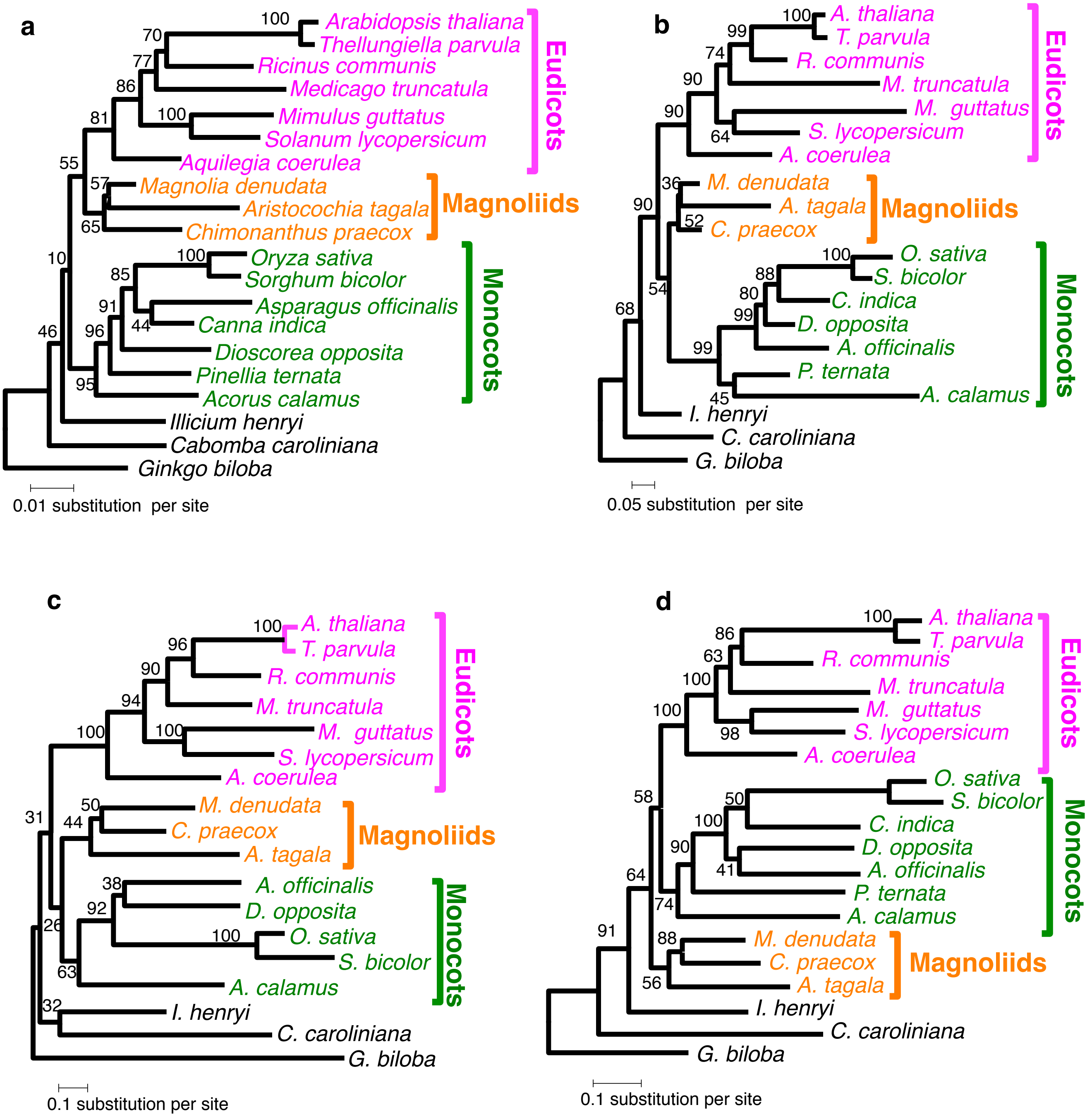

Supplementary Fig. 3 (a-d). Examples of single gene trees of selected marker genes in accordance with species trees of 20 representative species with established relationships. Taxa in different major groups of mesangiosperms are shown in different colors as indicated. Numbers in each nodes are ML bootstrap values obtained by RAxML.

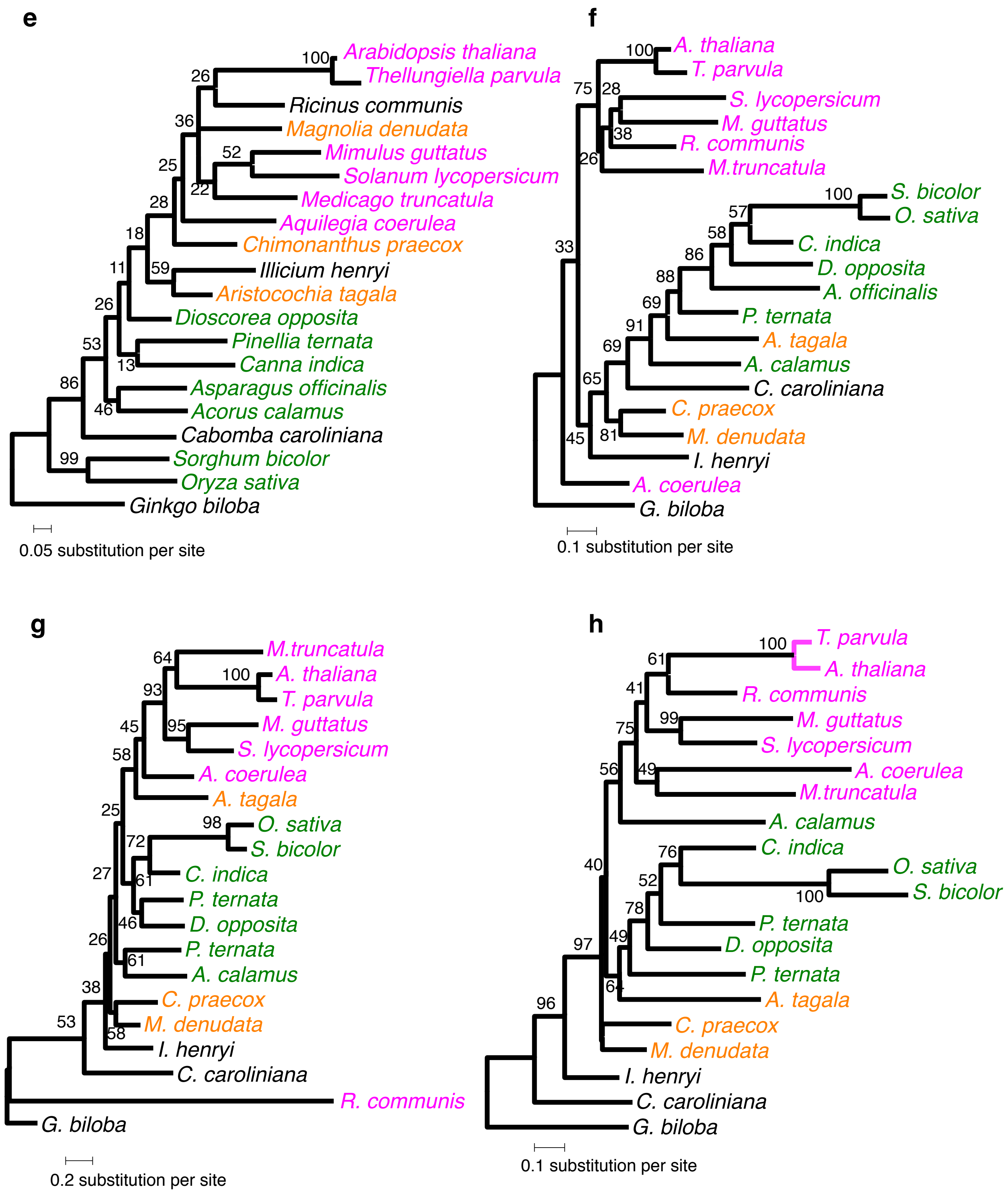

Supplementary Fig. 3 (e-h). Examples of single gene trees for excluded candidate genes conflicting with species trees of 20 representative species with established relationships. Numbers in each nodes are ML bootstrap obtained by RAxML. Species in purple, green and red are from eudicots, monocots and magnoliids, respectively.

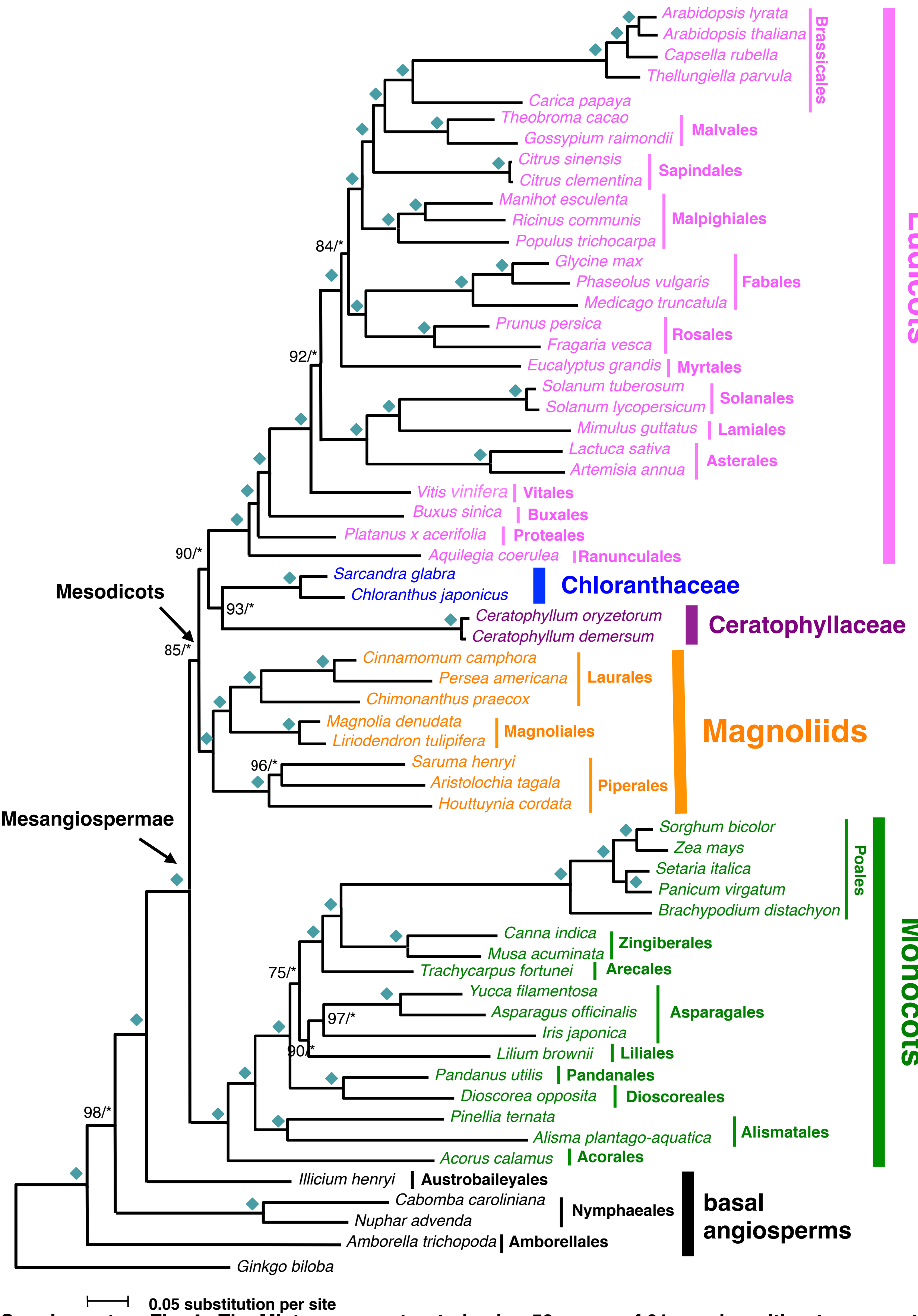

Supplementary Fig. 4. The ML tree reconstructed using 59 genes of 61 species without gene partition. Numbers associated with nodes are the bootstrap value (BS, on the left) obtained by RAxML and the posterior probability (PP, on the right) obtained by MrBayes based on concatenated 59 genes. Asterisks indicate BS of 100% or PP of 1.0. Diamonds indicate BS of 100% and PP of 1.0.

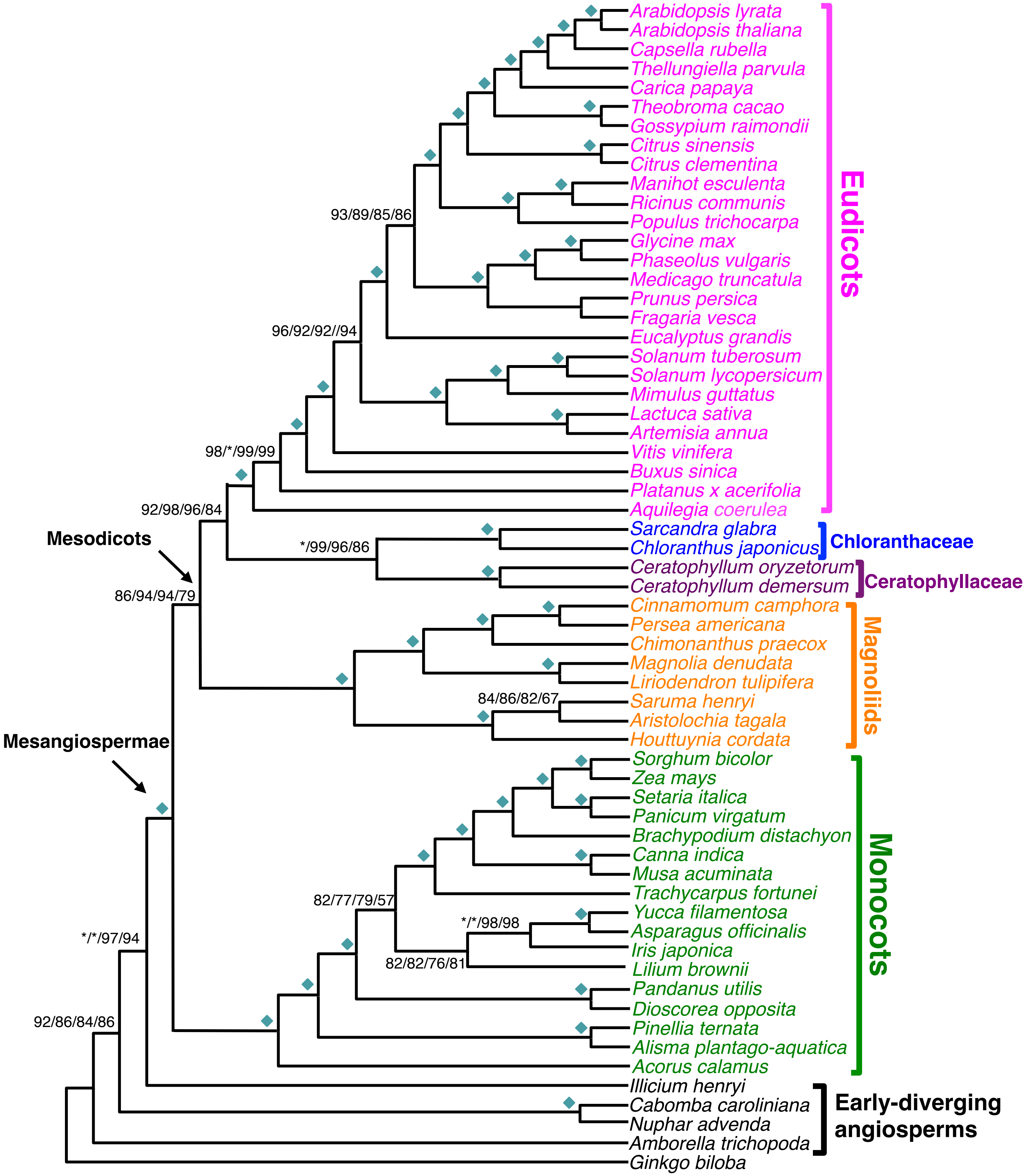

Supplementary Fig. 5. Trees based on different evolutionary models. This cladogram was inferred by RAxML based on concatenated 59-gene amino acid sequences. Taxa in different major groups of mesangiosperms are shown in different colors as indicated. Numbers associated with branches are bootstrap values (BS) based on JTT + CAT, JTT + G, WAG + CAT, and DAYHOFF + CAT from left to right; BS of 100% is indicated by asterisk; a diamond indicates that all four BS values are 100%.

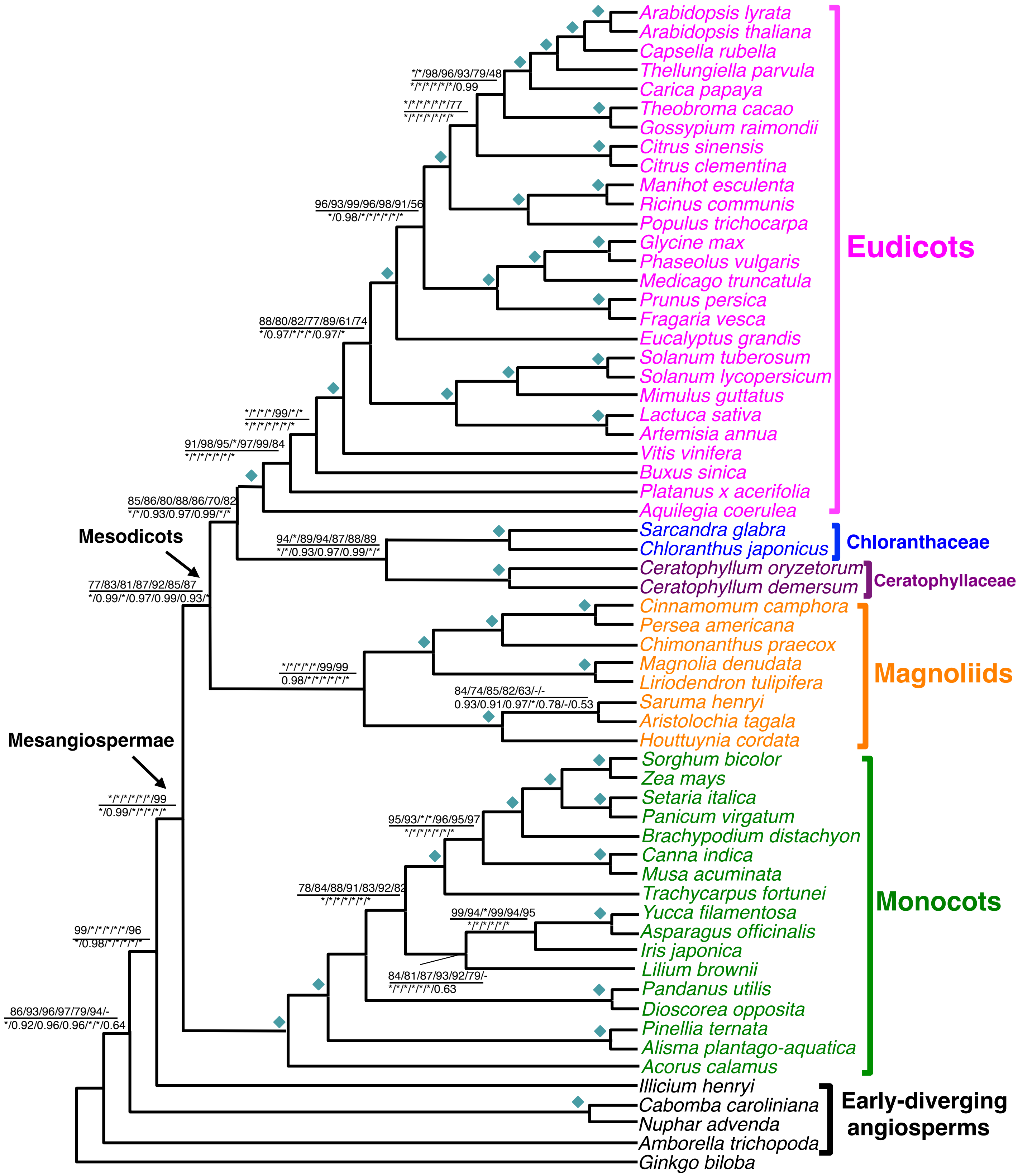

Supplementary Fig. 6. Trees based on different numbers of genes. A cladogram inferred using RAxML based on concatenated 59-gene amino acid sequences. Numbers associated with branches are bootstrap values (above) obtained by RAxML and posterior probability (below) obtained by MrBayes based on 55, 50, 46, 41, 33, 25 and 16 genes from left to right, respectively. A diamond indicates all seven bootstrap values and posterior possibilities on this node reaching 100% and 1.0. A dash indicates different topology at this node when compared with the 59-gene tree.

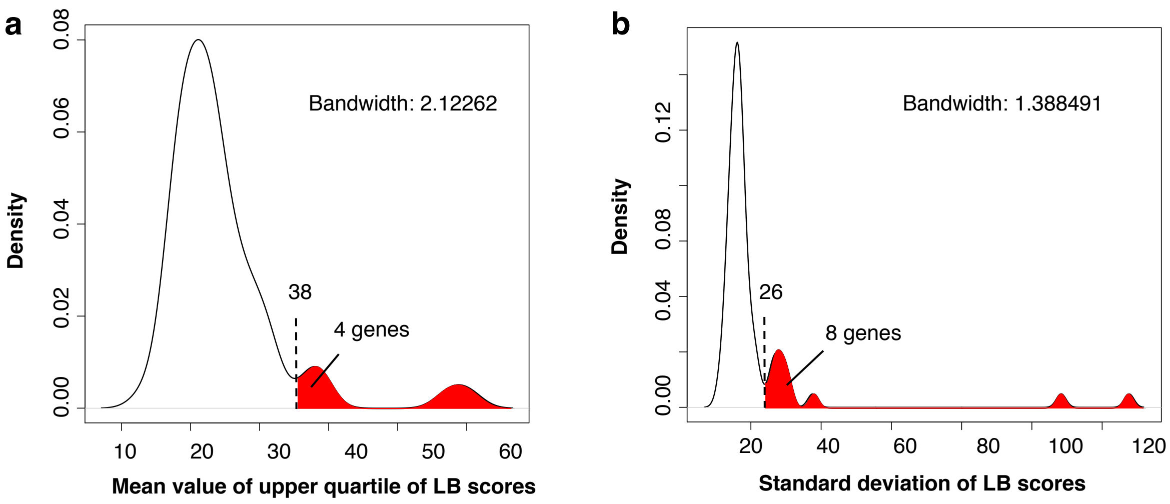

Supplementary Fig. 7. Density plots generated with R of different gene-specific long-branch indices for the 59 genes. (**a**) Average of the upper quartile of LB scores representing the genes with the longest branches. (**b**) Standard deviation of LB scores measuring heterogeneity. Dashed line indicates starting shoulder value. Red areas in the right-hand side indicate deviations from the normal distribution and comprise genes might be long branches.

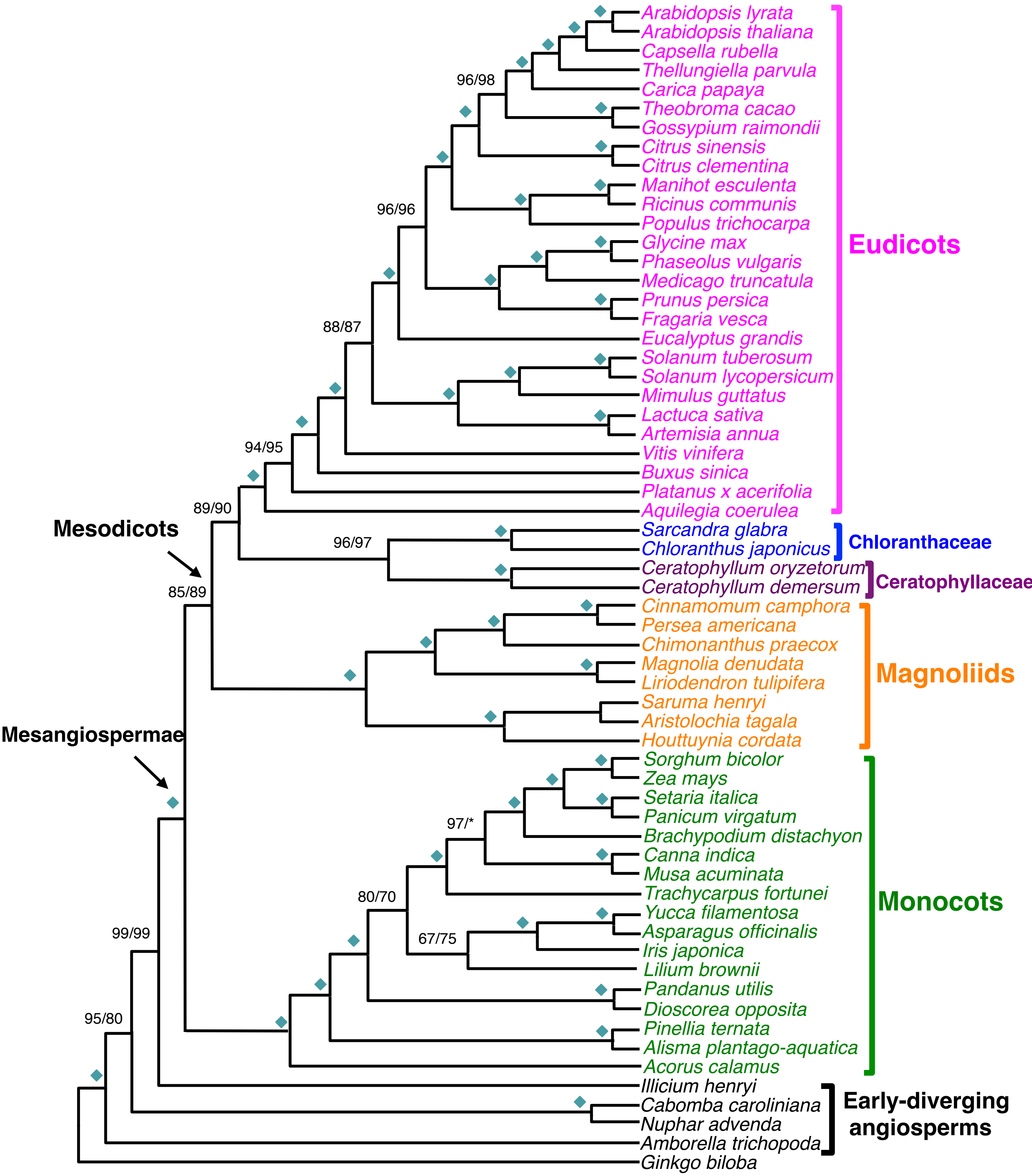

Supplementary Fig. 8. ML Trees inferred by removing genes with high long branch scores. A cladogram inferred using RAxML based on concatenated 55-gene amino acid sequences. Numbers associated with branches are bootstrap (BS) values obtained by RAxML based on 55 and 51 genes, which excluded four (gene ID: 434655, 434734, 434354 and MCM5) and eight (gene ID: 434655, 434734, 434354, MCM5, 432200, 431723, 433581 and 434860) genes according to gene-specific long-branch scores, from left to right, respectively. A diamond indicates both BS values of 100% at this node. An asterisk indicates one of the BS values at this node being 100%. Other BS values are indicated.

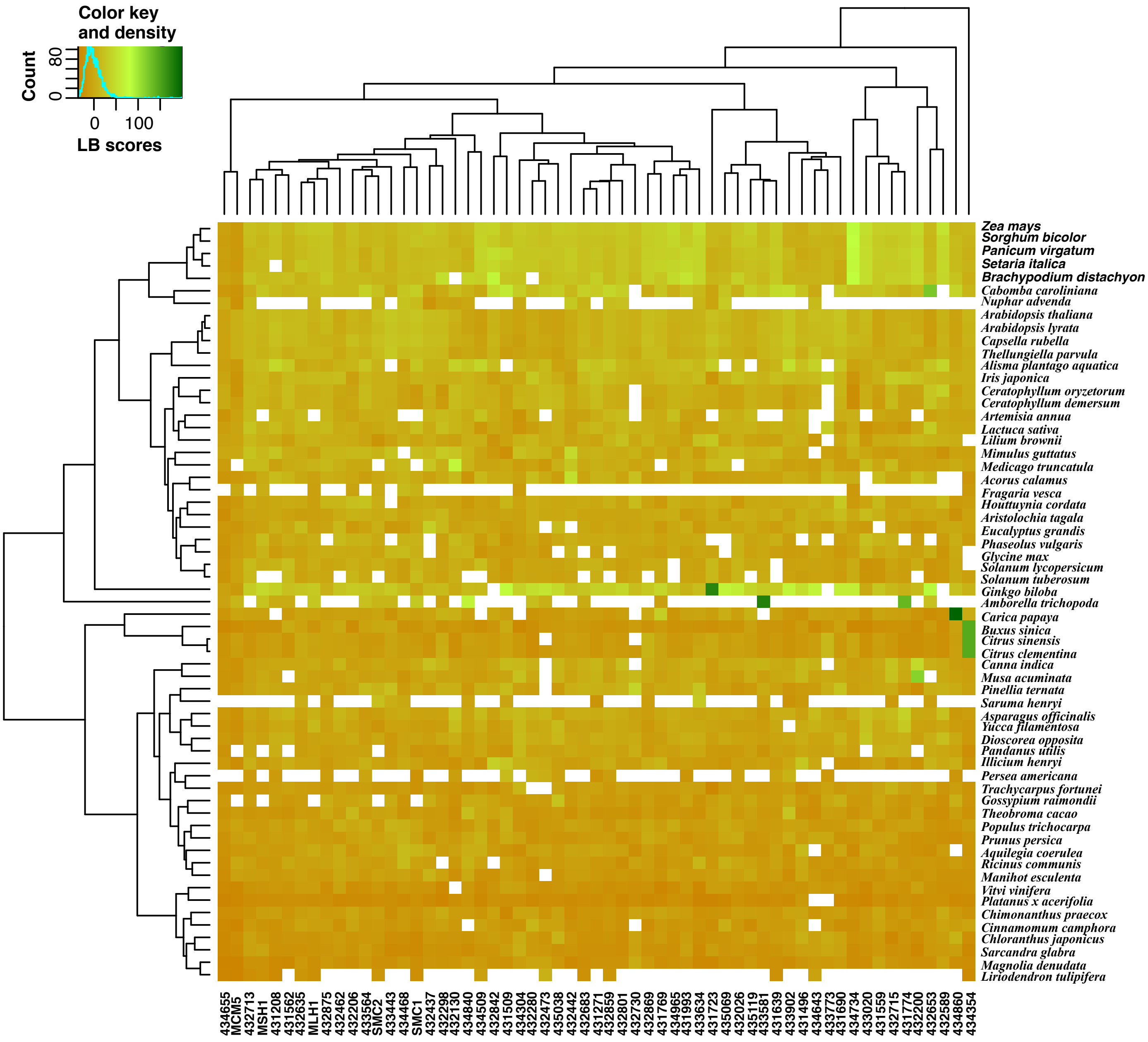

Supplementary Fig. 9. Heat map in combination with hierarchical clustering generated with R of the taxon-specific long-branch scores for 59 genes of 61 species. Row show taxa and columns genes. Color key and density plot of long-branch **(**LB) scores are shown in the upper left corner of the heat map. White cells in the map indicate that the taxon was lacking in that gene. Orange cells indicate that the taxon with low LB scores indicate that the taxon in that gene might be not affected by long-branch attraction. Dark green cells (genes with ID of 434680 in *Carica papaya*, 433581 in *Amborella trichopoda* and 431723 in *Ginkgo biloba*) with high LB scores indicate that the taxon in that gene might be a long-branch.

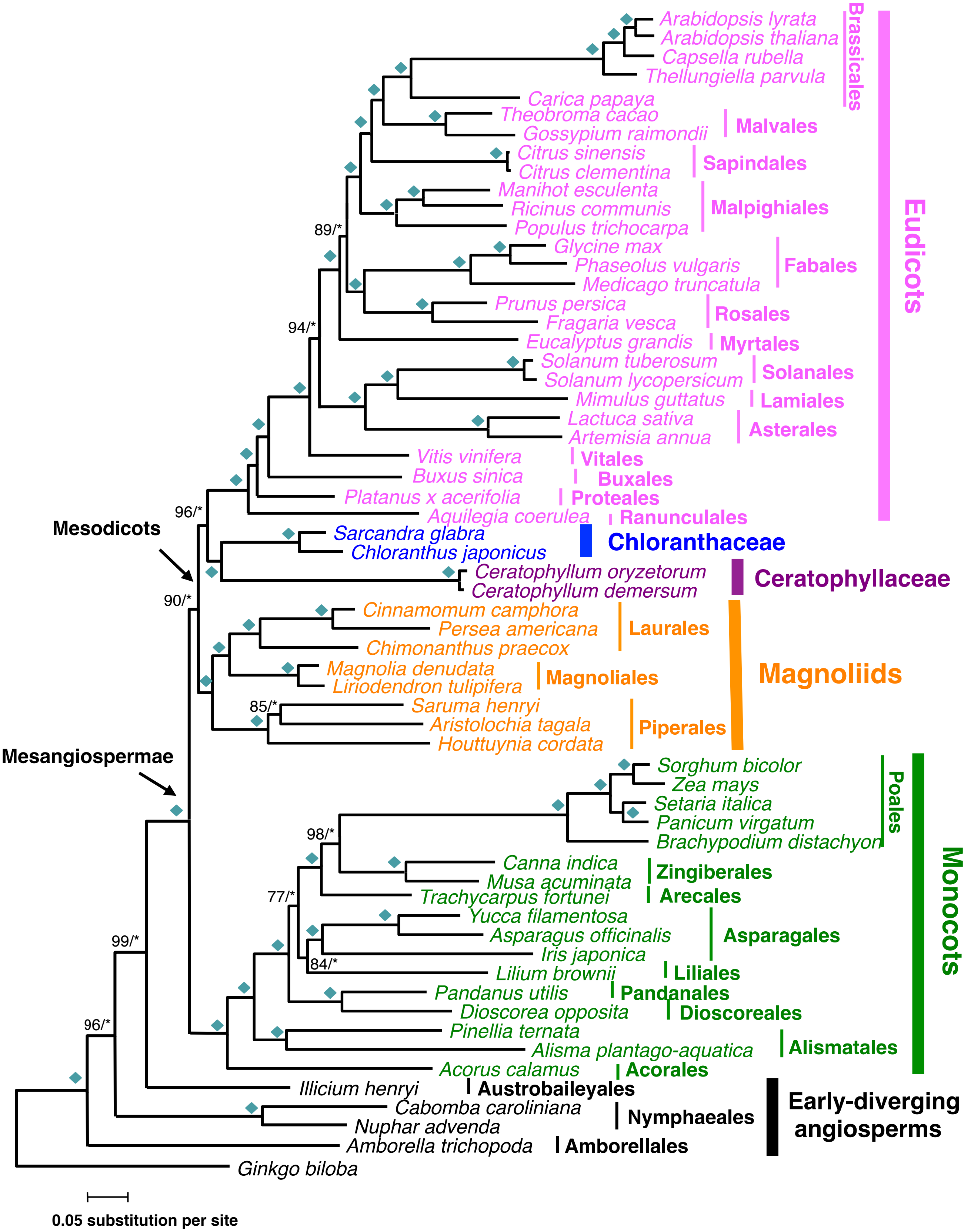

Supplementary Fig. 10. A phylogram of the best ML tree inferred using RAxML based on concatenated 59-gene amino acid sequences from 61 species. Genes with ID of 434680 in *Carica papaya*, 433581 in *Amborella trichopoda* and 431723 in *Ginkgo biloba* with the highest LB scores according to Supplementary Fig 9 were excluded in this analysis. Numbers associated with branches are bootstrap value (BS) and posterior probability (PP) inferred by RAxML and MrBayes, respectively. An asterisk indicates PP of 1.0. A diamond indicates BS of 100% and PP of 1.0 at the same time.

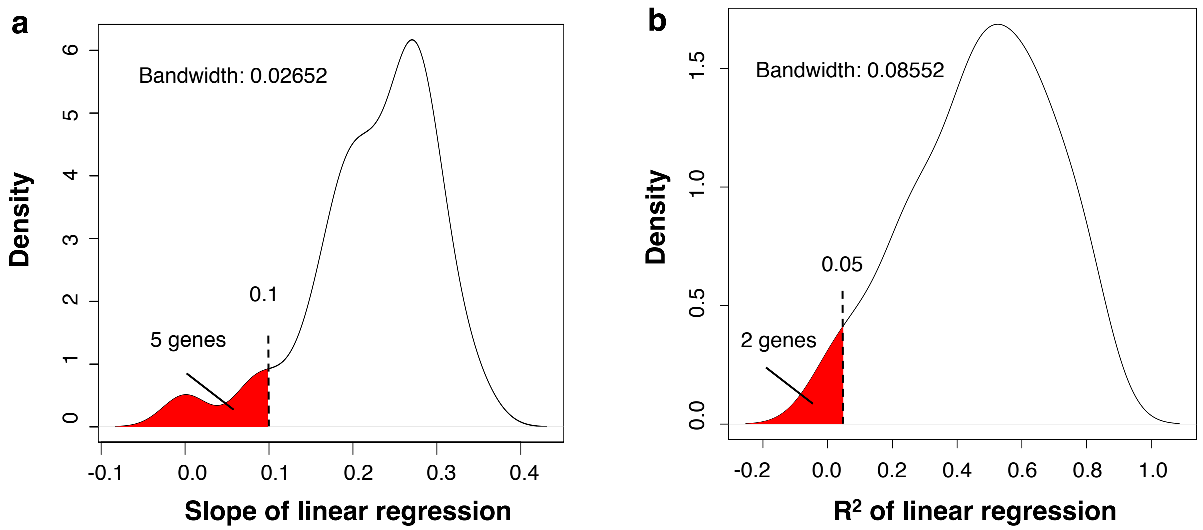

Supplementary Fig. 11 Density plots (distribution) of different gene-specific saturation indices for the 59 genes generated with R . (**A**) Slopes of the linear regression between patristic and uncorrected pairwise distances. (**B**) R^2 of the linear regression between patristic and uncorrected pairwise distances. Dashed line indicates starting shoulder value. Red areas in the left-hand side comprise genes that might be saturated.

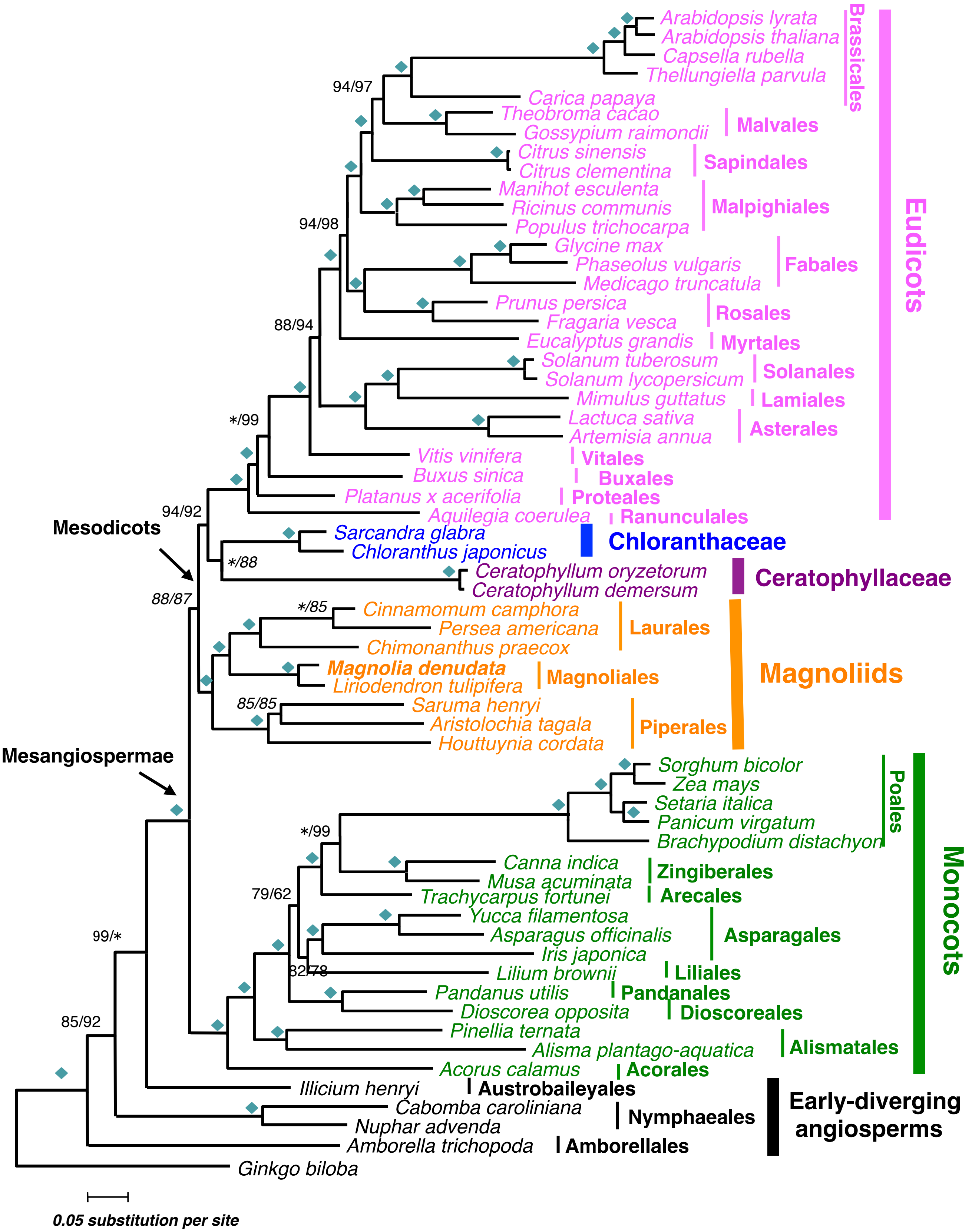

Supplementary Fig. 12. A phylogram of the best ML tree inferred using RAxML based on unsaturated genes amino acid sequences from 61 species. Numbers associated with branches are bootstrap (BS) values obtained by RAxML based on 54 and 57 genes, which excluded five genes (gene ID: 434655, MCM5, 433581, 431774 and MSH1) with Slope ≤ 0.1 and two genes (gene ID: 434655 and MCM5) with R^2 ≤ 0.05 according to Supplementary Fig 11, respectively, from 59-gene dataset. An asterisk indicates BS of 100% and a diamond indicates both two BS on this node of 100%.

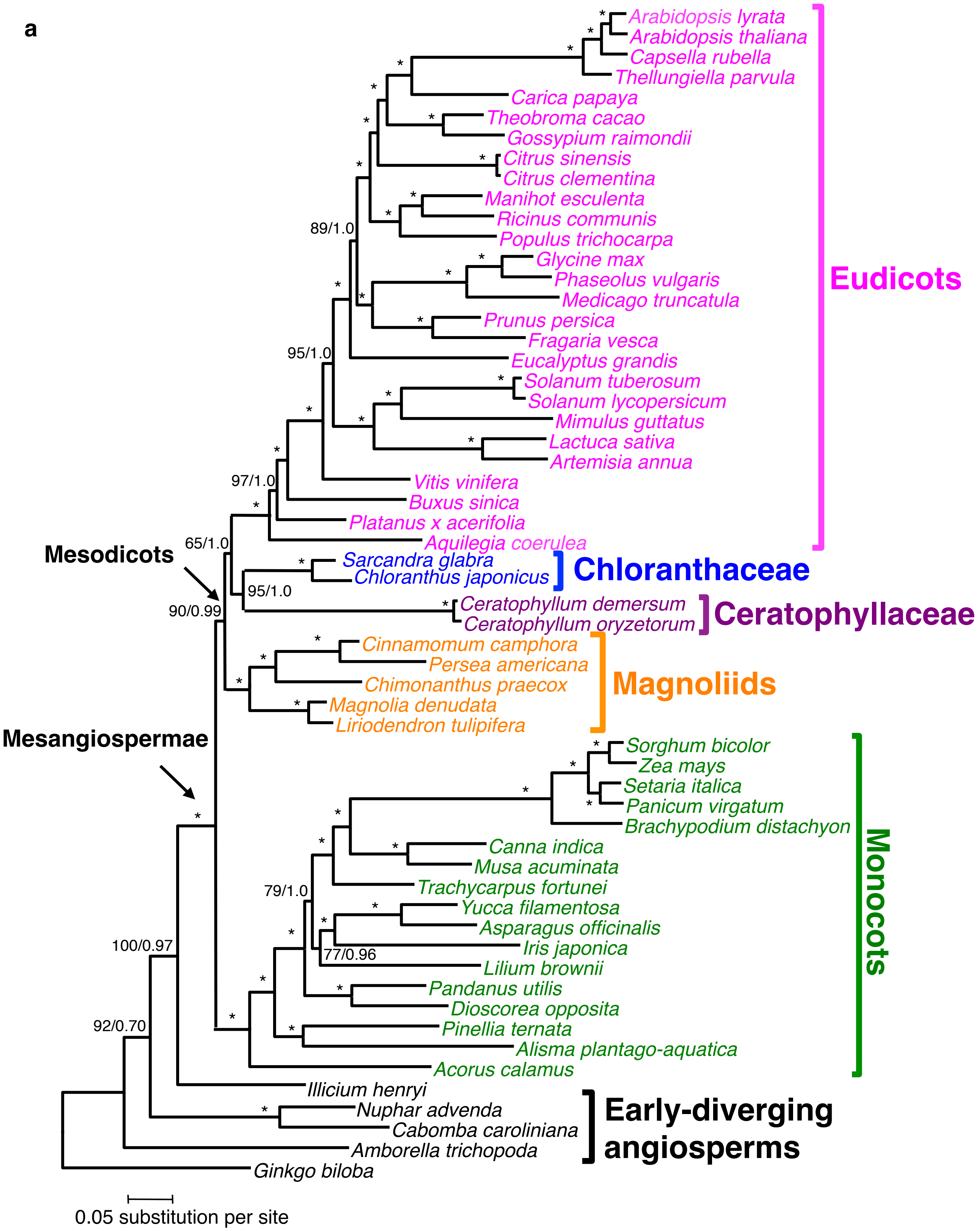

Supplementary Fig. 13a. A phylogram of the best ML tree inferred using RAxML based on concatenated 59-gene amino acid sequences from 58 species. Three species from Piperales of magnoliids were excluded in this analysis; numbers associated with branches are bootstrap values (BS) and posterior probability (PP) inferred by RAxML and MrBayes, respectively; an asterisk indicates BS of 100% and PP of 1.0 at the same time.

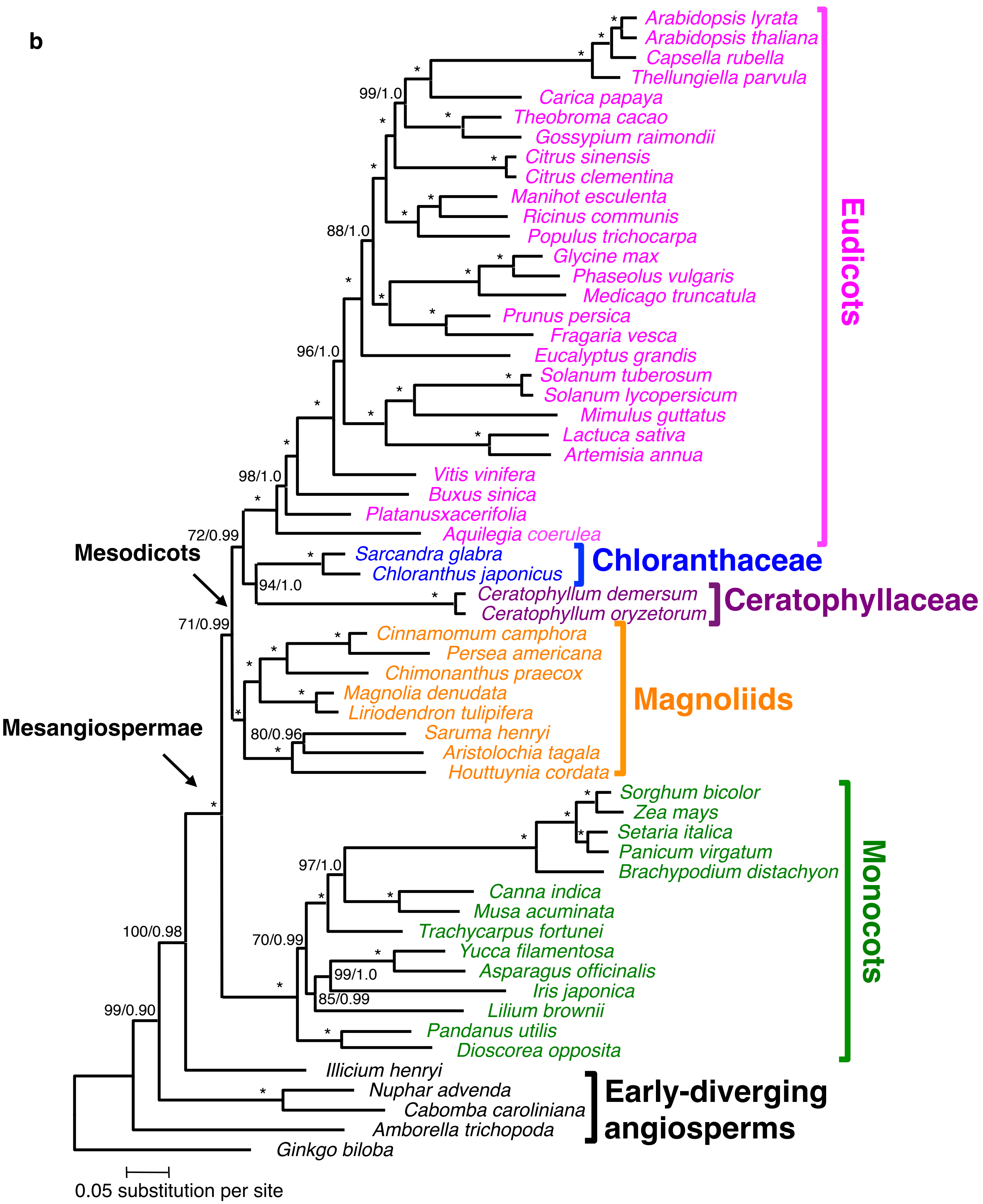

Supplementary Fig. 13b. A phylogram of the best ML tree inferred using RAxML based on concatenated 59-gene amino acid sequences from 58 species . Three species from basal monocots (*Acorus calamus*, *Alisma plantago-aquatica*, and *Pinellia ternata*) were excluded in this analysis; numbers associated with branches are bootstrap values (BS) and posterior probability (PP) inferred by RAxML and MrBayes, respectively; an asterisk indicates BS of 100% and PP of 1.0 at the same time.

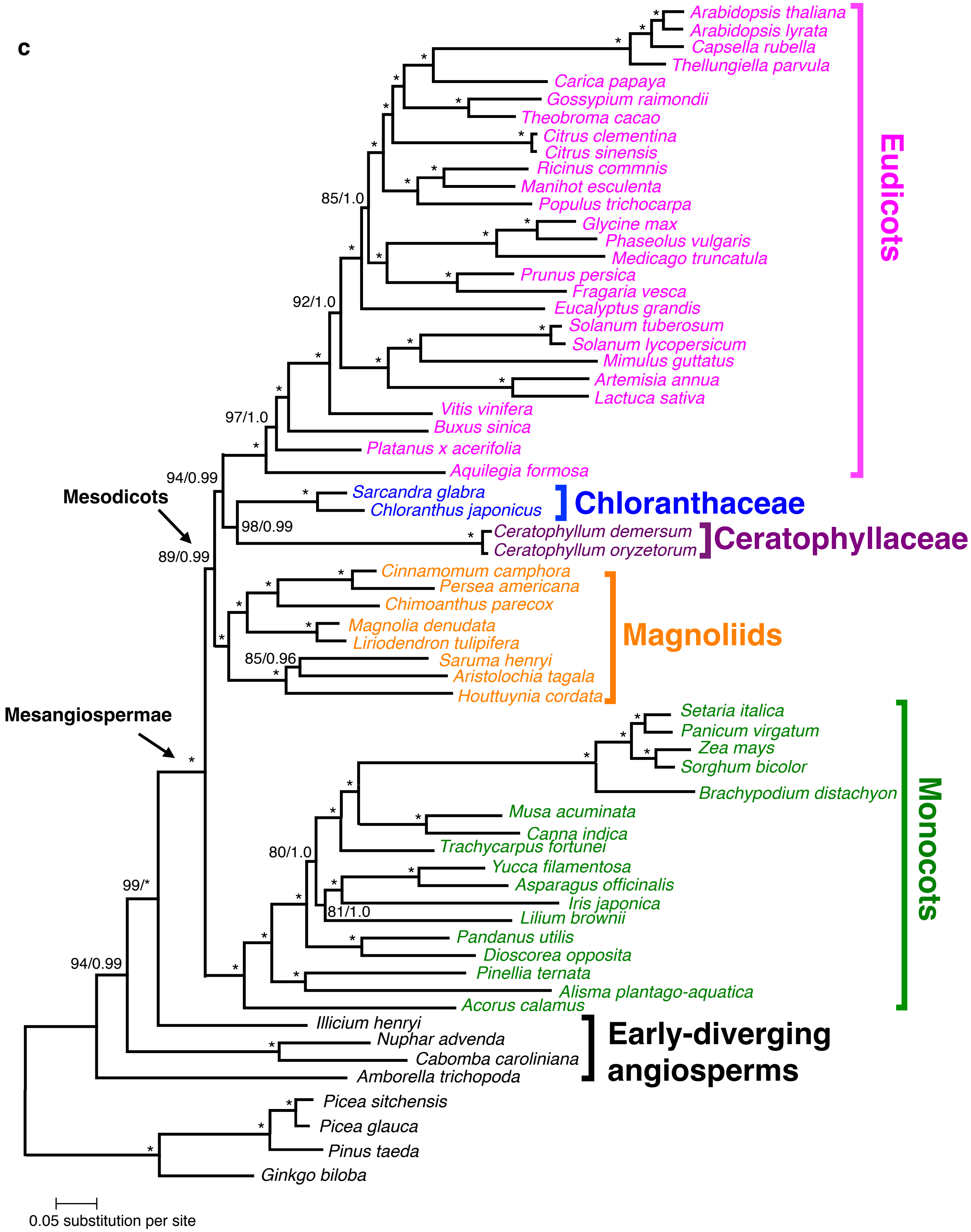

Supplementary Fig. 13c. A phylogram of the best ML tree inferred using RAxML based on concatenated 59-gene amino acid sequences from 64 species. Three Pinaceae species (*Picea sitchensis*, *Picea glauca*, and *Pinus taeda*) were included in this analysis; numbers associated with branches are bootstrap values (BS) and posterior probability (PP) inferred by RAxML and MrBayes, respectively; an asterisk indicates BS of 100% and PP of 1.0 at the same time.

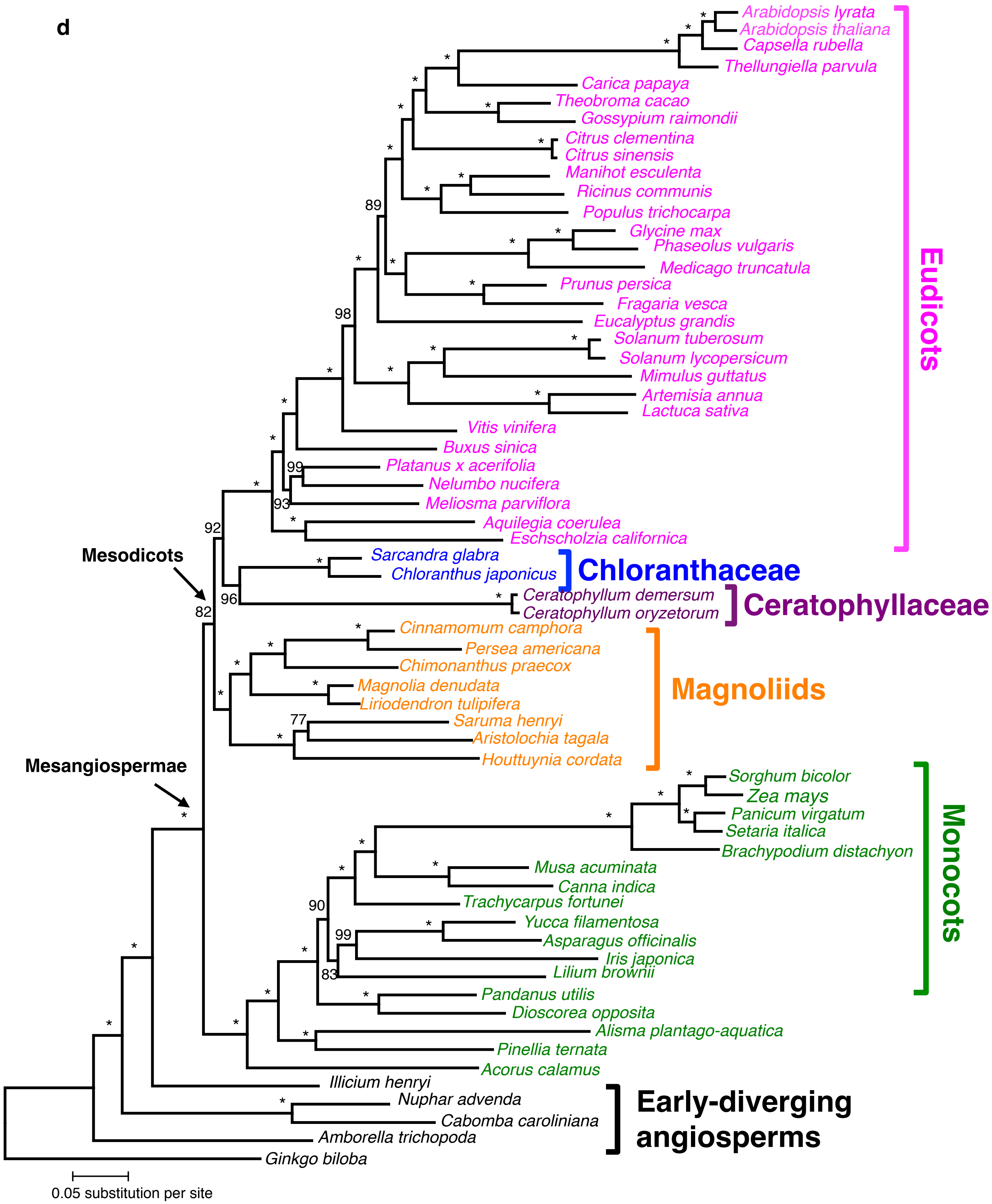

Supplementary Fig. 13d. An ML tree inferred by RAxML based on concatenated 59-gene amino acid sequences from 63 species. Three basal eudicot species (*Nelumbo nucifera*, *Meliosma parviflora* and *Eschscholzia californica*) were included in this analysis; numbers associated with branches are bootstrap values inferred by RAxML; an asterisk indicates BS of 100%.

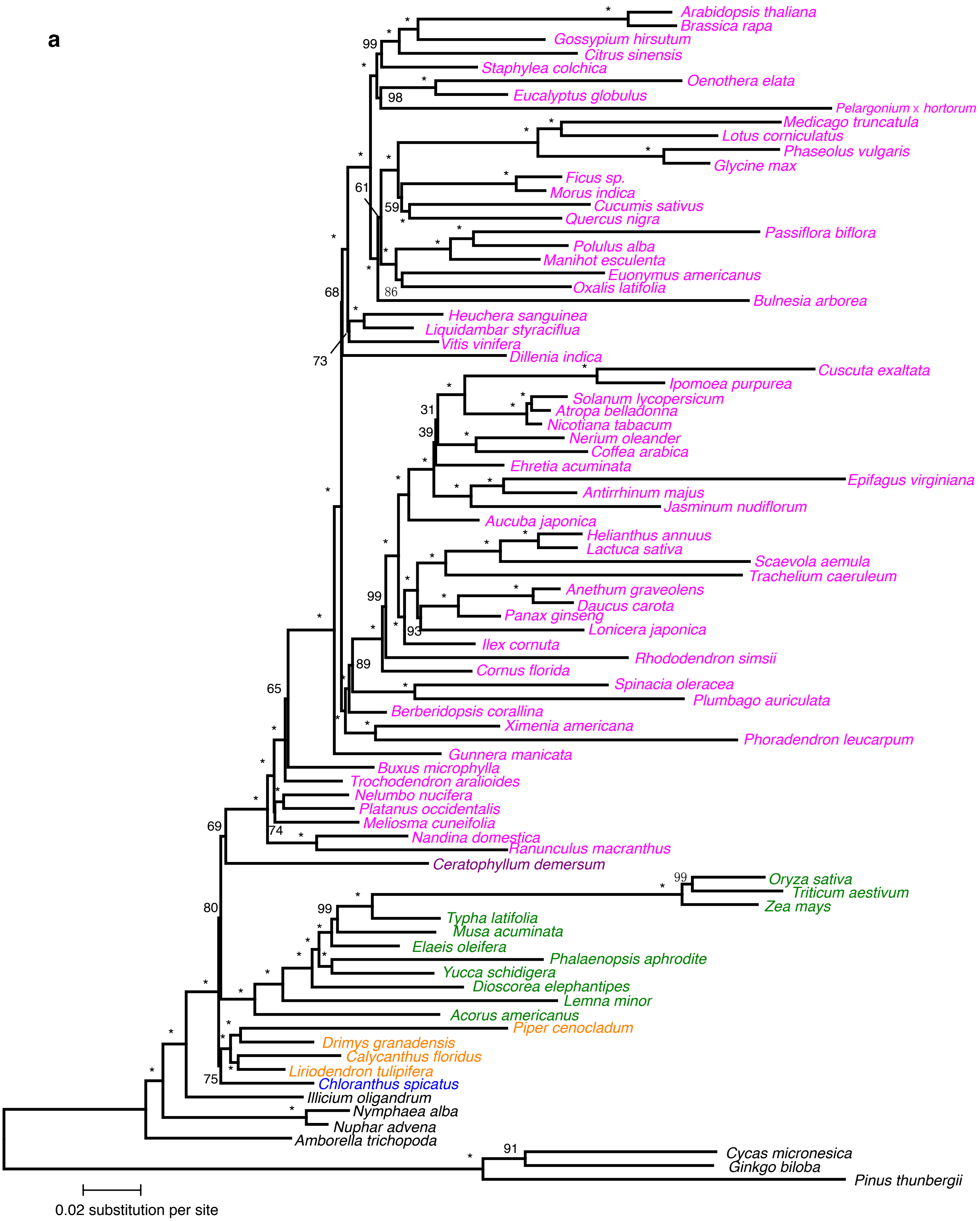

Supplementary Fig. 14a. The ML tree inferred by RAxML based on concatenated 83 plastid genes of 86 species. Numbers associated with branches are bootstrap values; an asterisk indicates bootstrap of 100%. Species from eudicots, monocots, magnoliids, Chloranthaceae and Ceratophyllaceae are in purple, green, orange, violet and blue, respectively.

b

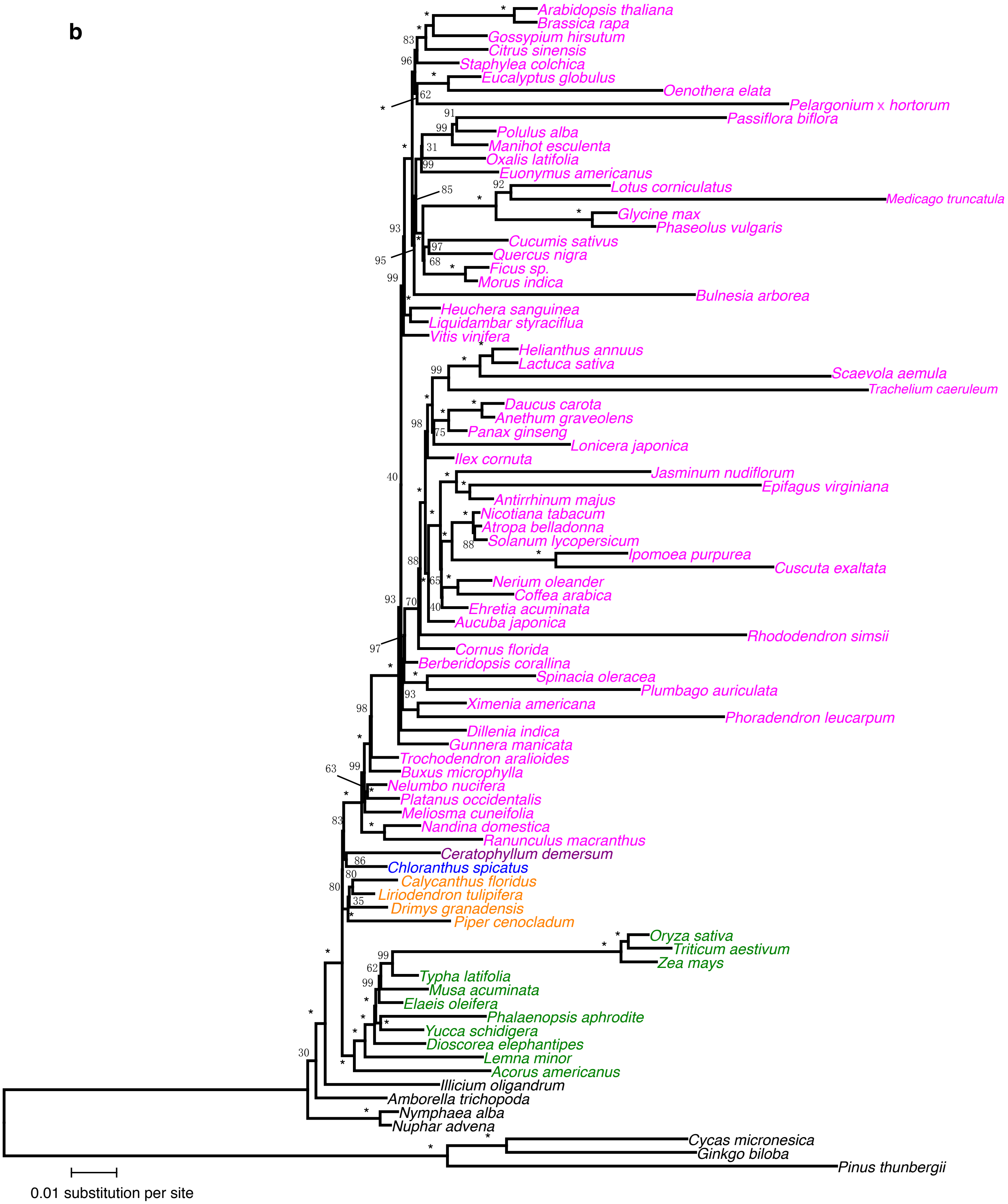

Supplementary Fig. 14b. The ML tree inferred by RAxML based on 40 concatenated plastid inverted repeat genes of 86 species. Numbers associated with branches are bootstrap values; an asterisk indicates bootstrap of 100%. Species from eudicots, monocots, magnoliids, Chloranthaceae and Ceratophyllaceae are in purple, green, orange, violet and blue, respectively.

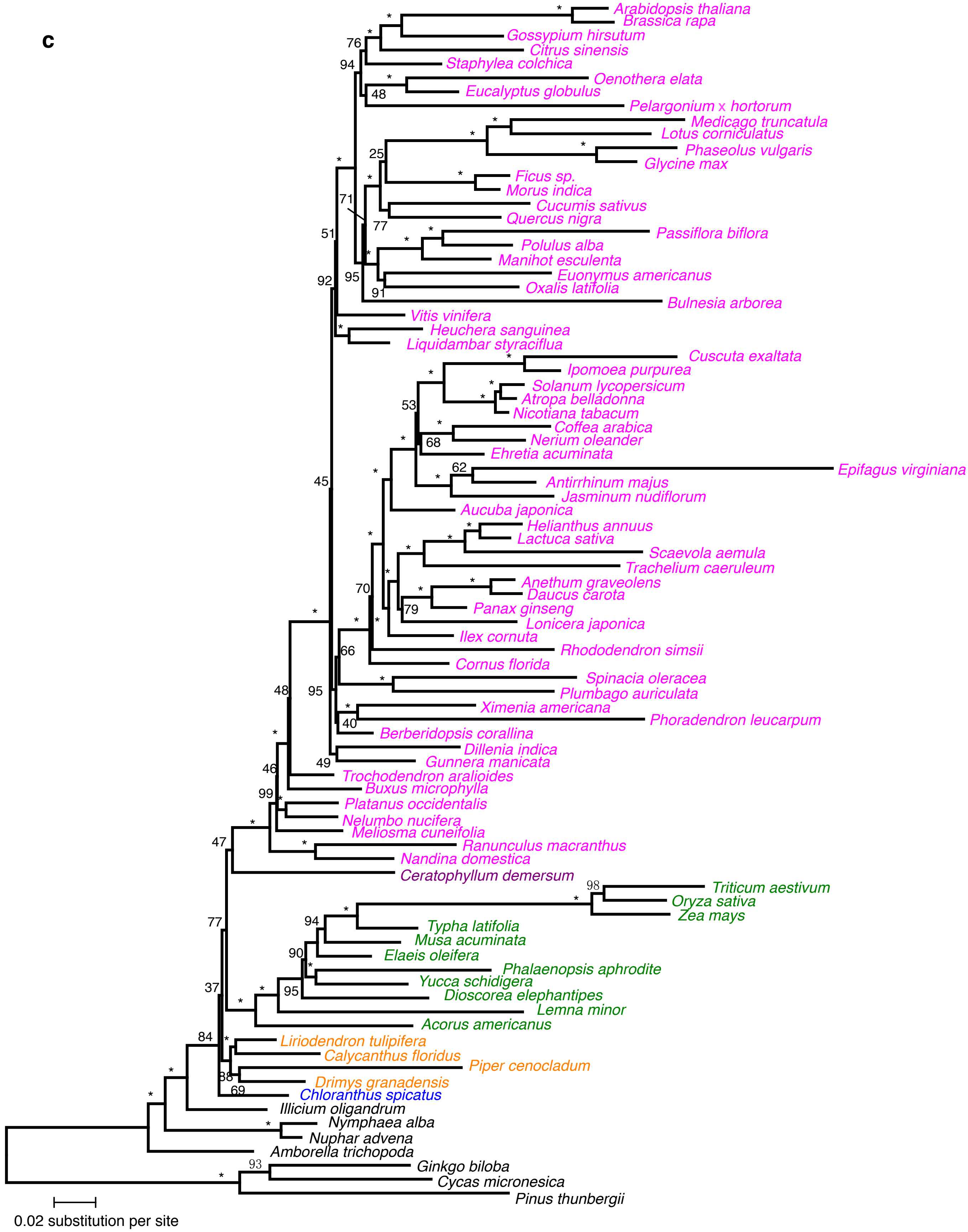

Supplementary Fig. 14c. The ML tree inferred by RAxML based on concatenated 34 plastid genes of 86 species with percentage of parsimony informative sites less than 40%. Numbers associated with branches are bootstrap values; an asterisk indicates bootstrap of 100%. Species from eudicots, monocots, magnoliids, Chloranthaceae and Ceratophyllaceae are in purple, green, orange, violet and blue, respectively.

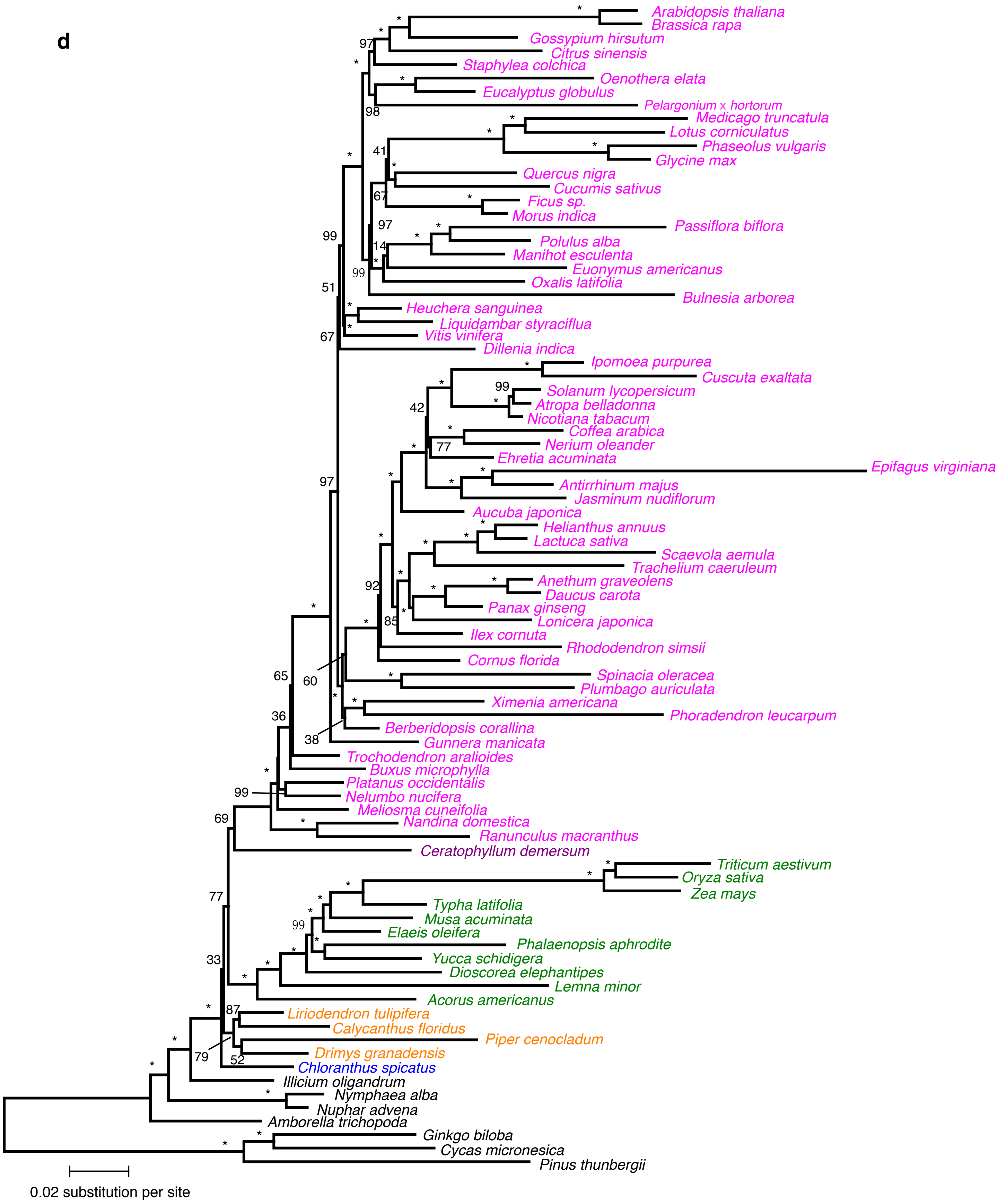

Supplementary Fig. 14d. The ML tree inferred by RAxML based on concatenated 46 plastid genes of 86 species with percentage of parsimony informative sites less than 50%. Numbers associated with branches are bootstrap values; an asterisk indicates bootstrap of 100%. Species from eudicots, monocots, magnoliids, Chloranthaceae and Ceratophyllaceae are in purple, green, orange, violet and blue, respectively.

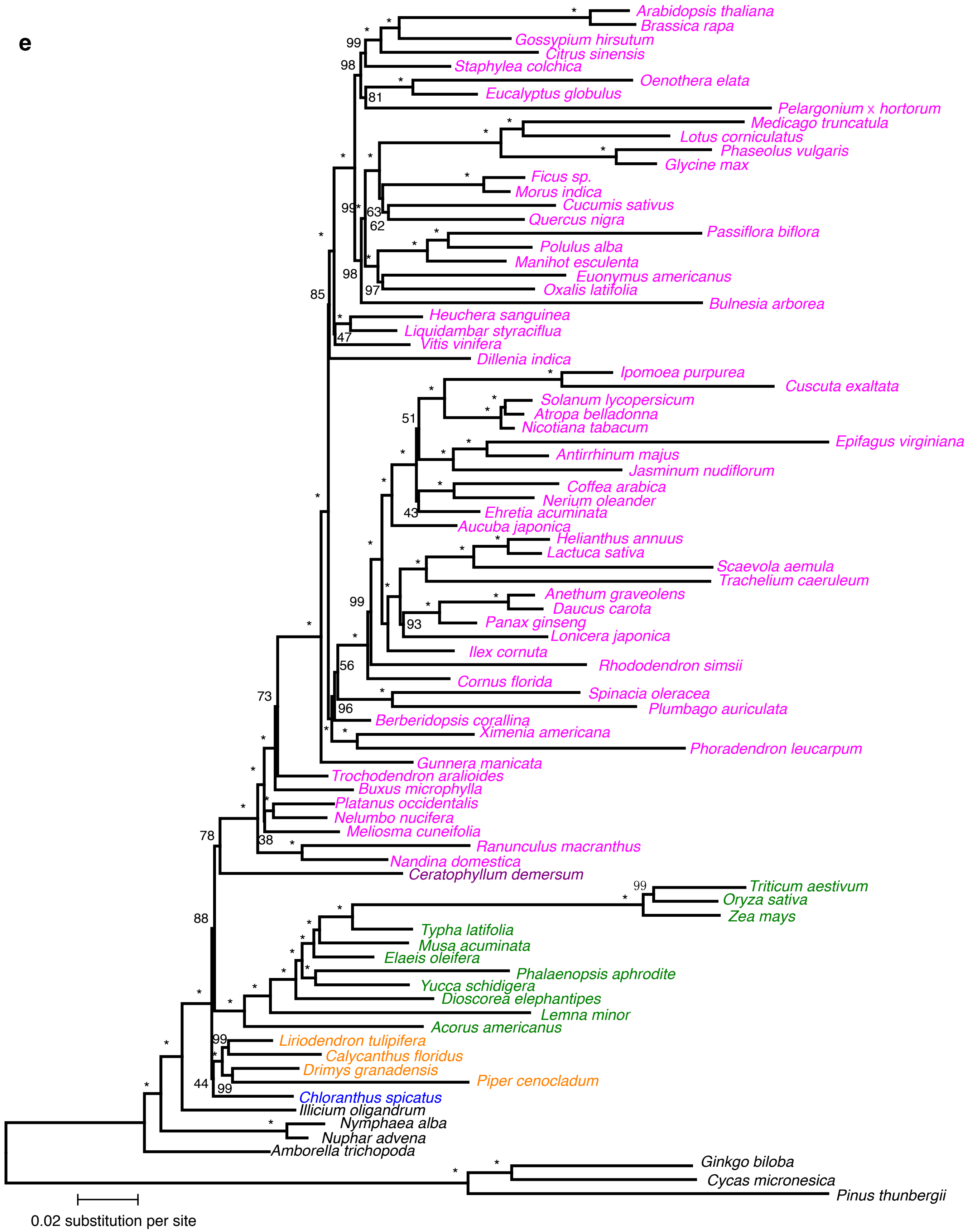

Supplementary Fig. 14e. The ML tree inferred by RAxML based on concatenated 66 plastid genes of 86 species with percentage of parsimony informative sites less than 60%. Numbers associated with branches are bootstrap values; an asterisk indicates bootstrap of 100%. Species from eudicots, monocots, magnoliids, Chloranthaceae and Ceratophyllaceae are in purple, green, orange, violet and blue, respectively.

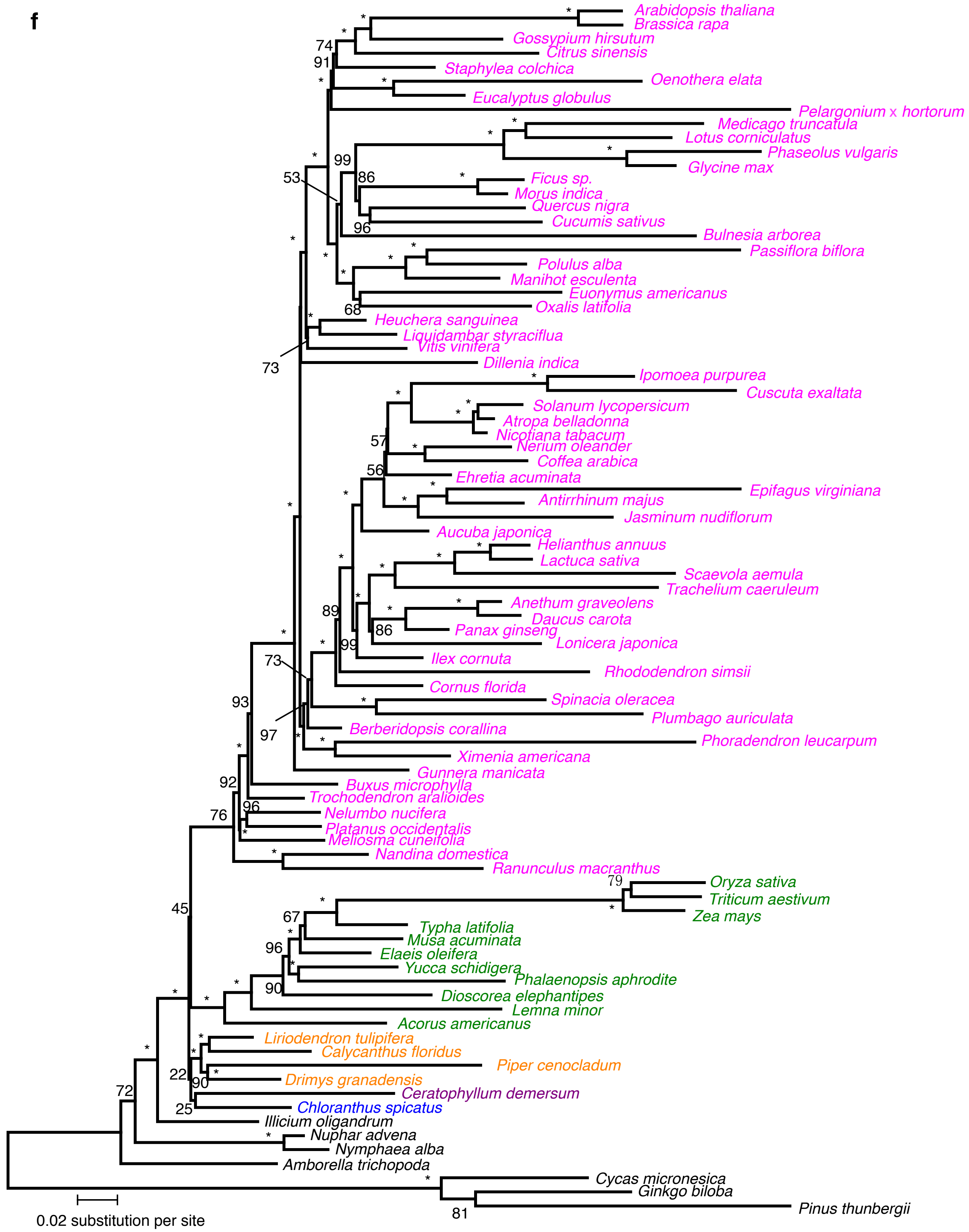

Supplementary Fig. 14f. The ML tree inferred by RAxML based on concatenated 17 plastid genes of 86 species with percentage of parsimony informative sites greater than 60%. Numbers associated with branches are bootstrap values; an asterisk indicates bootstrap of 100%. Species from eudicots, monocots, magnoliids, Chloranthaceae and Ceratophyllaceae are in purple, green, orange, violet and blue, respectively.

g

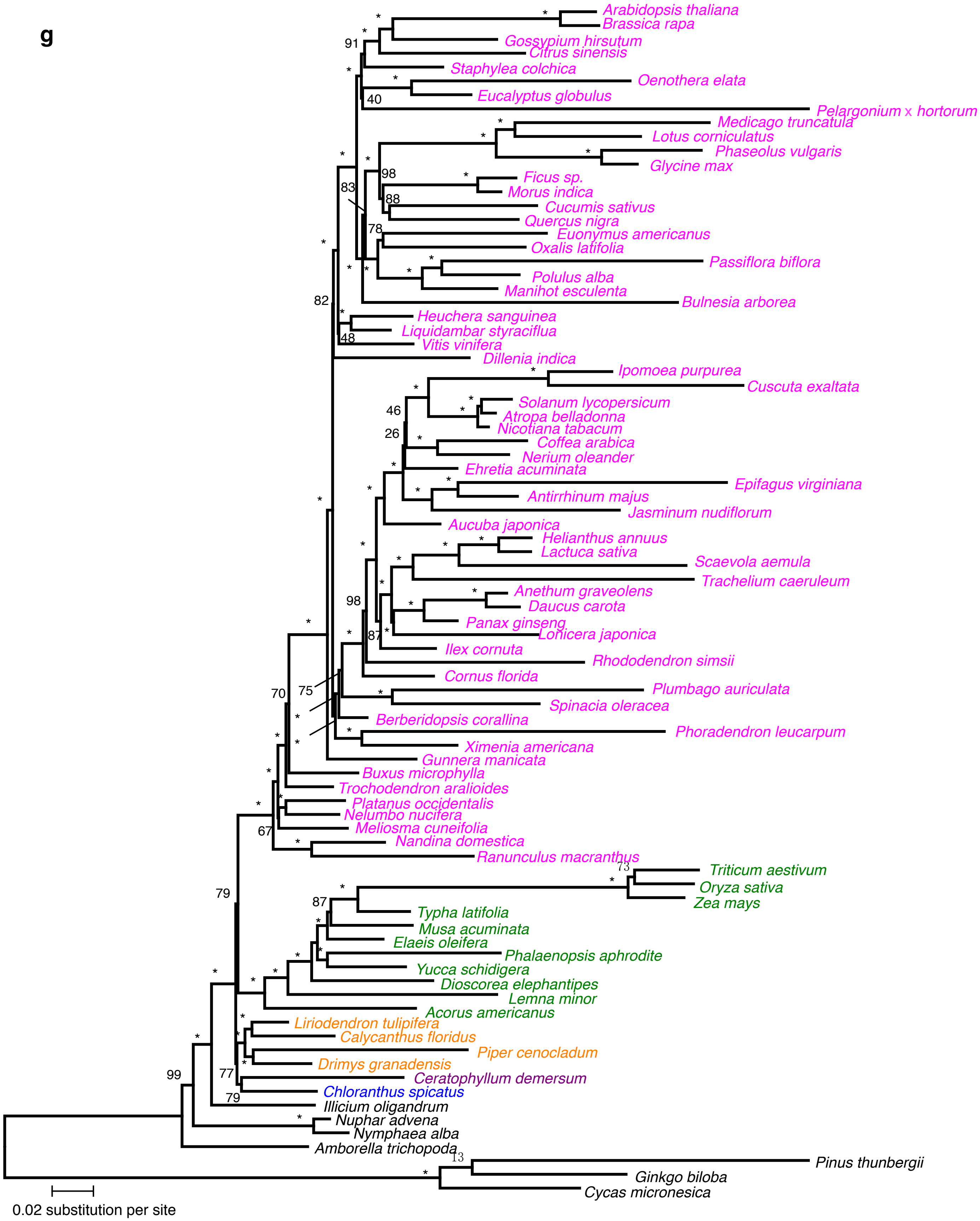

Supplementary Fig. 14g. The ML tree inferred by RAxML based on concatenated 37 plastid genes of 86 species with percentage of parsimony informative sites greater than 50%. Numbers associated with branches are bootstrap values; an asterisk indicates bootstrap of 100%. Species from eudicots, monocots, magnoliids, Chloranthaceae and Ceratophyllaceae are in purple, green, orange, violet and blue, respectively.

h

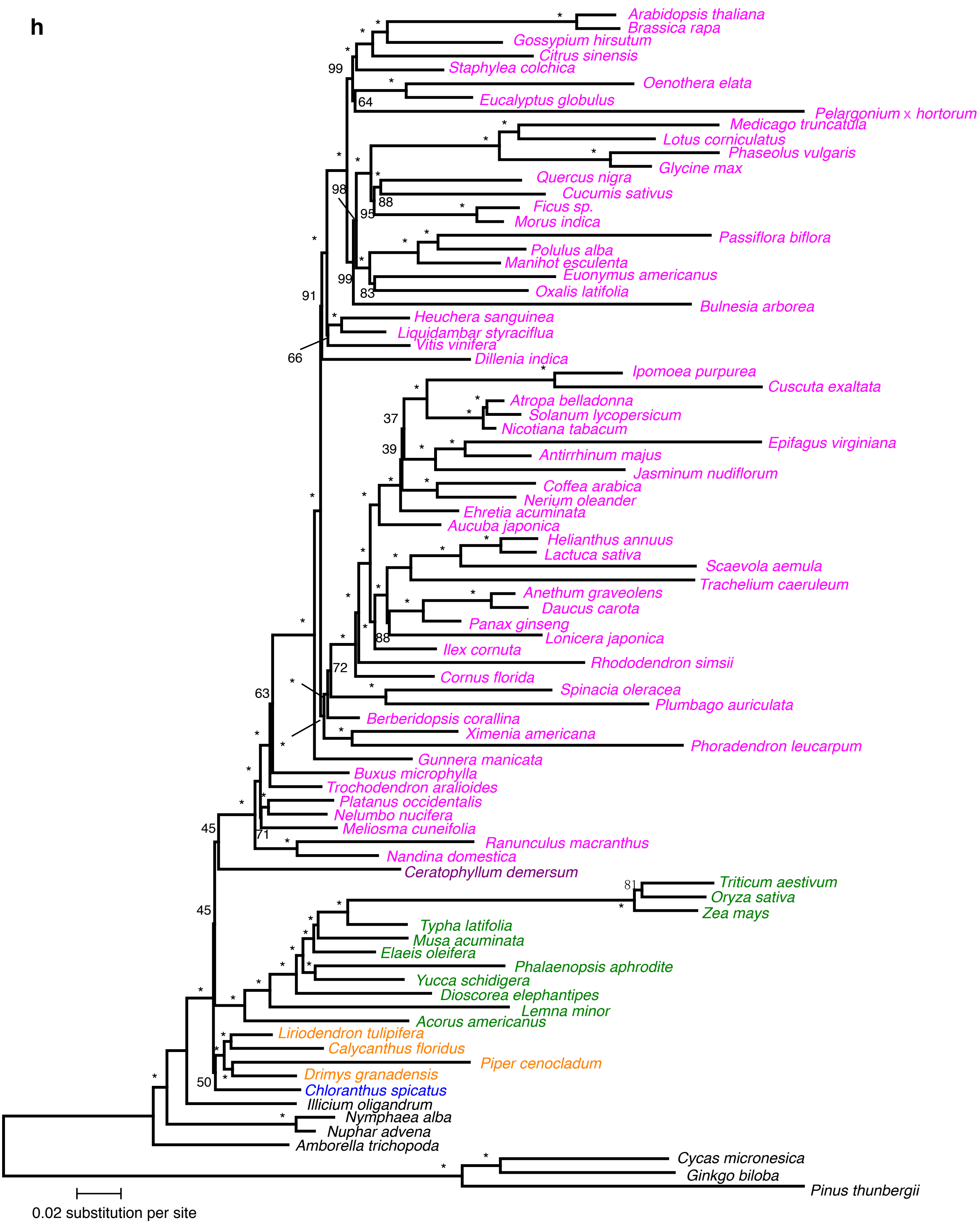

Supplementary Fig. 14h. The ML tree inferred by RAxML based on concatenated 49 plastid genes of 86 species with percentage of parsimony informative sites greater than 40%. Numbers associated with branches are bootstrap values; an asterisk indicates bootstrap of 100%. Species from eudicots, monocots, magnoliids, Chloranthaceae and Ceratophyllaceae are in purple, green, orange, violet and blue, respectively.

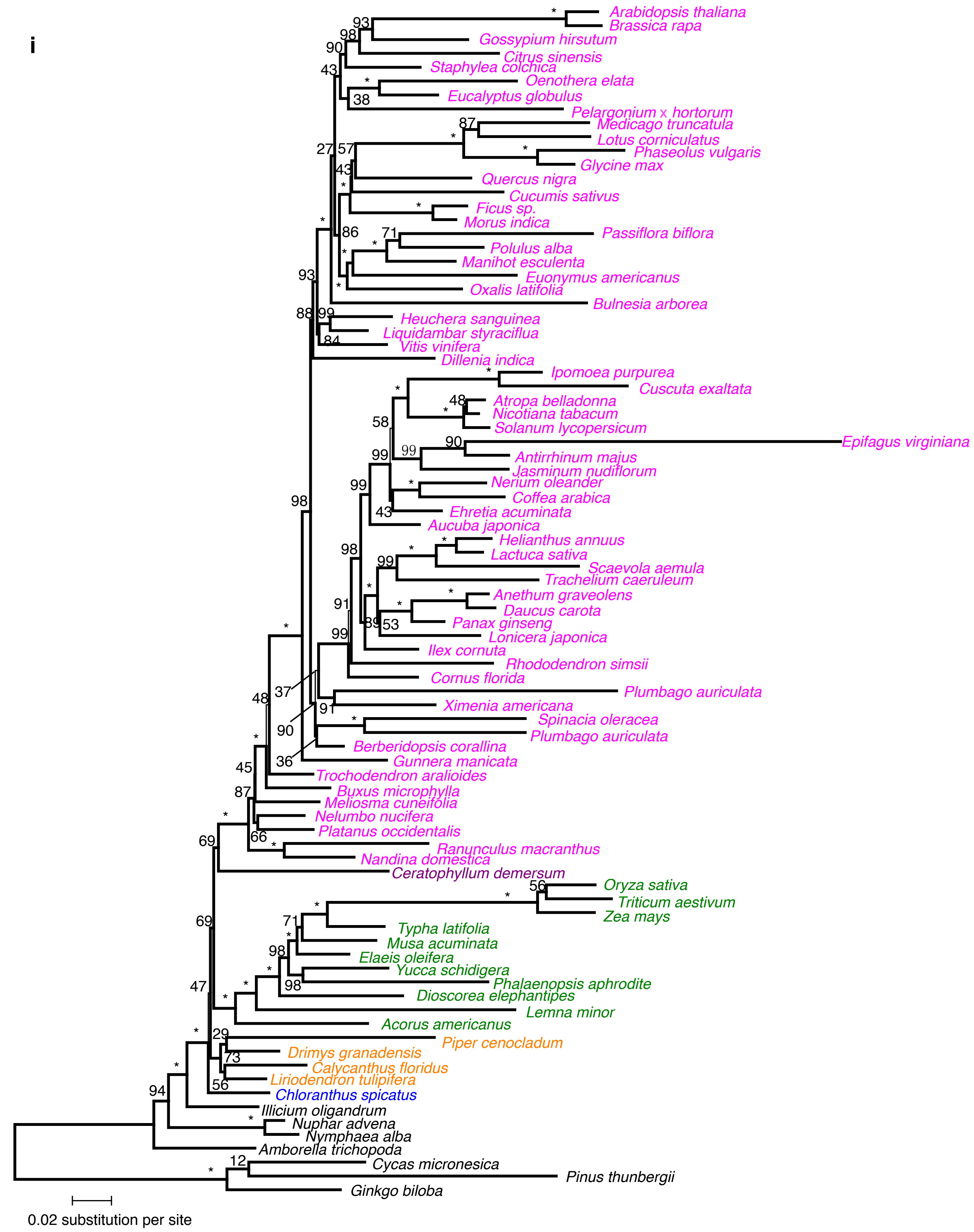

Supplementary Fig. 14i. The ML tree inferred by RAxML based on concatenated 13 plastid genes of 86 species with percentage of parsimony informative sites ranging from 40% to 50%. Numbers associated with branches are bootstrap values; an asterisk indicates bootstrap of 100%. Species from eudicots, monocots, magnoliids, Chloranthaceae and Ceratophyllaceae are in purple, green, orange, violet and blue, respectively.

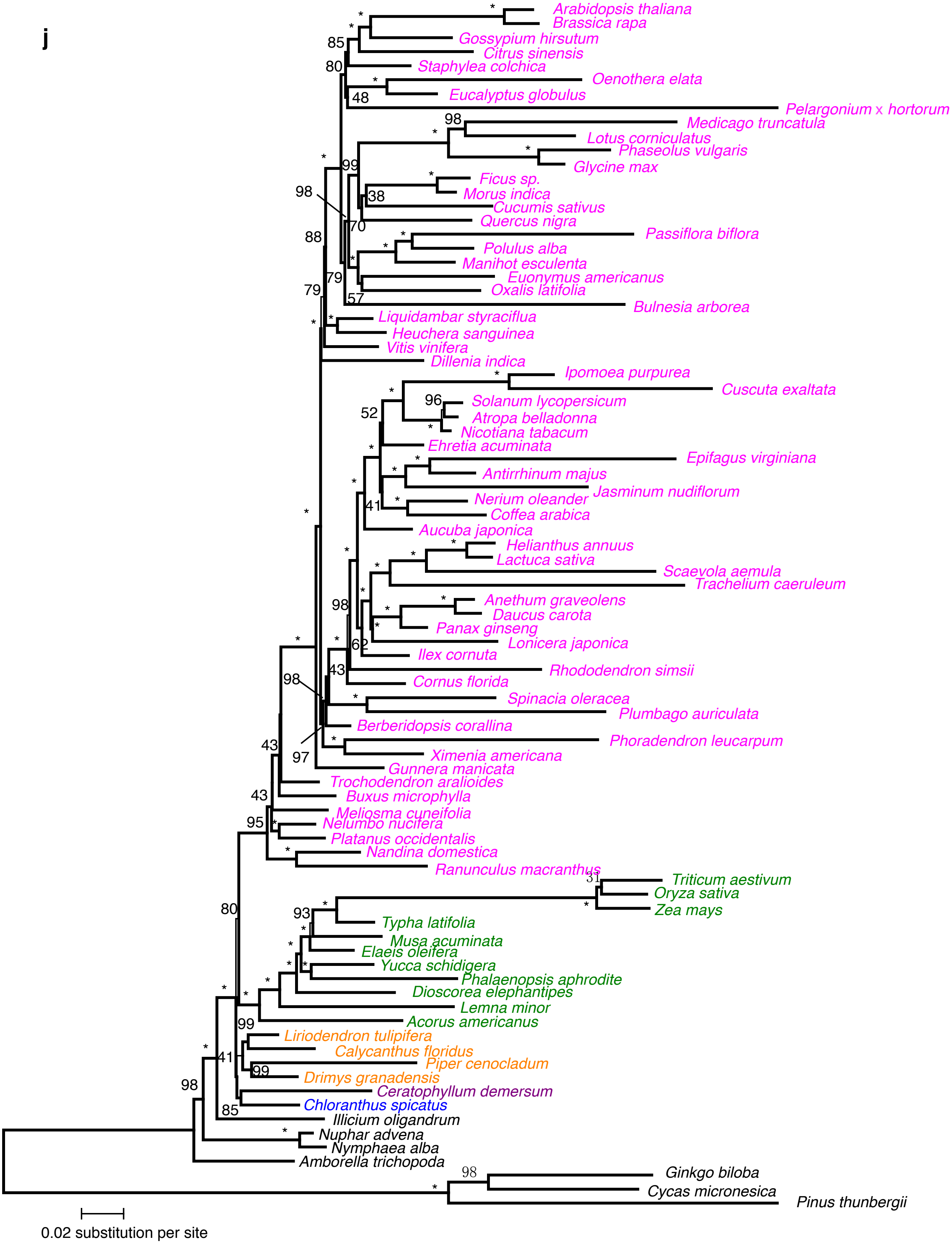

Supplementary Fig. 14j. The ML tree inferred by RAxML based on concatenated 20 plastid genes of 86 species with percentage of parsimony informative sites ranging from 50% to 60%. Numbers associated with branches are bootstrap values; an asterisk indicates bootstrap of 100%. Species from eudicots, monocots, magnoliids, Chloranthaceae and Ceratophyllaceae are in purple, green, orange, violet and blue, respectively.

k

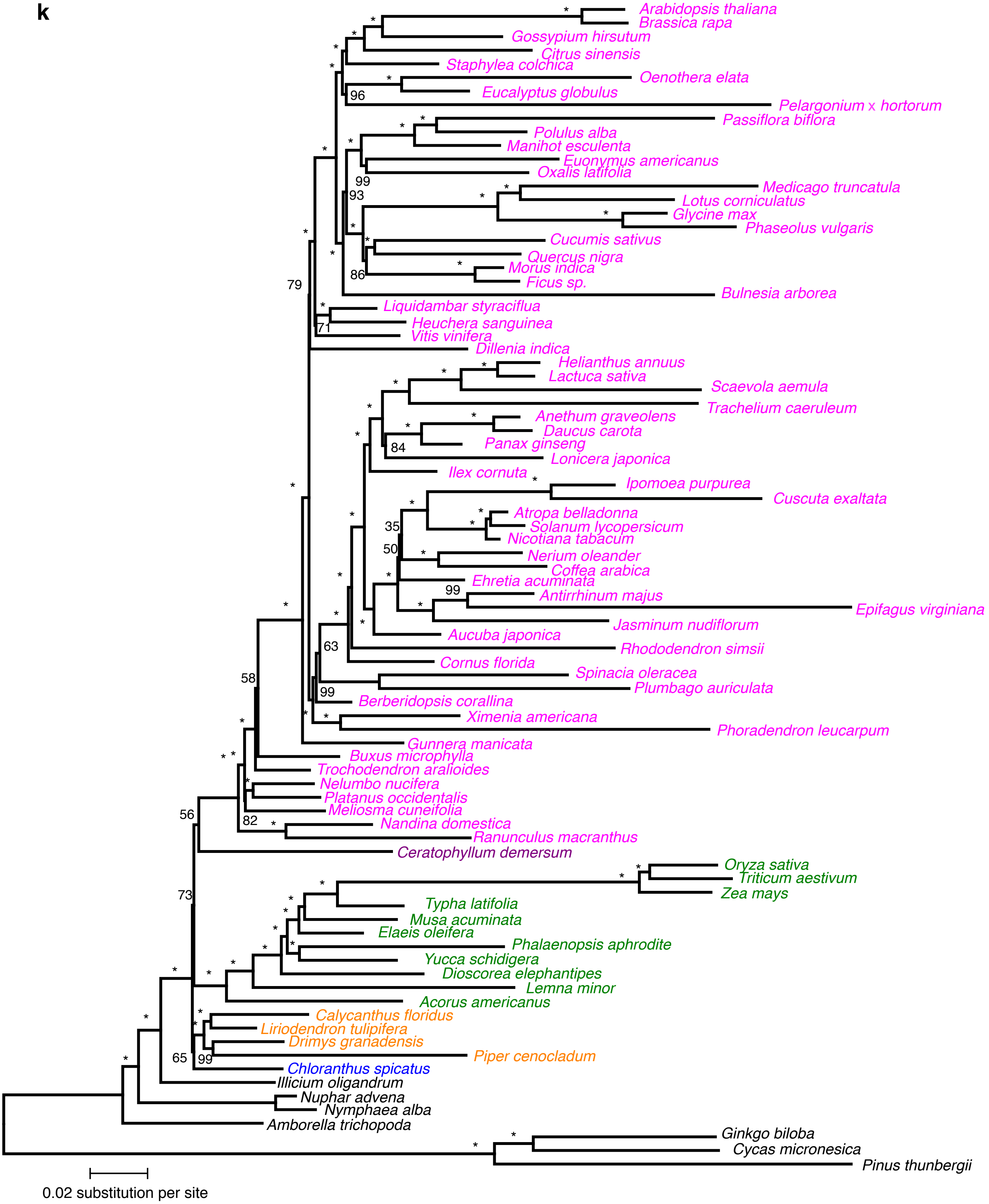

Supplementary Fig. 14k. The ML tree inferred by RAxML based on concatenated 112 plastid genes of 86 species. Numbers associated with branches are bootstrap values; an asterisk indicates bootstrap of 100%. Species from eudicots, monocots, magnoliids, Chloranthaceae and Ceratophyllaceae are in purple, green, orange, violet and blue, respectively.

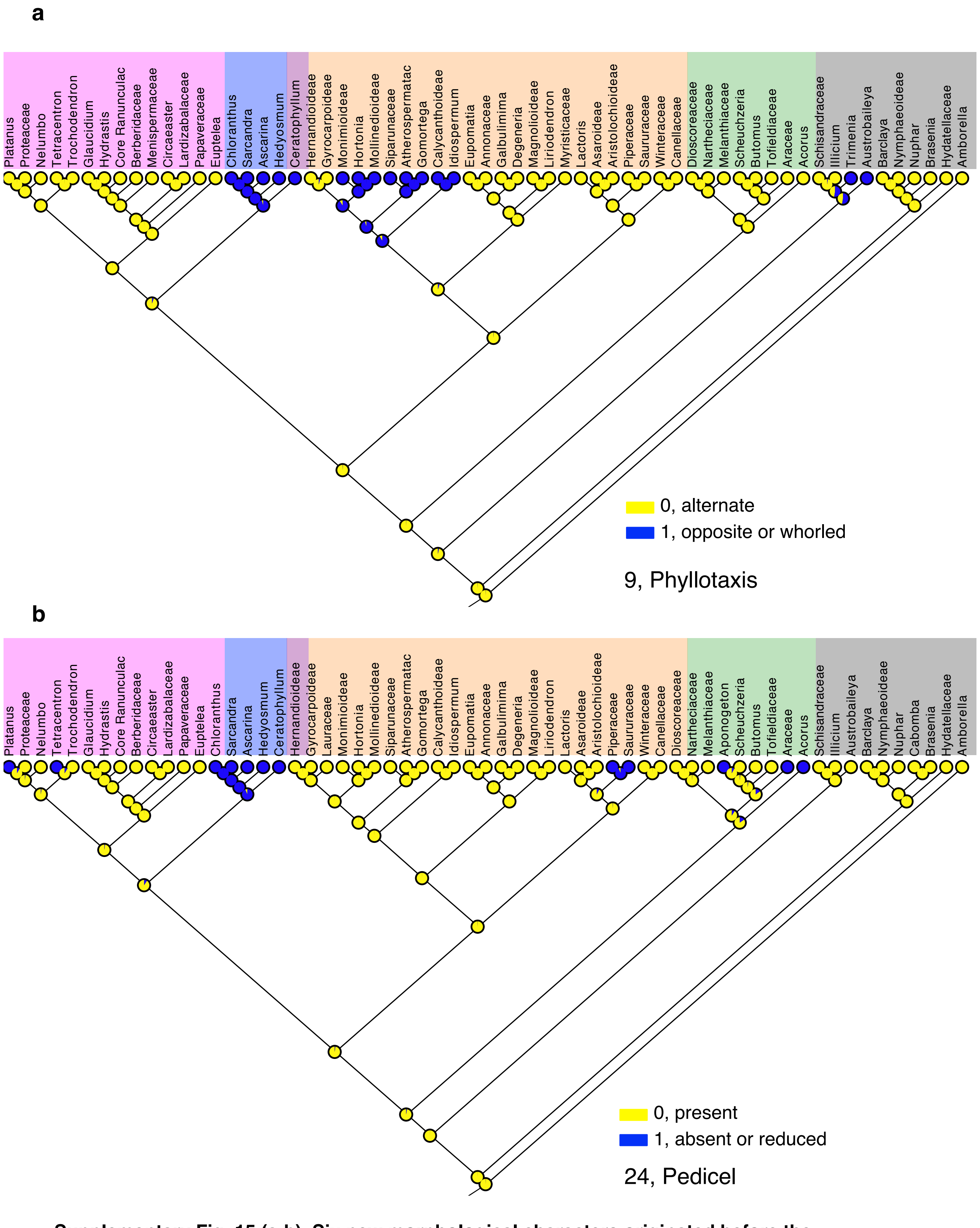

Supplementary Fig. 15 (a-b). Six new morphological characters originated before the diversification of Chloranthaceae and Ceratophyllaceae. Eudicots, Chloranthaceae, Ceratophyllaceae, magnoliids, monocots and basal angiosperms are highlighted by pink, blue, dark purple, orange, green and gray, respectively.

c

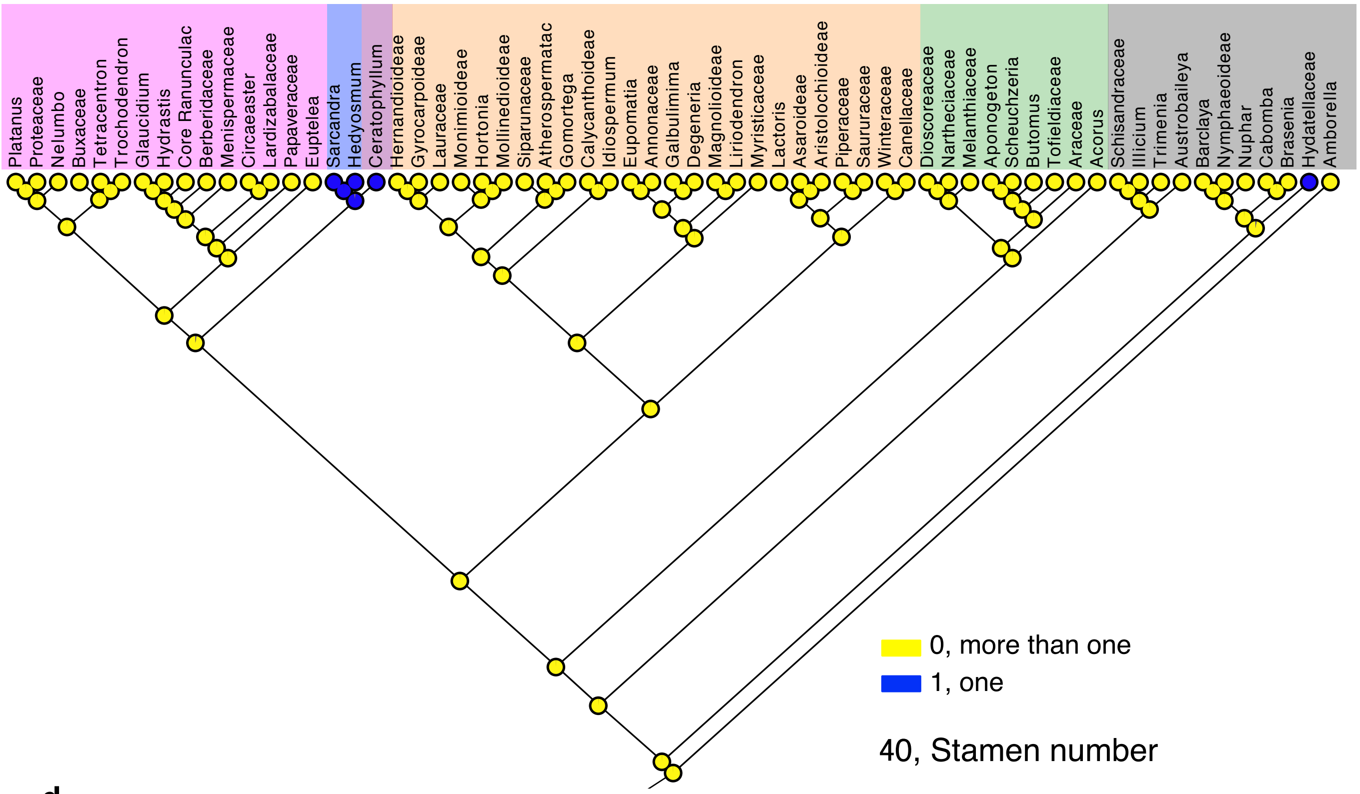

d

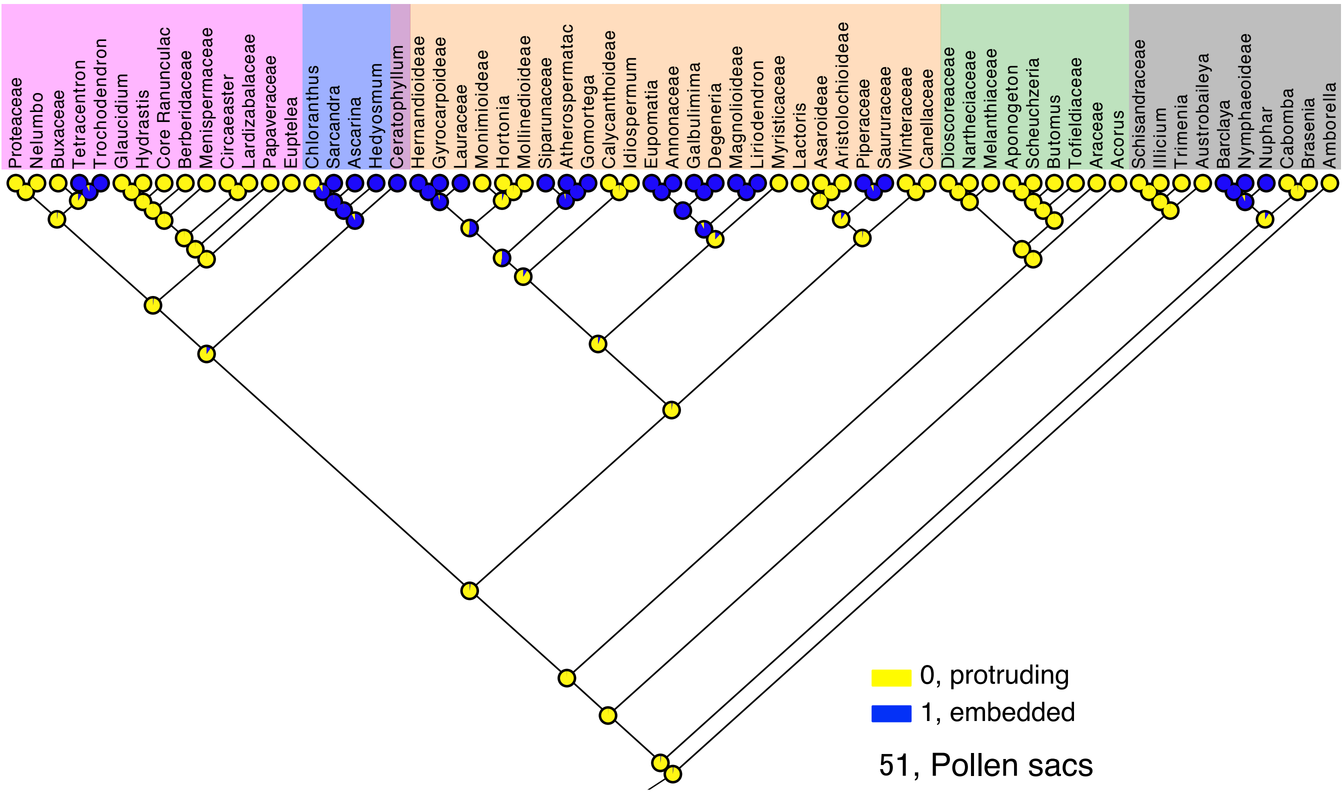

Supplementary Fig. 15 (c-d). Six new morphological characters originated before the diversification of Chloranthaceae and Ceratophyllaceae. Eudicots, Chloranthaceae, Ceratophyllaceae, magnoliids, monocots and basal angiosperms are highlighted by pink, blue, dark purple, orange, green and gray, respectively.

e

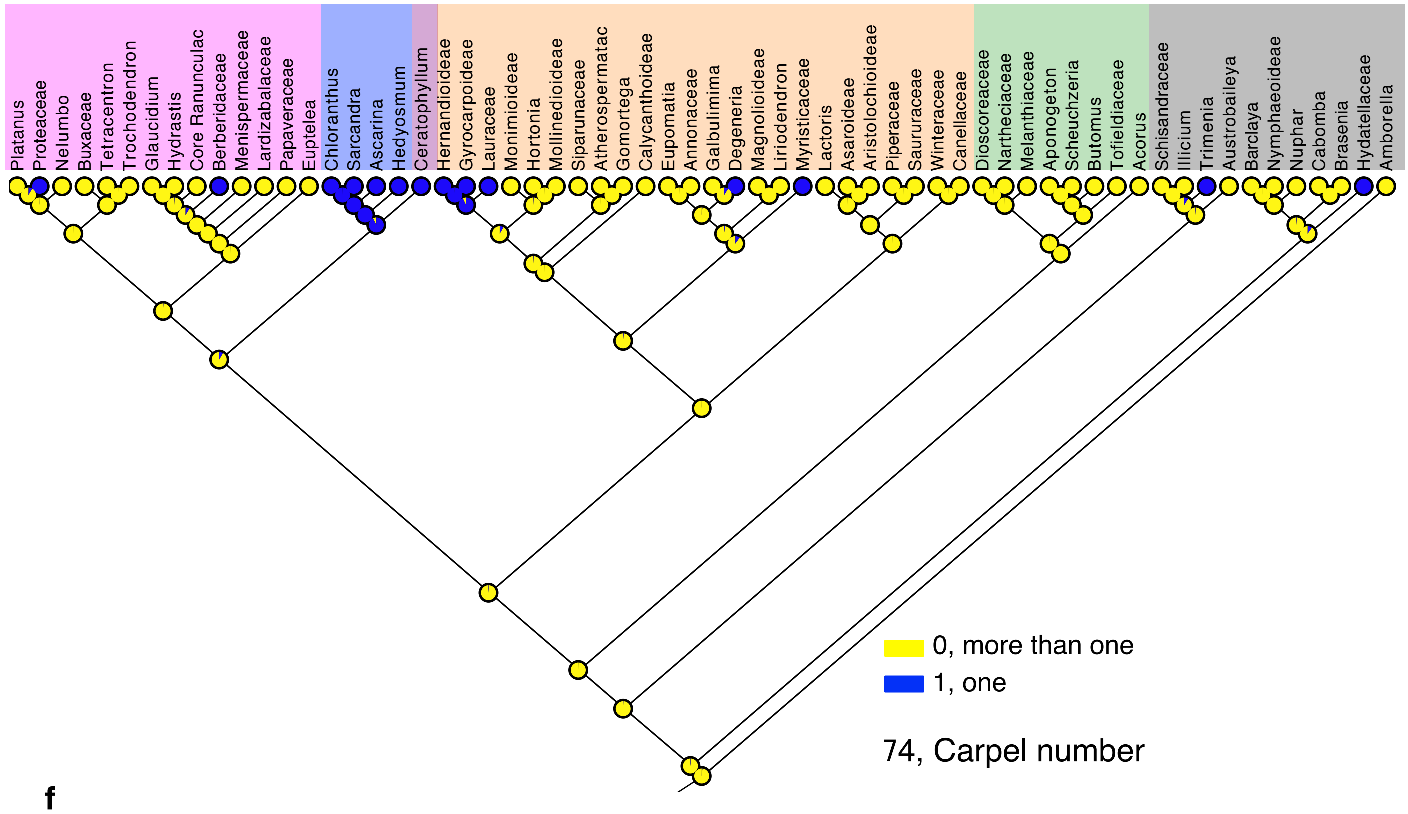

f

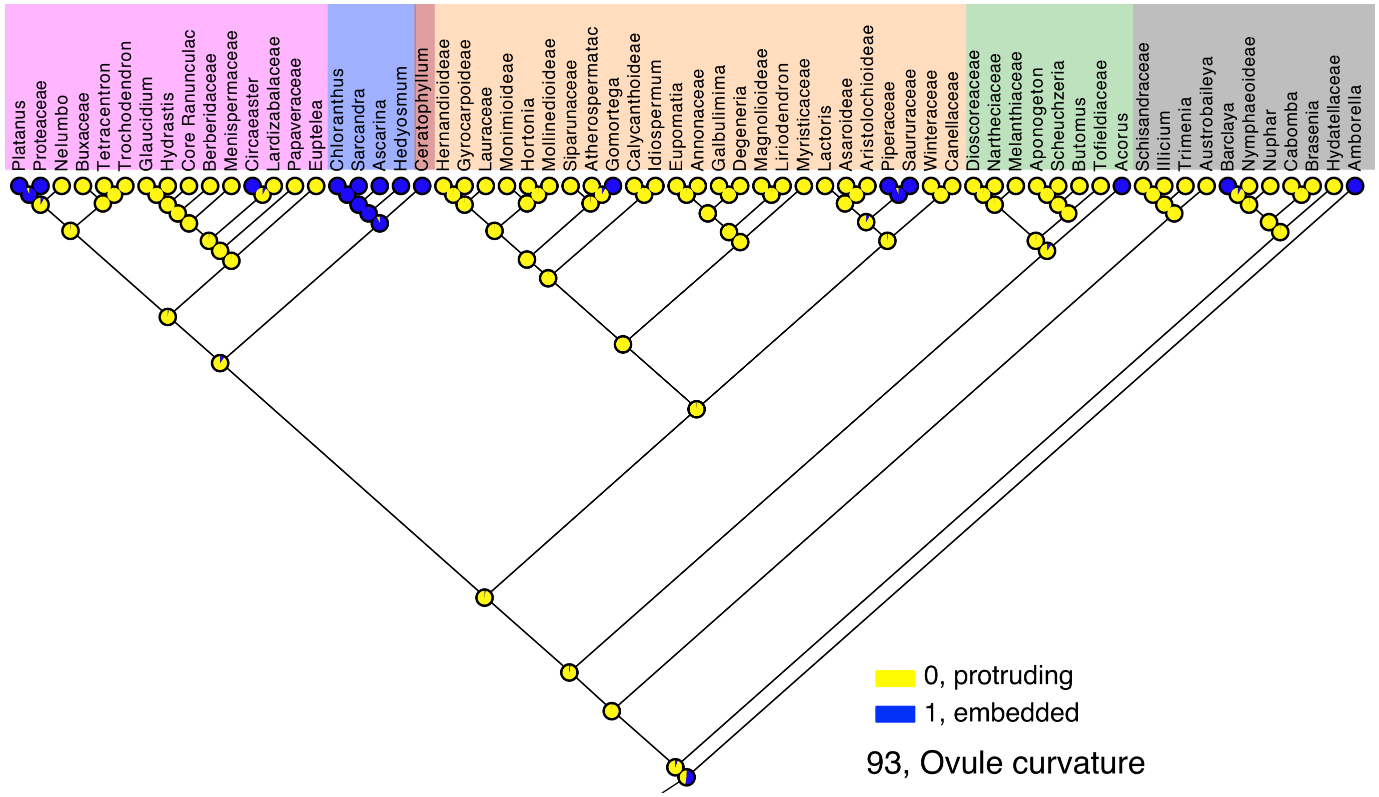

Supplementary Fig. 15 (e-f). Six new morphological characters originated before the diversification of Chloranthaceae and Ceratophyllaceae. Eudicots, Chloranthaceae, Ceratophyllaceae, magnoliids, monocots and basal angiosperms are highlighted by pink, blue, dark purple, orange, green and gray, respectively.

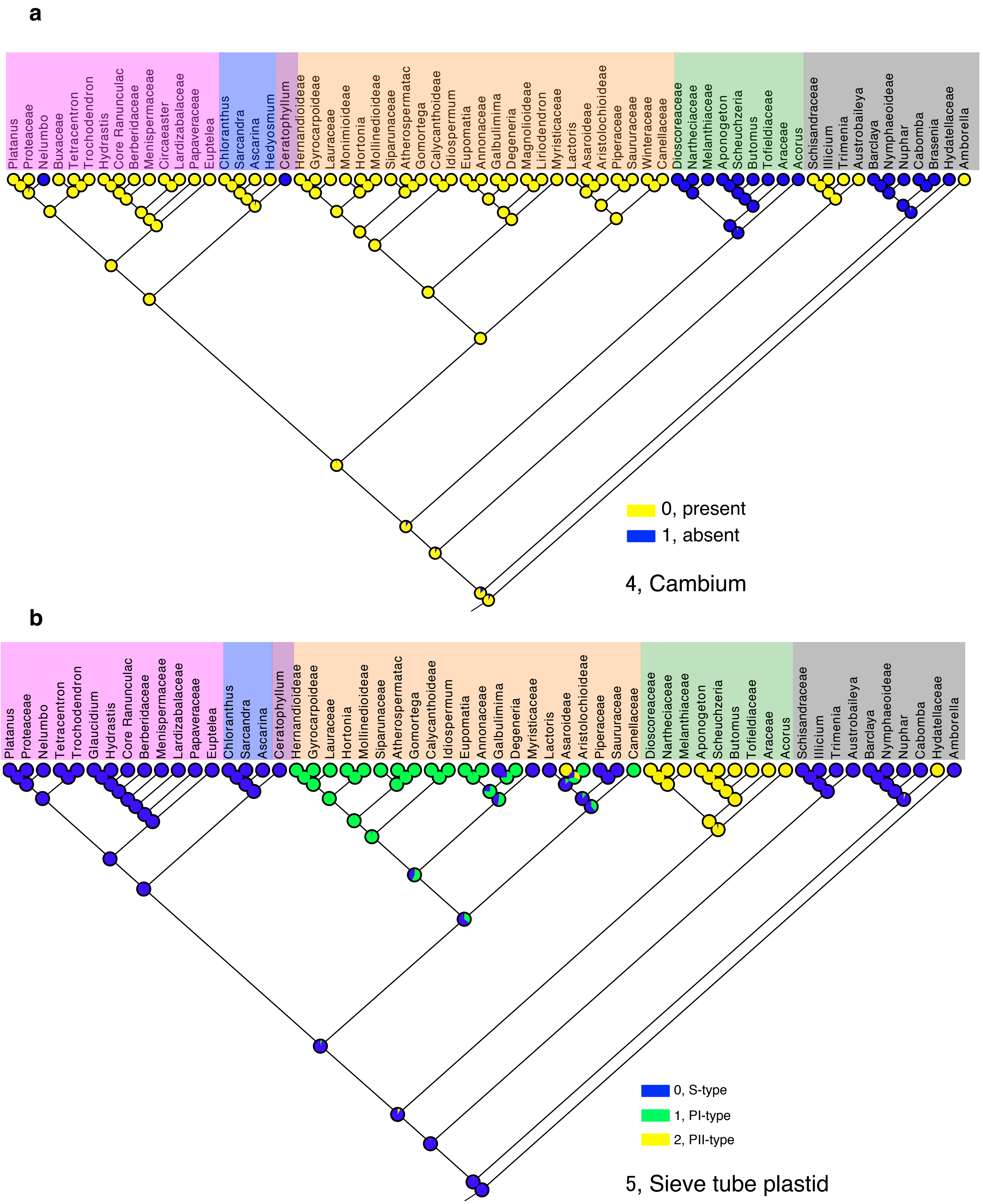

Supplementary Fig. 16 (a-b). Seven new morphological characters originated before the diversification of monocots. Eudicots, Chloranthaceae, Ceratophyllaceae, magnoliids, monocots and basal angiosperms are highlighted by pink, blue, dark purple, orange, green and gray, respectively.

c

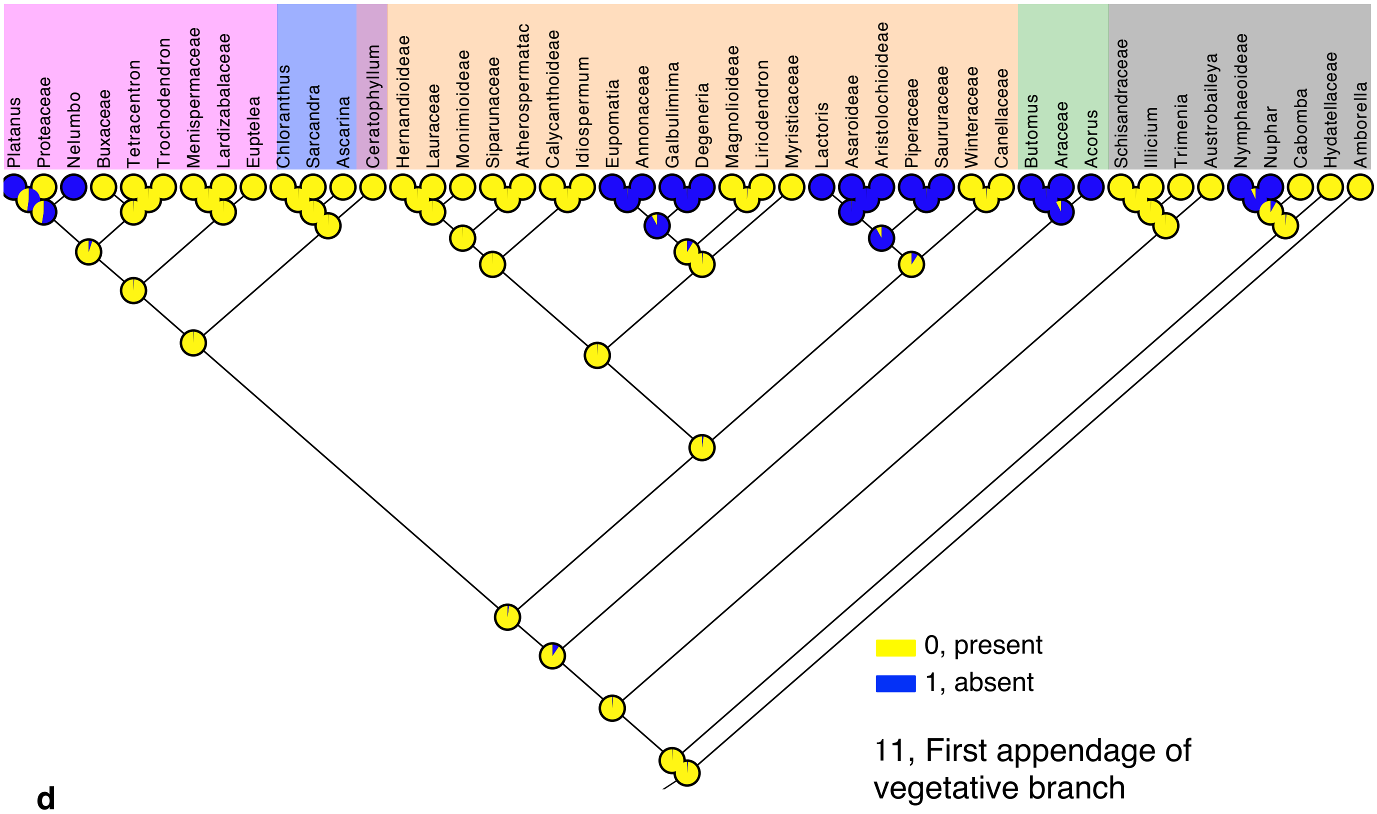

d

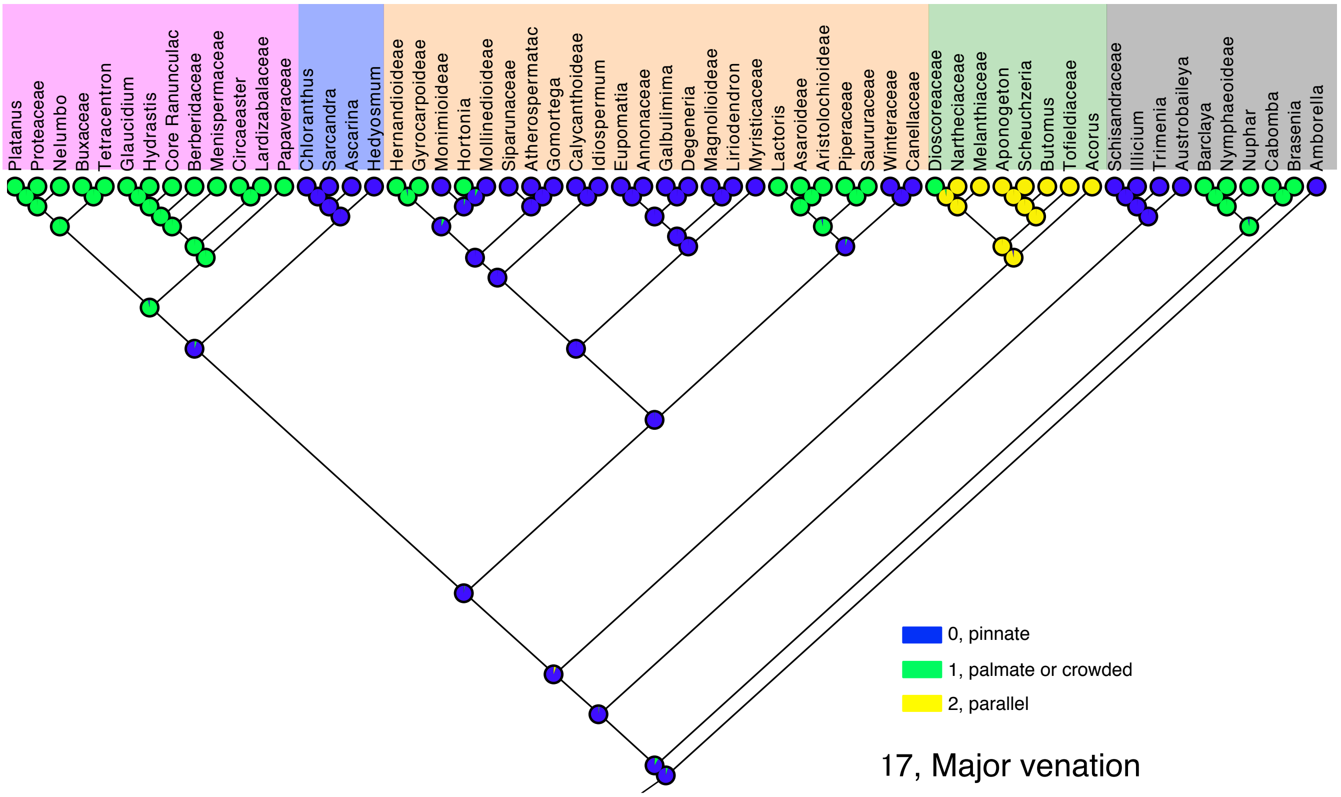

Supplementary Fig. 16 (c-d). Seven new morphological characters originated before the diversification of monocots. Eudicots, Chloranthaceae, Ceratophyllaceae, magnoliids, monocots and basal angiosperms are highlighted by pink, blue, dark purple, orange, green and gray, respectively.

e

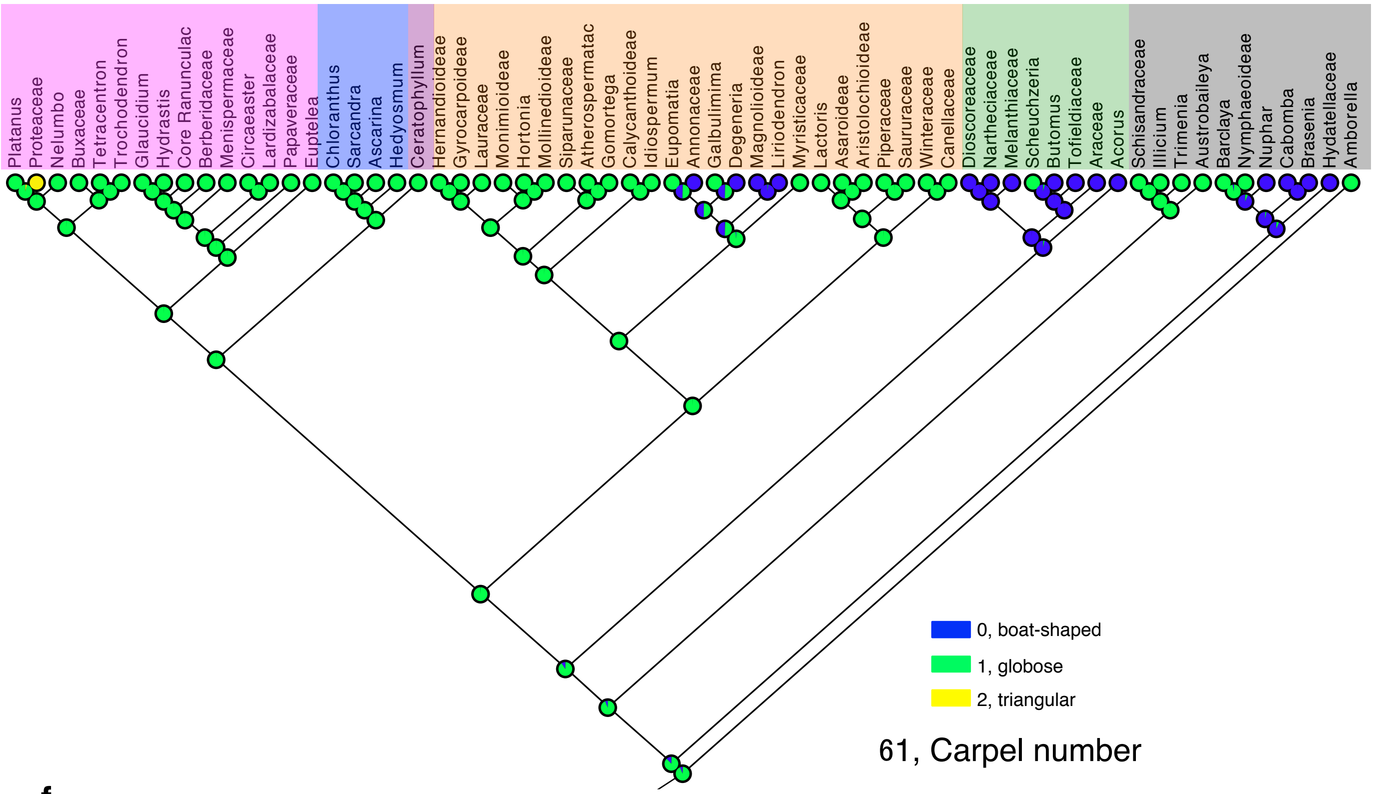

f

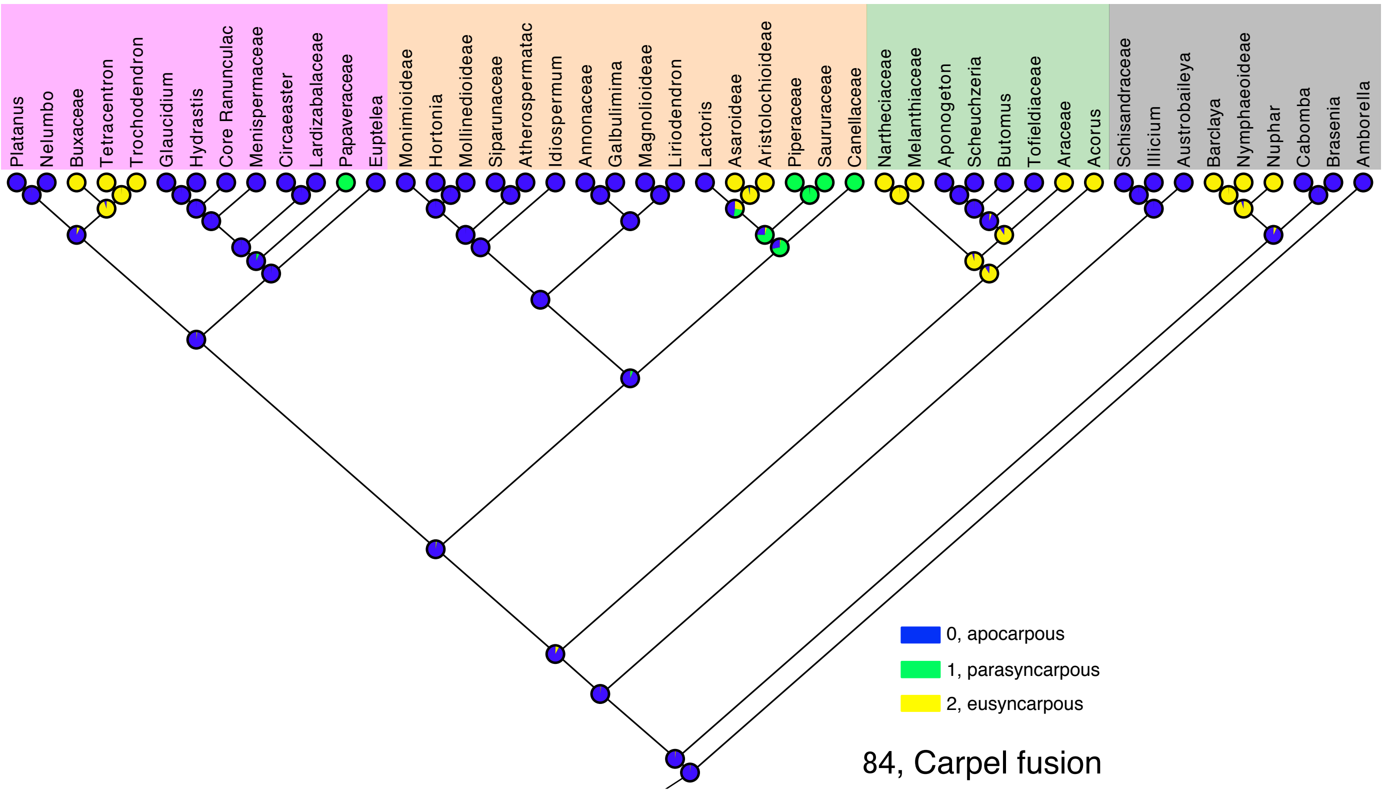

Supplementary Fig. 16 (e-f). Seven new morphological characters originated before the diversification of monocots. Eudicots, Chloranthaceae, Ceratophyllaceae, magnoliids, monocots and basal angiosperms are highlighted by pink, blue, dark purple, orange, green and gray, respectively.

g

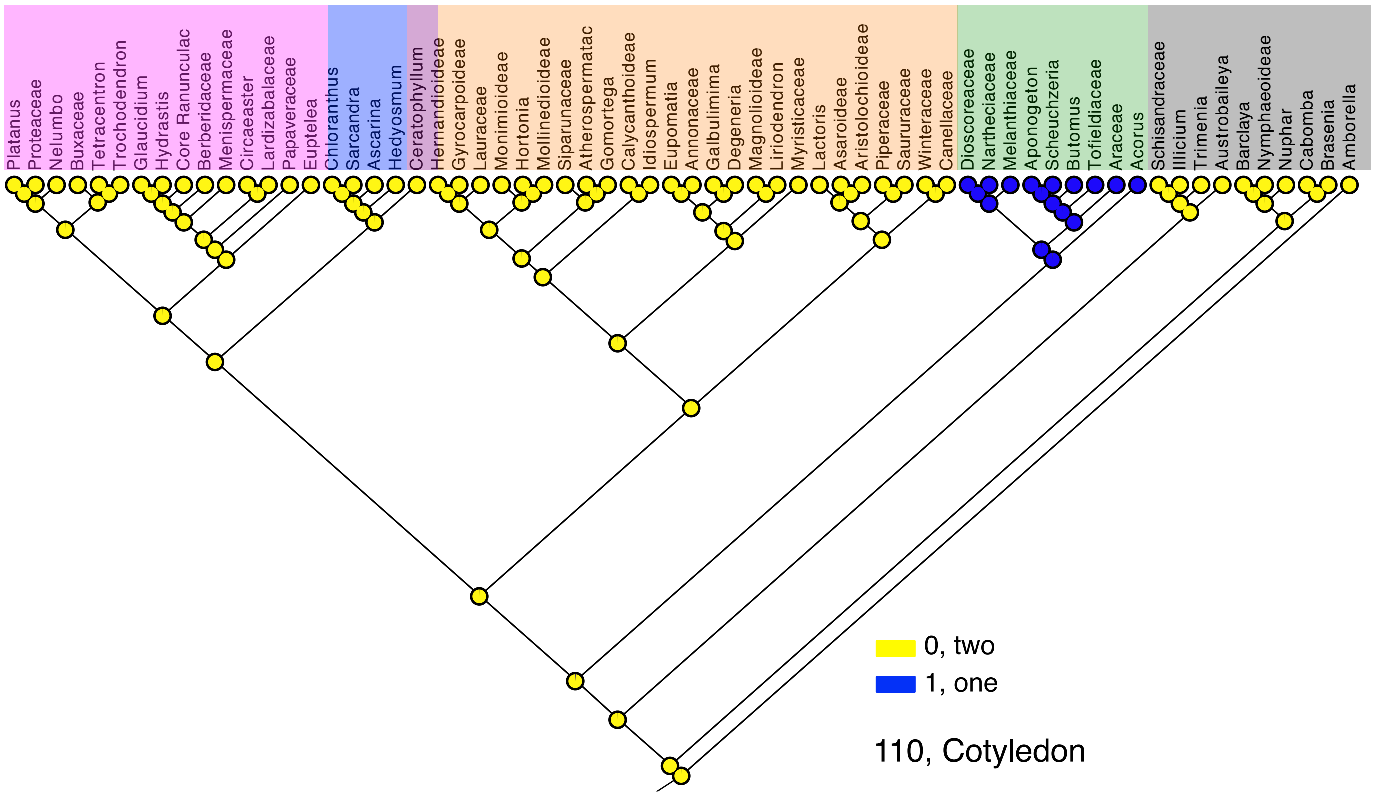

Supplementary Fig. 16 (g). Seven new morphological characters originated before the diversification of monocots. Eudicots, Chloranthaceae, Ceratophyllaceae, magnoliids, monocots and basal angiosperms are highlighted by pink, blue, dark purple, orange, green and gray, respectively.

a

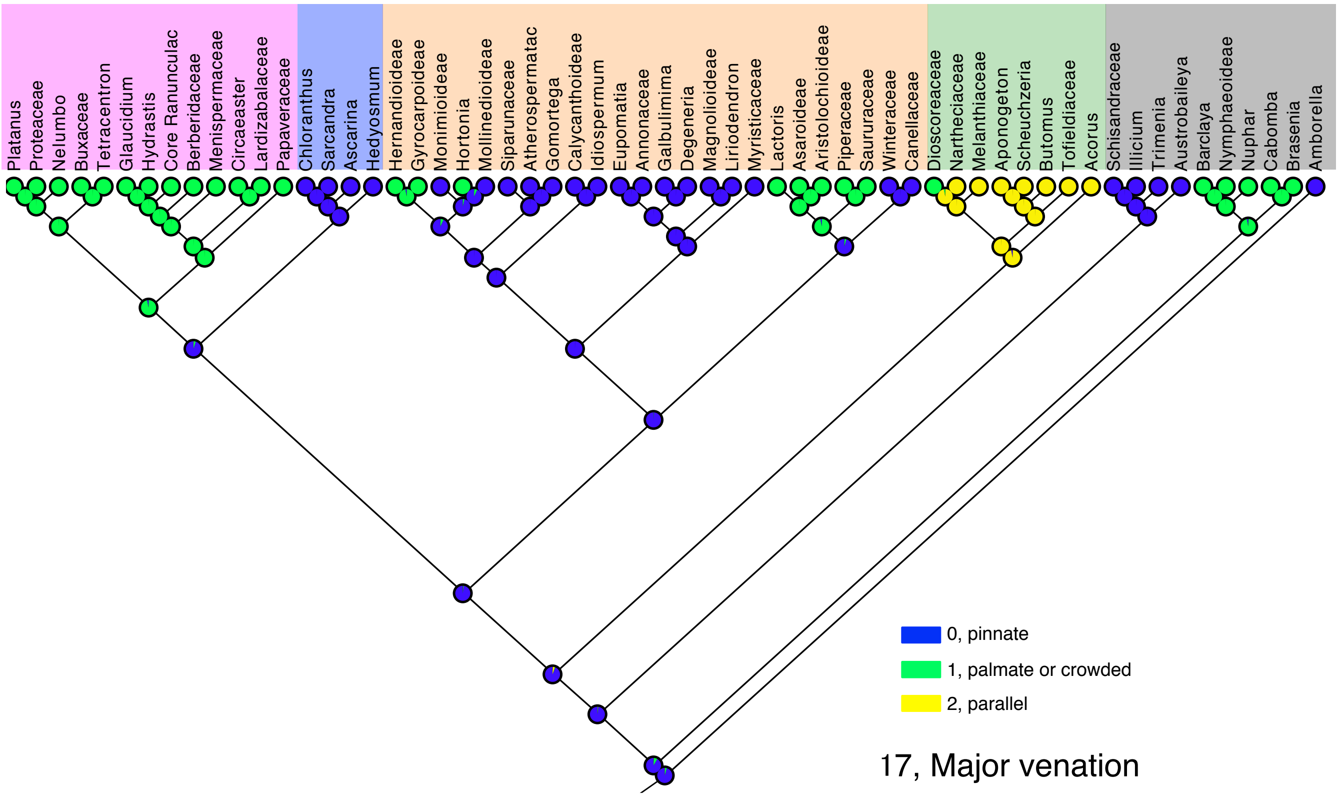

b

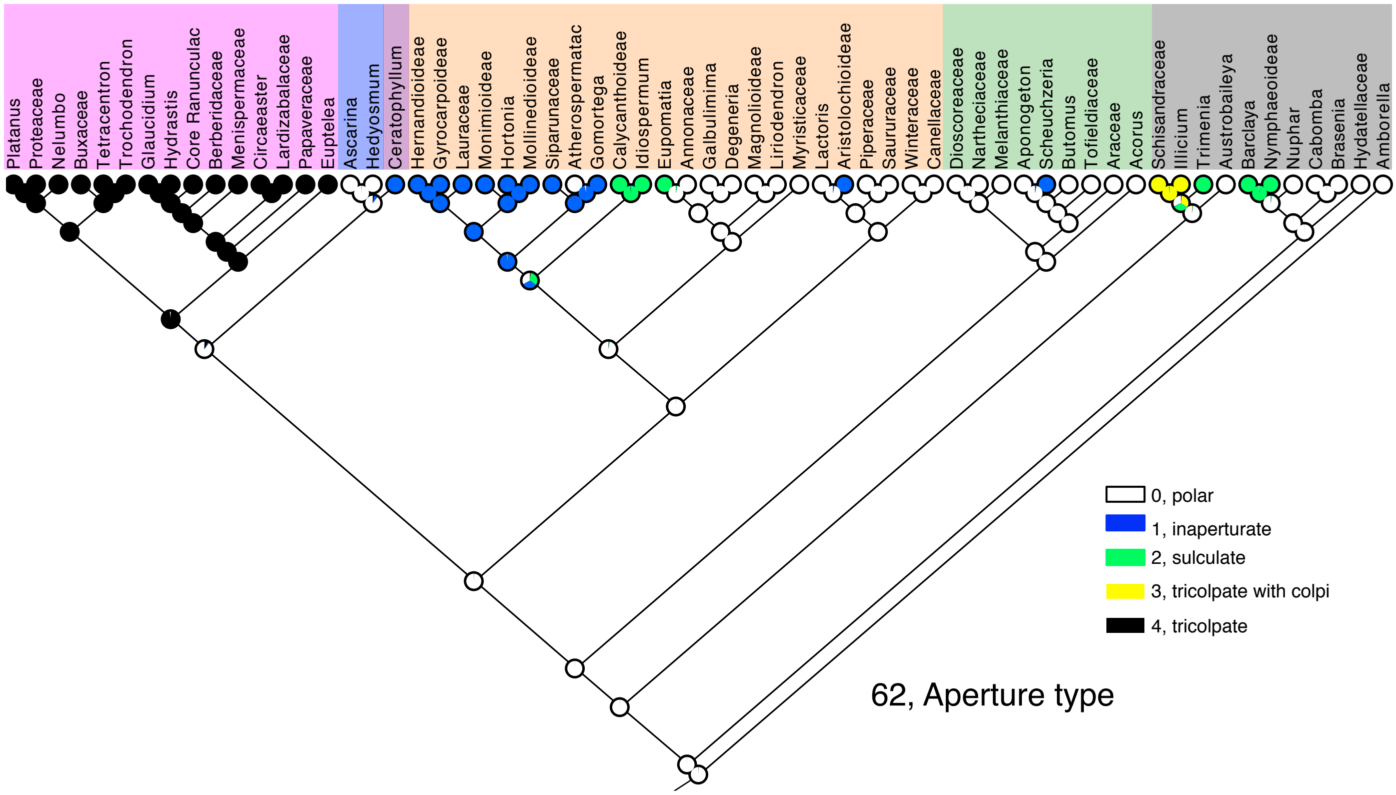

Supplementary Fig. 17 (a-b). Two new morphological characters originated before the diversification of eudicots. Eudicots, Chloranthaceae, Ceratophyllaceae, magnoliids, monocots and basal angiosperms are highlighted by pink, blue, dark purple, orange, green and gray, respectively.

a

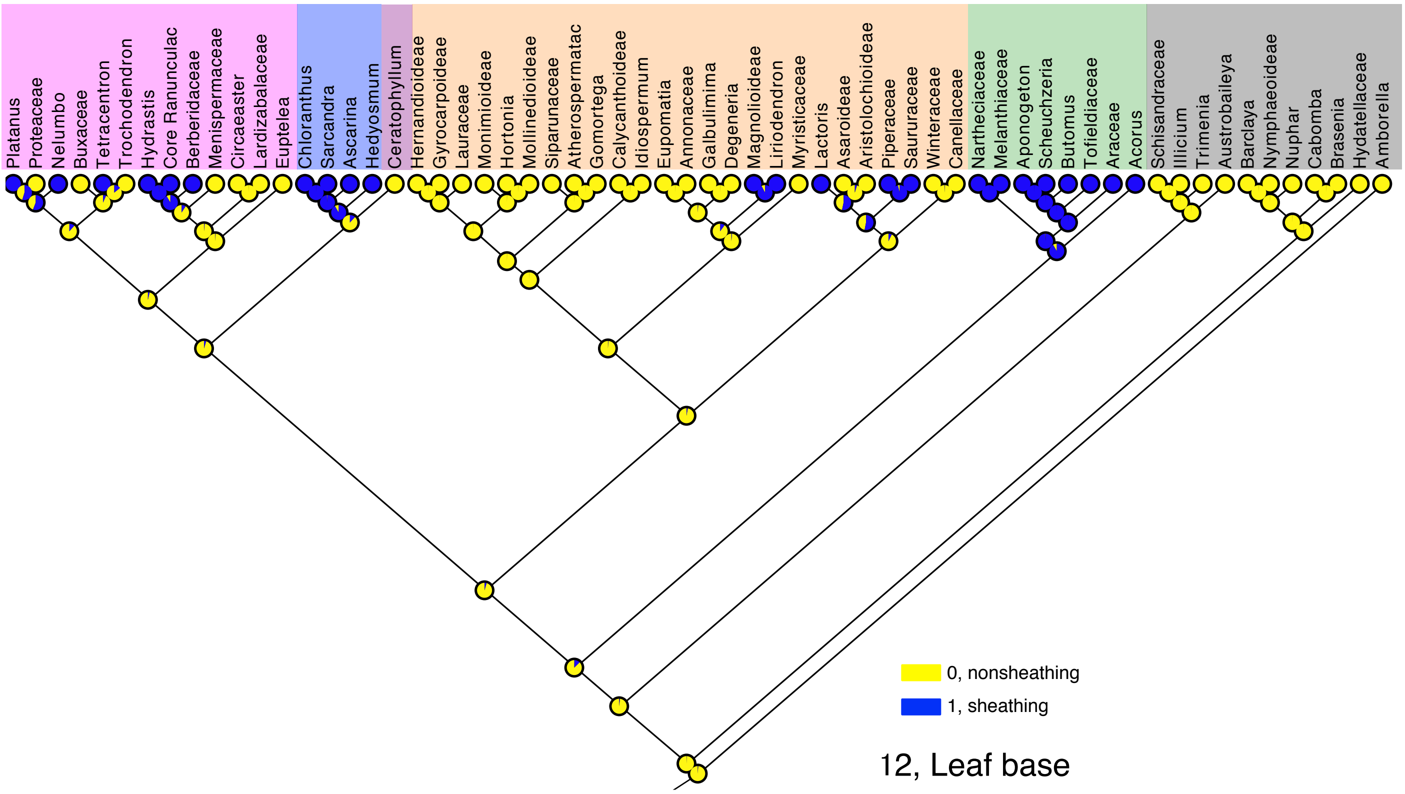

b

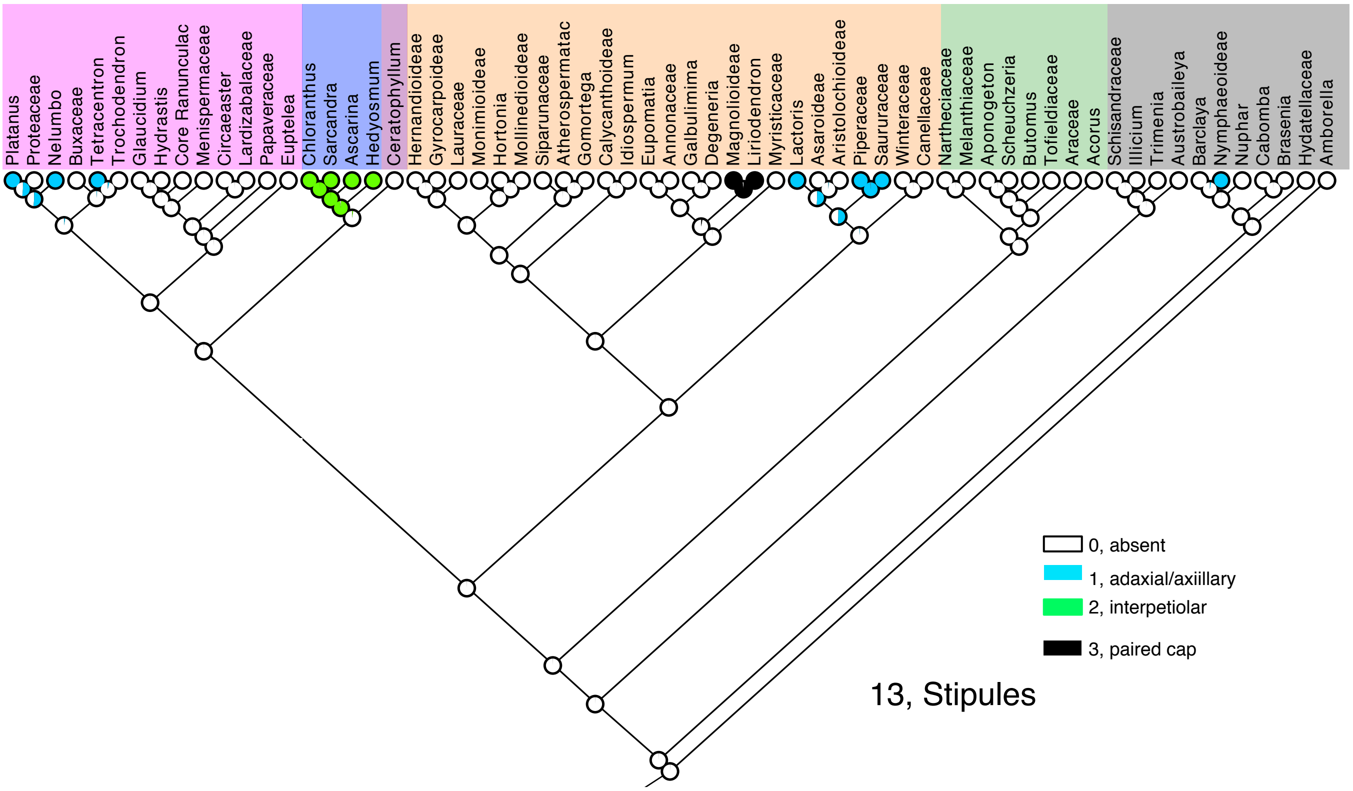

Supplementary Fig. 18 (a-b). Three new morphological characters originated before the diversification of Chloranthaceae. Eudicots, Chloranthaceae, Ceratophyllaceae, magnoliids, monocots and basal angiosperms are highlighted by pink, blue, dark purple, orange, green and gray, respectively.

c

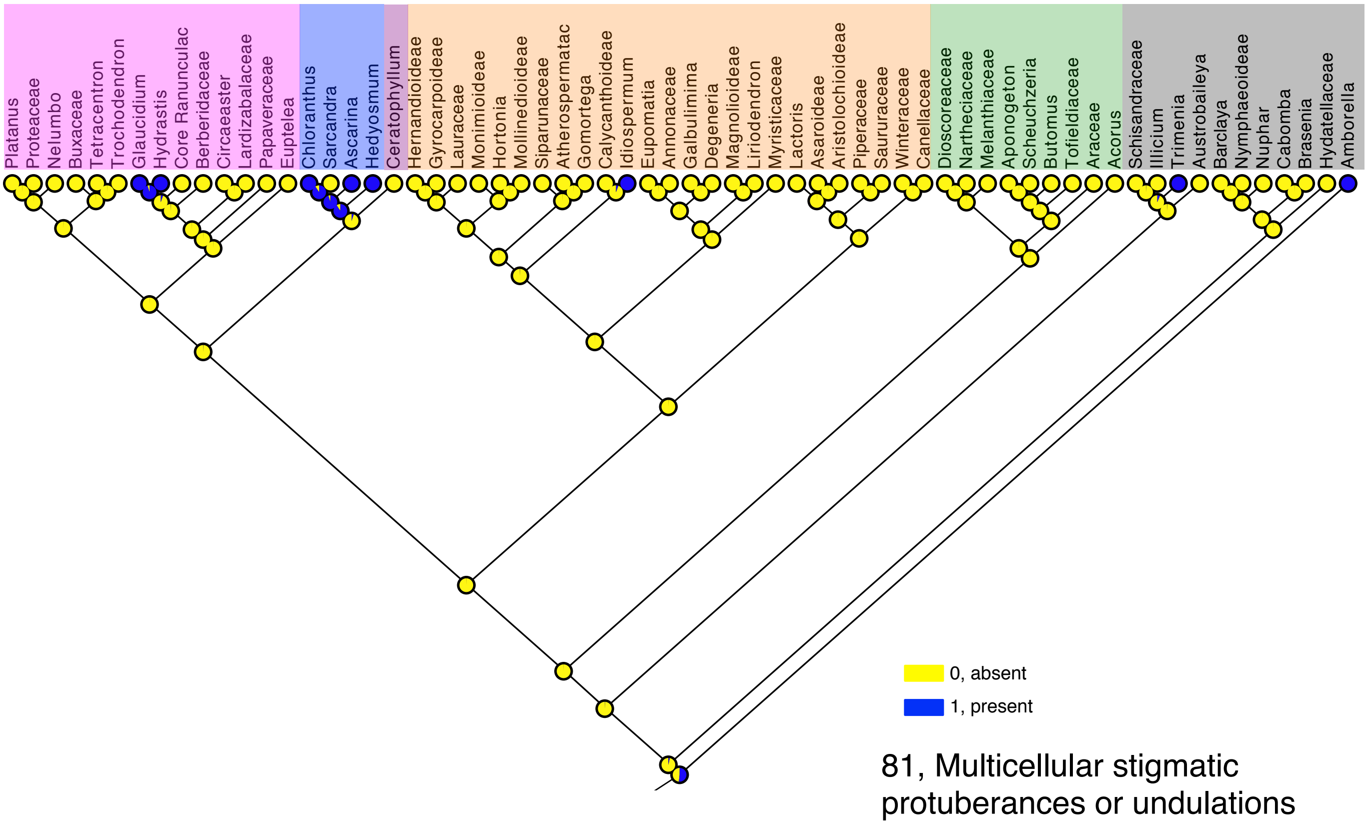

Supplementary Fig. 18 (c). Three new morphological characters originated before the diversification of Chloranthaceae. Eudicots, Chloranthaceae, Ceratophyllaceae, magnoliids, monocots and basal angiosperms are highlighted by pink, blue, dark purple, orange, green and gray, respectively.

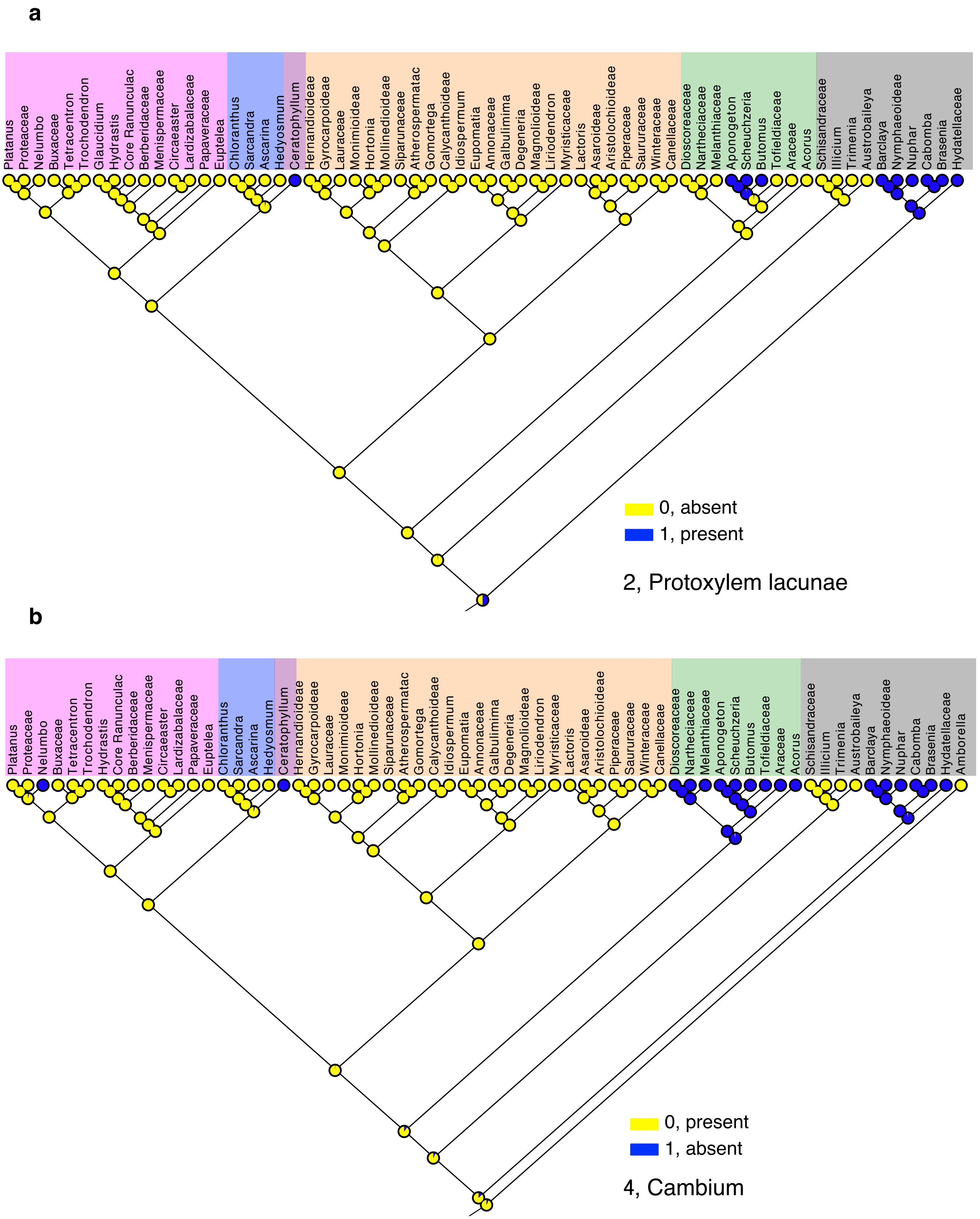

Supplementary Fig. 19 (a-b). Nine new morphological characters originated before the diversification of Ceratophyllaceae. Eudicots, Chloranthaceae, Ceratophyllaceae, magnoliids, monocots and basal angiosperms are highlighted by pink, blue, dark purple, orange, green and gray, respectively.

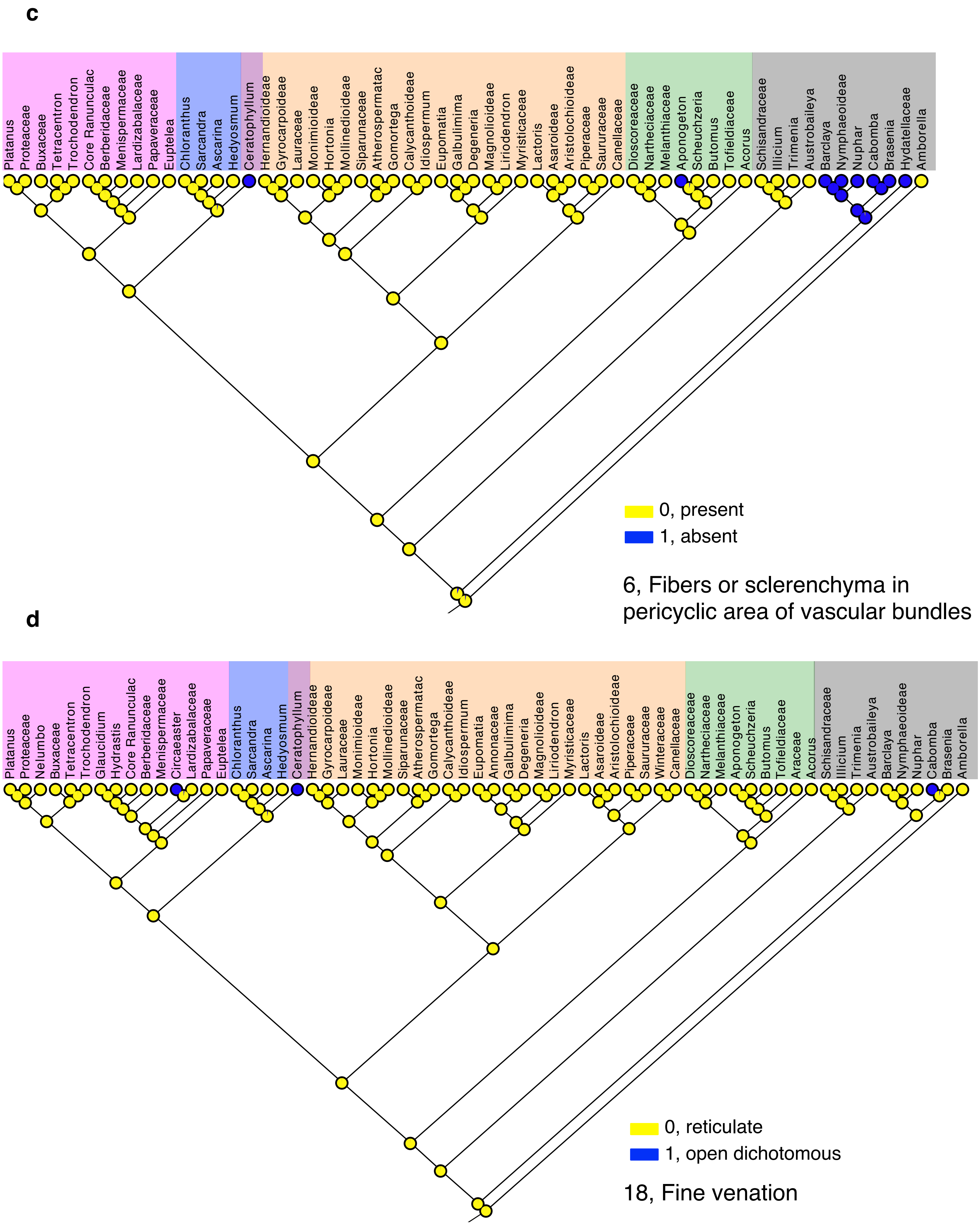

Supplementary Fig. 19 (c-d). Nine new morphological characters originated before the diversification of Ceratophyllaceae. Eudicots, Chloranthaceae, Ceratophyllaceae, magnoliids, monocots and basal angiosperms are highlighted by pink, blue, dark purple, orange, green and gray, respectively.

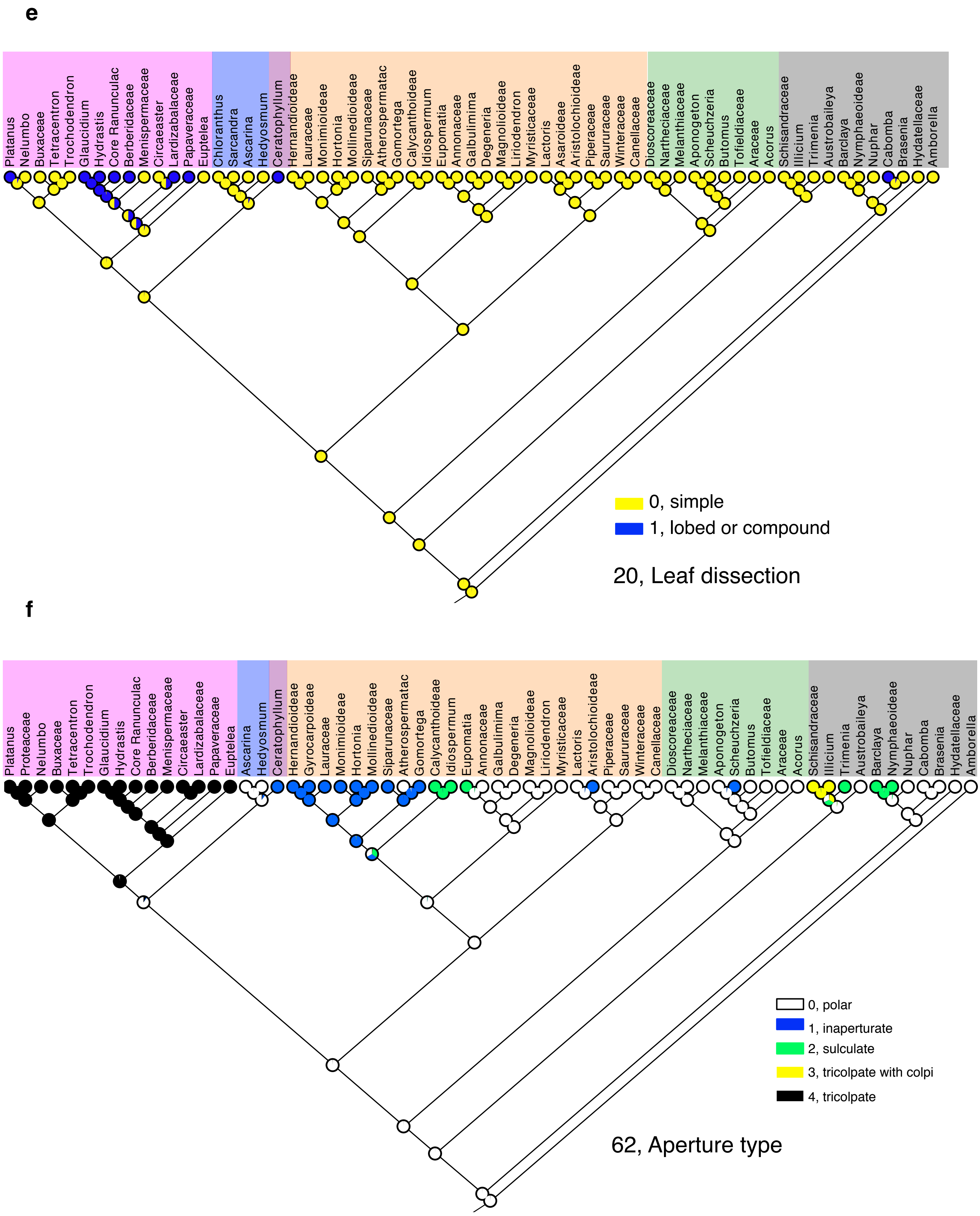

Supplementary Fig. 19 (e-f). Nine new morphological characters originated before the diversification of Ceratophyllaceae. Eudicots, Chloranthaceae, Ceratophyllaceae, magnoliids, monocots and basal angiosperms are highlighted by pink, blue, dark purple, orange, green and gray, respectively.

g

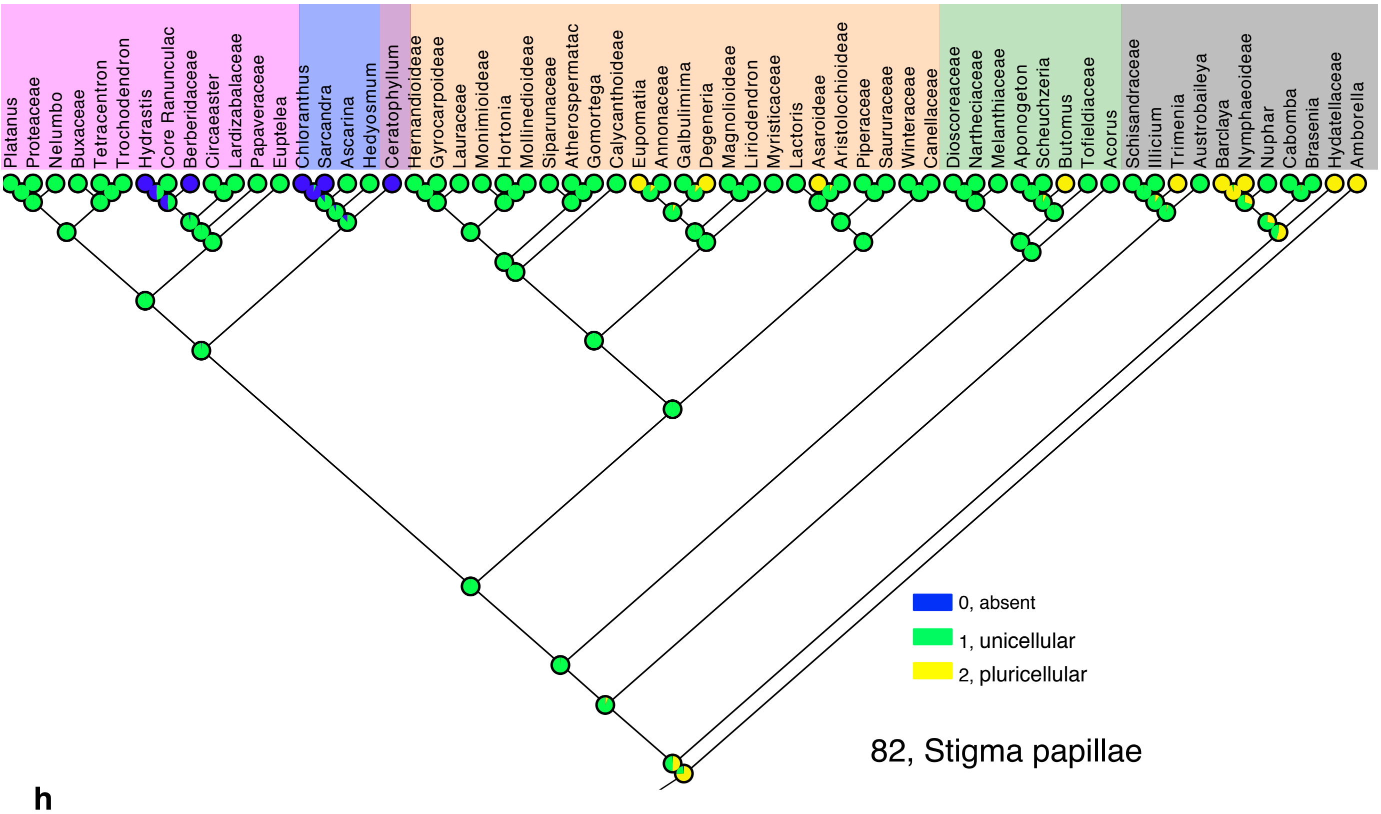

h

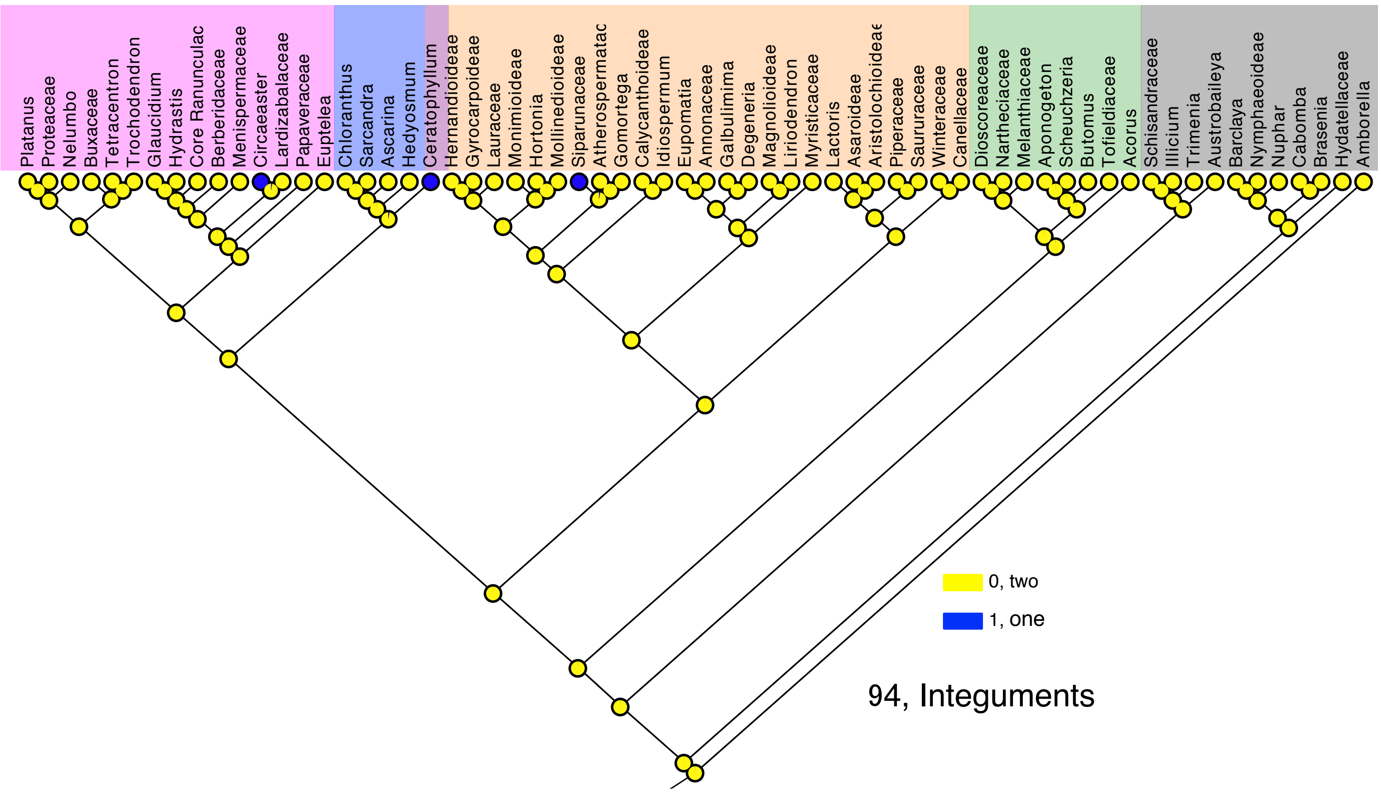

Supplementary Fig. 19 (g-h). Nine new morphological characters originated before the diversification of Ceratophyllaceae. Eudicots, Chloranthaceae, Ceratophyllaceae, magnoliids, monocots and basal angiosperms are highlighted by pink, blue, dark purple, orange, green and gray, respectively.

i

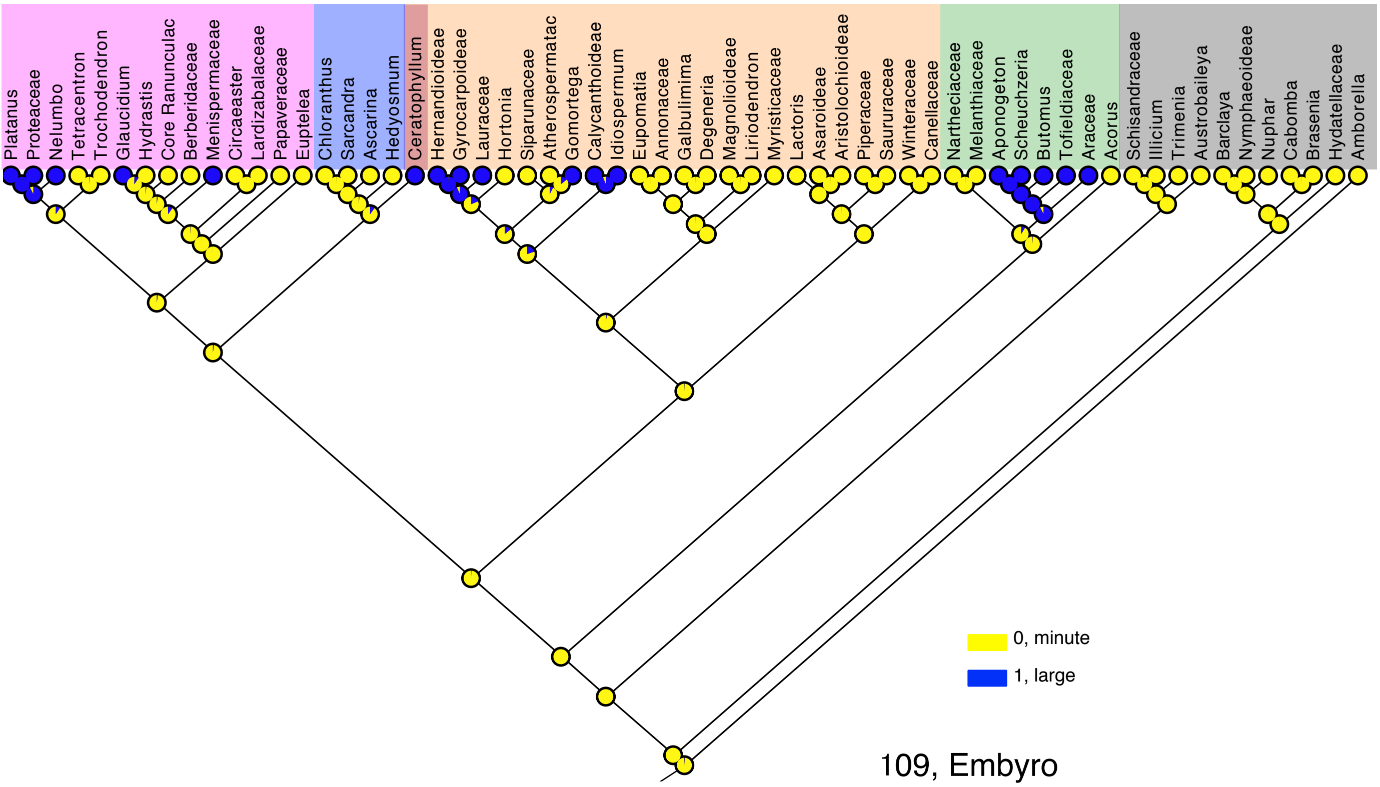

Supplementary Fig. 19 (i). Nine new morphological characters originated before the diversification of Ceratophyllaceae. Eudicots, Chloranthaceae, Ceratophyllaceae, magnoliids, monocots and basal angiosperms are highlighted by pink, blue, dark purple, orange, green and gray, respectively.

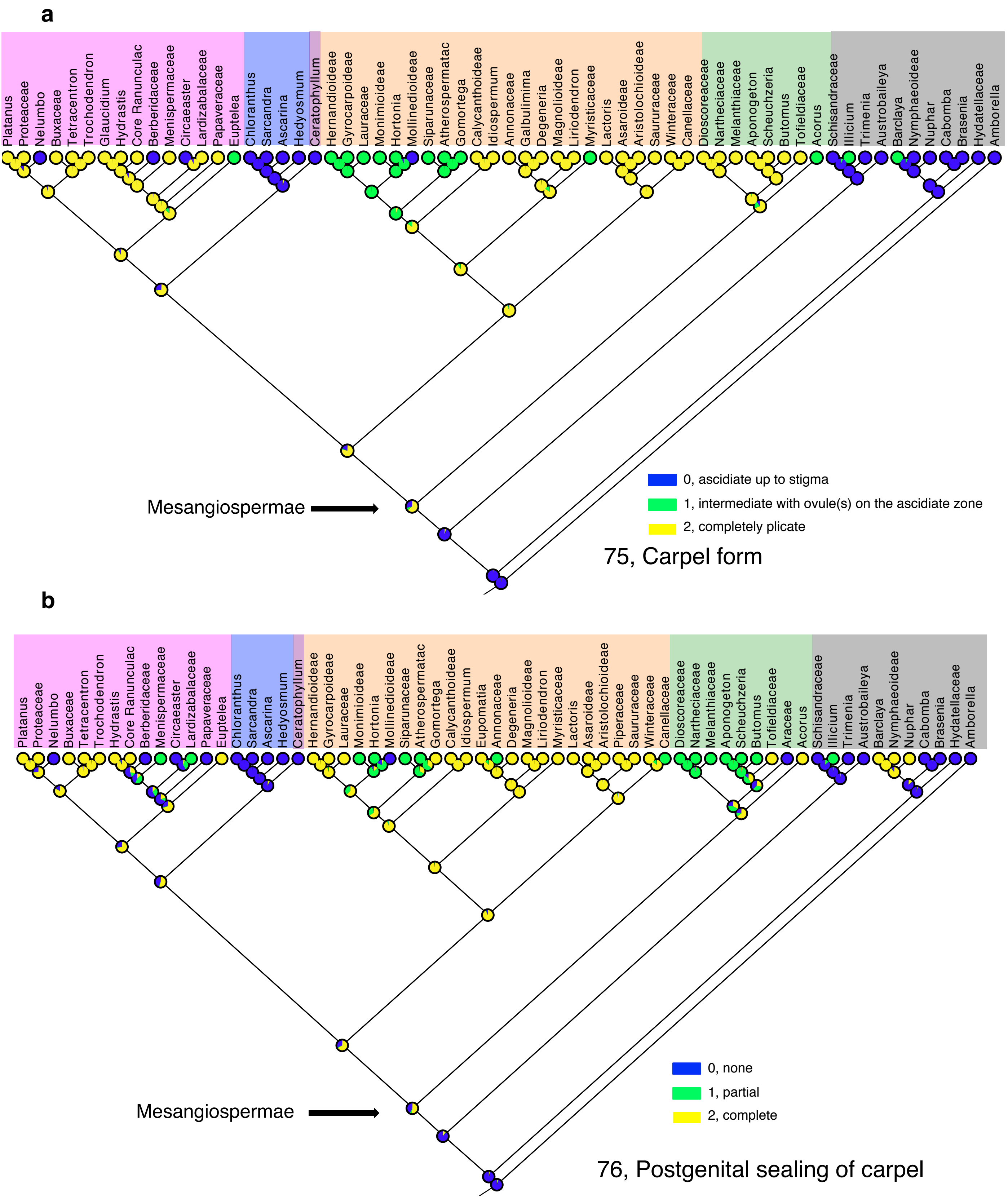

Supplementary Fig. 20. Evolutionary patterns of carpel form (a) and postgenital sealing of carpel (b) in the context of our topology. Eudicots, Chloranthaceae, Ceratophyllaceae, magnoliids, monocots and basal angiosperms are highlighted by pink, blue, dark purple, orange, green and gray, respectively.

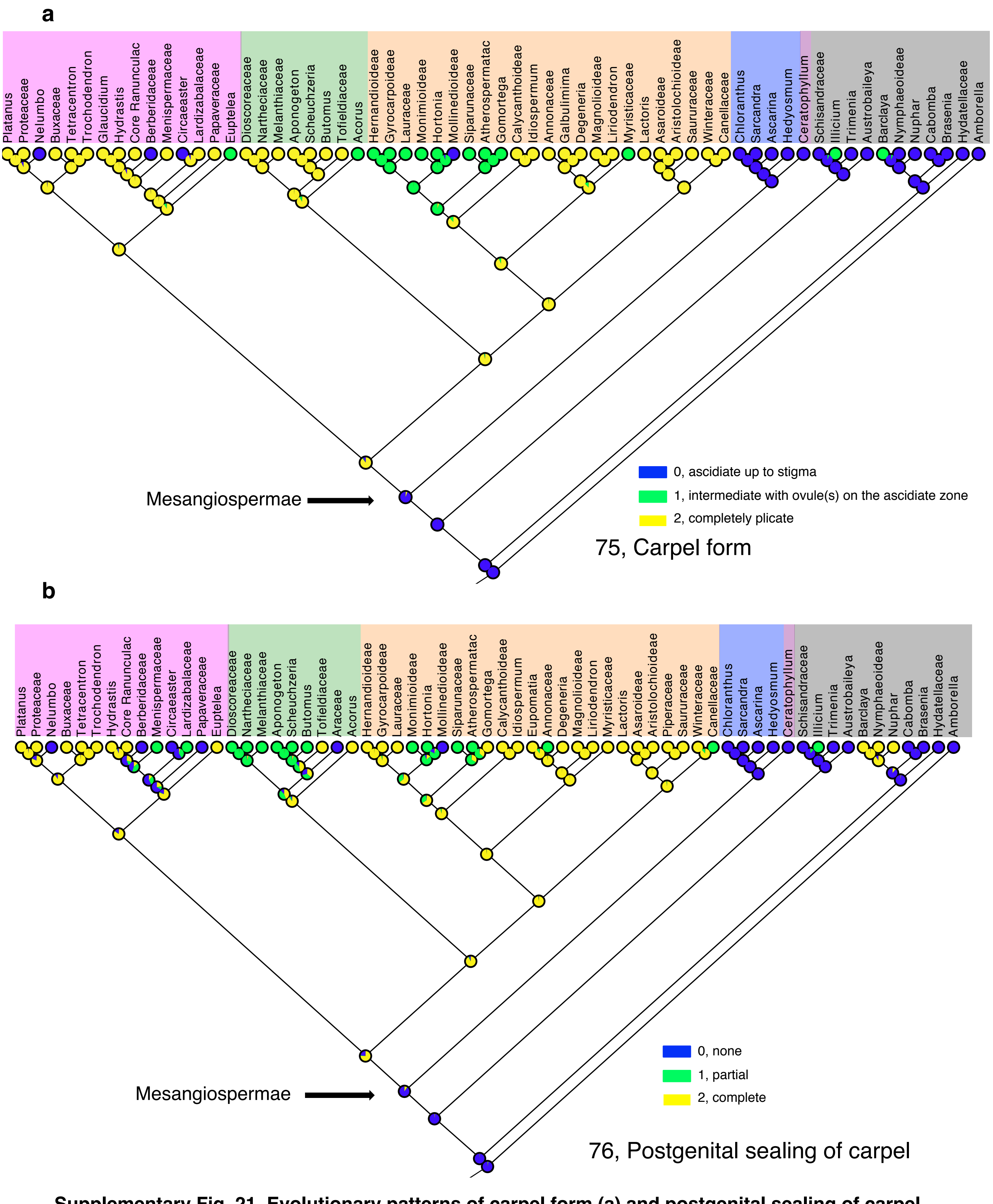

Supplementary Fig. 21. Evolutionary patterns of carpel form (a) and postgenital sealing of carpel (b) in the context of E&D tree as shown in Fig. 1d. Eudicots, Chloranthaceae, Ceratophyllaceae, magnoliids, monocots and basal angiosperms are highlighted by pink, blue, dark purple, orange, green and gray, respectively.

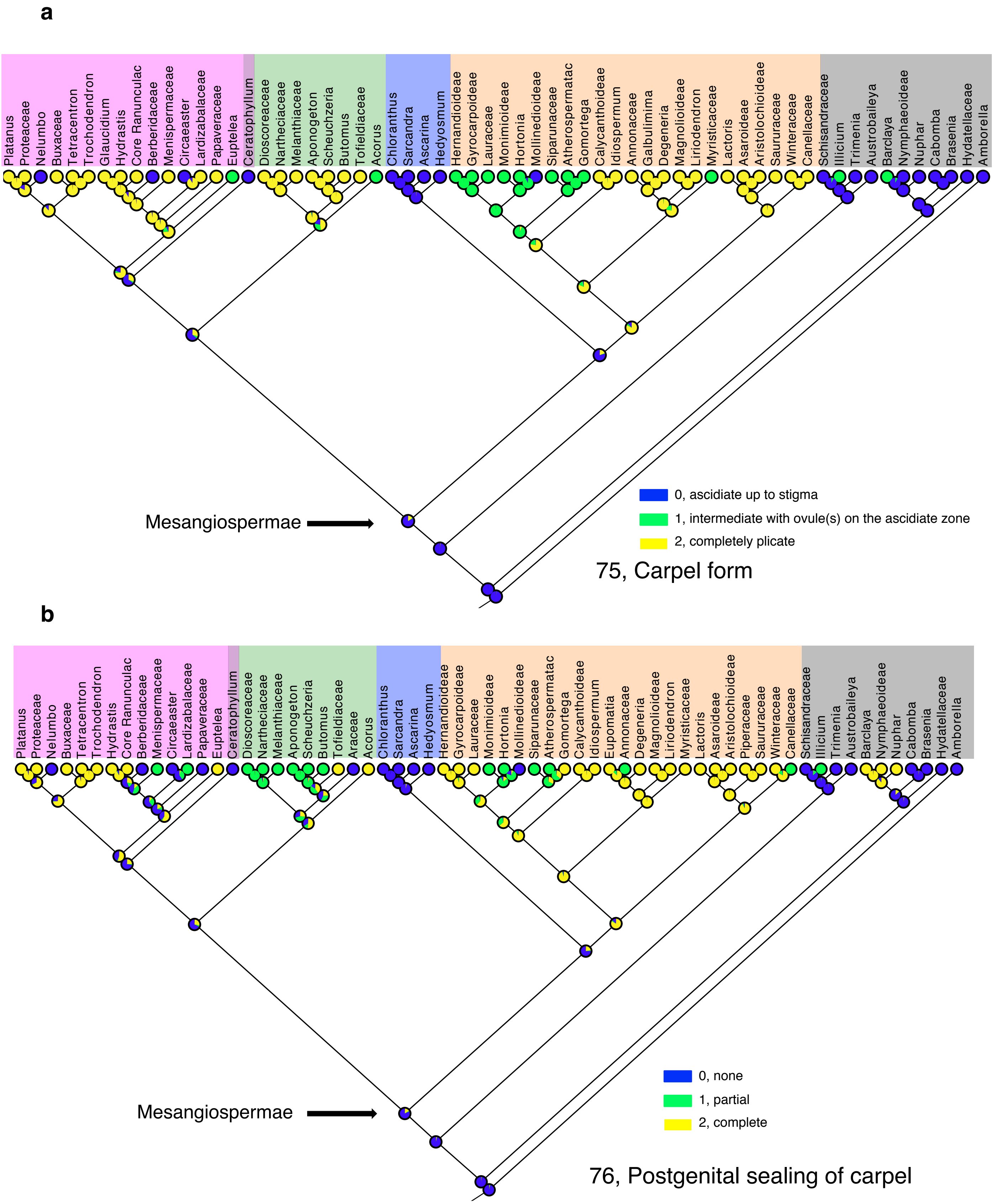

Supplementary Fig. 22. Evolutionary patterns of carpel form (a) and postgenital sealing of carpel (b) in the context of the topology accepted by APG III as shown in Fig. 1a. Eudicots, Chloranthaceae, Ceratophyllaceae, magnoliids, monocots and basal angiosperms are highlighted by pink, blue, dark purple, orange, green and gray, respectively.

Supplementary Table 1: Taxa included in this study

Species	Family	Order	Major lineage	Sources
Selaginella moellendorffii	Selaginellaceae	Selaginellales	Lycophyte	Phytozome
Pinus taeda	Pinaceae	Pinales	Gymnosperms	NCBI
Picea sitchensis	Pinaceae	Pinales	Gymnosperms	NCBI
Picea glauca	Pinaceae	Pinales	Gymnosperms	NCBI
Ginkgo biloba	Ginkgoaceae	Ginkgoales	Gymnosperms	This study
Amborella trichopoda	Amborellaceae	Amborellales	Basal angiosperm	Ancestral Angiosperm Genome Project (AAGP)
Nuphar advena	Nymphaeaceae	Nymphaeales	Basal angiosperm	Floral Genome Group (FGP)
Cabomba caroliniana	Cabombaceae	Nymphaeales	Basal angiosperm	This study
Illicium henryi	Schisandraceae	Austrobaileyales	Basal angiosperm	This study
Acorus calamus	Acoraceae	Acorales	Monocots	This study
Alisma plantago-aquatica	Alismataceae	Alismatales	Monocots	This study
Pinellia ternata	Araceae	Alismatales	Monocots	This study
Dioscorea opposita	Dioscoreaceae	Dioscoreales	Monocots	This study
Pandanus utilis	Pandanaceae	Pandanales	Monocots	This study
Lilium brownii	Lilaceae	Lilales	Monocots	This study
Asparagus officinalis	Asparagaceae	Asparagales	Monocots	This study
Irish japonica	Iridaceae	Asparagales	Monocots	This study
Yucca filamentosa	Agavaceae	Asparagales	Monocots	This study
Trachycarpus fortunei	Arecaceae	Arecales	Monocots	This study
Canna indica	Cannaceae	Zingiberales	Monocots	This study
Musa acuminata	Musaceae	Zingiberales	Monocots	Phytozome
Sorghum bicolor	Poaceae	Poales	Monocots	Phytozome
Setaria italica	Poaceae	Poales	Monocots	Phytozome

Oryza sativa	Poaceae	Poales	Monocots	Phytozome
Brachypodium distachyon	Poaceae	Poales	Monocots	Phytozome
Zea mays	Poaceae	Poales	Monocots	Phytozome
Panicum vitgatum	Poaceae	Poales	Monocots	Phytozome
Houttuynia cordata	Saururaceae	Piperales	Magnoliids	This study
Aristolochia tagala	Aristolochiaceae	Piperales	Magnoliids	This study
Saruma henryi	Aristolochiaceae	Piperales	Magnoliids	FGP
Liriodendron tulipifera	Magnoliaceae	Magnoliales	Magnoliids	This study
Magnolia denudata	Magnoliaceae	Magnoliales	Magnoliids	This study
Chimonanthus praecox	Calycanthaceae	Laurales	Magnoliids	This study
Cinnamomum camphora	Lauraceae	Laurales	Magnoliids	This study
Persea americana	Lauraceae	Laurales	Magnoliids	AAGP
Ceratophyllum demersum	Ceratophyllaceae	Ceratophyllales	Ceratophyllales	This study
Ceratophyllum oryzetorum	Ceratophyllaceae	Ceratophyllales	Ceratophyllales	This study
Chloranthus japonicus	Chloranthaceae	Chloranthales	Chloranthales	This study
Sarcandra glabra	Chloranthaceae	Chloranthales	Chloranthales	This study
Buxus sinica	Buxaceae	Buxales	Basal eudicots	This study
Platanus x acerifolia	Platanaceae	Proteales	Basal eudicots	This study
Nelumbo necifera	Nelumbonaceae	Proteales	Basal eudicots	NCBI
Meliosma arviflora	Sabiaceae	Sabiaceae	Basal eudicots	This study
Eschscholzia californica	Papaveraceae	Ranunculales	Basal eudicots	NCBI
Aquilegia coerulea	Ranunculaceae	Ranunculales	Basal eudicots	Phytozome
Vitis vinifera	Vitaceae	Vitales	Core eudicots	Phytozome
Solanum lycopersicum	Solanaceae	Solanales	Asterids	Phytozome
Solanum tuberosum	Solanaceae	Solanales	Asterids	Phytozome

Mimulus guttatus	Scrophulariaceae	Lamiales	Asterids	Phytozome
Lactuca sativa	Asteraceae	Asterales	Asterids	This study
Artemisia annua	Asteraceae	Asterales	Asterids	NCBI
Eucalyptus grandis	Myrtaceae	Myrtales	Rosids	Phytozome
Prunus persica	Rosaceae	Rosales	Rosids	Phytozome
Fragaria vesca	Rosaceae	Rosales	Rosids	Phytozome
Glycine max	Fabaceae	Fabales	Rosids	Phytozome
Medicago truncatula	Fabaceae	Fabales	Rosids	Phytozome
Phaseolus vulgaris	Fabaceae	Fabales	Rosids	Phytozome
Citrus clementina	Rutaceae	Sapindales	Rosids	Phytozome
Citrus sinensis	Rutaceae	Sapindales	Rosids	Phytozome
Theobroma cacao	Sterculiaceae	Malvales	Rosids	Phytozome
Gossypium raimondii	Malvaceae	Malvales	Rosids	Phytozome
Carica papaya	Caricaceae	Brassiacales	Rosids	Phytozome
Arabidopsis thaliana	Brassicaceae	Brassiacales	Rosids	Phytozome
Arabidopsis lyrata	Brassicaceae	Brassiacales	Rosids	Phytozome
Thellungiella parvula	Brassicaceae	Brassiacales	Rosids	Phytozome
Capsella rubella	Brassicaceae	Brassiacales	Rosids	Phytozome
Populus trichocarpa	Salicaceae	Malpighiales	Rosids	Phytozome
Ricinus communis	Euphorbiaceae	Malpighiales	Rosids	Phytozome
Manihot esculenta	Euphorbiaceae	Malpighiales	Rosids	Phytozome

Supplementary Table 2: Information of 59 orthologous groups.

ID of OG	Length of alignment (after trimal, AA)	Number of species	Ratio of missing data (%)	Parsimony informative sites (%)	Gene ID (*A. thaliana*)	Length of *A. thaliana* genes (AA)	Functional annotation
**432206**	389	55	7.95	57.58	**AT1G05055**	421	Transcription factor TFIIH complex, involved in transcription and DNA repair and interacted with AtXPD.
**432635**	289	55	3.14	50.87	**AT1G10830**	367	15-Cis-Zeta-Carotene Isomerase (Z-ISO).
**432730**	298	47	5.91	57.05	**AT1G14140**	305	Uncoupling Protein 3 (*UCP3*), mitochondrial substrate carrier family protein.
**433020**	410	52	5.45	65.85	**AT1G16280**	491	DEAD-box RNA helicase, RNA Helicase 36 (*RH36*).
**432715**	294	54	4.99	57.82	**AT1G48270**	326	G-Protein-Coupled Receptor 1 (*GCR1*).
**432442**	313	54	8.27	72.84	**AT1G52530**	319	*Hus1*-like protein.
**432589**	317	52	3.27	69.72	**AT1G55760**	329	BTB/POZ domain-containing protein.
**431769**	229	53	3.09	66.38	**AT1G65230**	286	Function unknown.
**432437**	616	52	5.42	44.81	**AT1G79600**	711	Protein kinase superfamily protein, protein phosphorylation.
**MCM5**	741	57	23.61	49.12	**AT2G07690**	727	Minichromosome Maintenance 5 (*MCM5*).
**432859**	761	54	12.58	63.86	**AT2G13540**	848	ABA Hypersensitive 1 (*ABH1*), involved in ABA signaling and flowering.
**431559**	316	54	4.34	57.91	**AT2G17265**	370	Homoserine Kinase (*HSK*).
**433581**	306	53	9.75	72.88	**AT2G18410**	374	Function unknown.
**434655**	454	58	8.51	44.71	**AT2G20330**	648	Transducin/WD40 repeat-like superfamily protein.
**431509**	175	56	3.55	40.00	**AT2G26430**	416	Arginine-rich Cyclin 1 (*RCY1*), encodes an ania-6a type arginine-rich cyclin.
**431208**	318	54	11.16	70.75	**AT2G26590**	300	Regulatory Particle Non-Atpase 13 (*RPN13*), involved in cell adhesion.
**432026**	296	54	4.70	64.86	**AT2G31040**	350	ATP synthase protein I-related.
**432130**	197	54	0.60	65.99	**AT2G34690**	206	Accelerated Cell Death 11 (*ACD11*).
**432298**	249	55	7.73	66.27	**AT2G35320**	307	Eyes Absent Homolog (*EYA*), encodes a tyrosine-specific phosphatase.
**433773**	276	43	1.81	73.91	**AT2G38760**	321	Annexin 3 (*ANNAT3*).
**432462**	296	53	2.09	57.43	**AT3G10970**	365	Haloacid dehalogenase-like hydrolase (HAD) superfamily protein, involved in metabolic process.

**431639**	469	54	11.33	79.32	**AT3G15180**	519	ARM repeat superfamily protein.
**434509**	227	52	7.02	63.88	**AT3G17590**	240	Bushy Growth (*BSH*), represents a conserved subunit of plant SWI/SNF complexes.
**432200**	330	52	4.05	53.94	**AT3G20020**	435	Protein Arginine Methyltransferase 6 (*PRMT6*), involved in protein amino acid methylation.
**434734**	325	55	4.93	62.46	**AT3G22590**	415	Plant Homologous to Parafibromin (*PHP*).
**MSH1**	1036	52	29.55	57.05	**AT3G24320**	1118	Mutl Protein Homolog 1 (*MSH1*), promotes re-arrangements of mitochondrial genome.
**431723**	257	52	9.24	60.31	**AT3G52210**	355	S-adenosyl-L-methionine-dependent methyltransferases superfamily protein, involved in mRNA capping.
**434468**	420	55	4.50	63.81	**AT3G53110**	496	Encodes a putative DEAD-Box RNA Helicase and has RNA-dependent ATPase activity, low Expression of Osmotically Responsive Genes 4 (*LOS4*)
**SMC1**	1172	56	16.44	63.91	**AT3G54670**	1239	Titan8 (*TTN8*), *SMC1*, encodes a member of the Arabidopsis cohesin complex that is essential for viability and sister chromatid alignment.
**435069**	470	53	5.49	58.30	**AT3G59040**	590	Tetratricopeptide repeat (TPR)-like superfamily protein, function unknown.
**432683**	1044	55	13.49	68.30	**AT3G62360**	1227	Carbohydrate-binding-like fold; functions in carbohydrate binding
**435038**	504	52	2.73	57.54	**AT4G00740**	600	Quasimodo 3 (*QUA3*), encodes a Golgi-localized type II membrane pectin methyltransferase regulating cell wall biosynthesis in suspension cells.
**MLH1**	722	55	27.15	55.54	**AT4G09140**	737	Mutl-homologue 1 (*MLH1*), encodes a protein with similarity to Mut1 DNA mismatch repair protein, from *E.coli*.
**433443**	650	53	9.09	59.69	**AT4G11160**	743	Translation initiation factor 2, small GTP-binding protein, functions in GTP binding, GTPase activity, translation initiation factor activity; involved in translational initiation.
**434643**	161	49	5.50	72.67	**AT4G18930**	181	RNA ligase/cyclic nucleotide phosphodiesterase family protein.
**432280**	406	55	4.98	62.32	**AT4G23820**	444	Pectin lyase-like superfamily protein; functions in polygalacturonase activity; involved in carbohydrate metabolic process.
**431496**	169	54	2.13	60.95	**AT4G29520**	306	Function unknown.
**432473**	422	48	7.99	70.14	**AT4G35880**	524	Eukaryotic aspartyl protease family protein, involved in proteolysis.
**435119**	344	52	10.49	70.93	**AT4G36440**	394	Function unknown.
**432713**	525	57	10.96	53.14	**AT4G38890**	700	FMN-linked oxidoreductases superfamily protein; functions in tRNA dihydrouridine synthase activity, FAD binding, catalytic activity, zinc ion binding, nucleic acid binding; involved in oxidation reduction, tRNA processing, metabolic process.
**432869**	447	54	9.52	62.19	**AT5G03900**	523	Iron-sulphur cluster biosynthesis family protein.
**431993**	355	56	7.54	56.62	**AT5G08170**	383	Embryo Defective 1873 (*EMB1873*).
**432875**	232	55	7.77	79.31	**AT5G11810**	306	Function unknown.
**434840**	302	56	5.56	46.69	**AT5G12040**	369	Nitrilase/cyanide hydratase and apolipoprotein N-acyltransferase family protein; functions in hydrolase activity, acting on carbon-nitrogen (but not peptide) bonds, zinc ion binding;

							involved in nitrogen compound metabolic process.
**432842**	564	53	5.16	46.81	**AT5G13400**	624	Major facilitator superfamily protein; functions in transporter activity; involved in oligopeptide transport.
**434860**	330	53	3.31	45.45	**AT5G17230**	437	Encodes phytoene synthase (*PSY*).
**432801**	525	55	11.00	75.81	**AT5G20170**	653	RNA polymerase II transcription mediators; functions in RNA polymerase II transcription mediator activity; involved in regulation of transcription from RNA polymerase II promoter.
**433634**	545	57	8.19	65.14	**AT5G42480**	801	Accumulation and replication of chloroplasts 6 (*ARC6*). Probably involved in functions in the assembly and/or stabilization of the plastid-dividing FtsZ ring, inhibiting FtsZ filament formation in the chloroplast.
**433902**	315	56	12.53	52.06	**AT5G51140**	395	Pseudouridine synthase family protein; functions in pseudouridine synthase activity; involved in pseudouridine synthesis, RNA modification.
**431271**	841	56	8.64	61.24	**AT5G51430**	836	Embryo Yellow (*EYE*). It is likely to be involved in transport or retention of Golgi-localized proteins and in maintenance of Golgi morphology.
**434965**	629	51	7.52	60.73	**AT5G52580**	673	RabGAP/TBC domain-containing protein; functions in RAB GTPase activator activity; involved in regulation of Rab GTPase activity.
**431690**	121	53	0.81	52.07	**AT5G54855**	146	Pollen Ole e 1 allergen and extensin family protein; function unknown.
**434304**	345	54	9.22	59.13	**AT5G62030**	453	Diphthamide synthesis DPH2 family protein, function unknown.
**SMC2**	1158	56	23.21	55.79	**AT5G62410**	1175	Structural Maintenance of Chromosomes 2 (*SMC2*).
**434354**	519	53	8.90	71.48	**AT5G64440**	607	Fatty Acid Amide Hydrolase (*FAAH*).
**432653**	313	54	7.07	70.93	**AT5G64510**	424	Tunicamycin Induced 1 (*TIN1*), a plant-specific ER stress-inducible protein.
**431774**	308	54	3.38	53.25	**AT5G65860**	346	Ankyrin repeat family protein; functions in methyltransferase activity; involved in metabolic process.
**431562**	333	54	9.97	58.86	**AT5G66180**	395	S-adenosyl-L-methionine-dependent methyltransferases superfamily protein.
**433564**	214	56	1.88	54.67	**AT5G66530**	307	Galactose mutarotase-like superfamily protein; functions in carbohydrate binding, isomerase activity, aldose 1-epimerase activity, catalytic activity; involved in galactose metabolic process, carbohydrate metabolic process.

Supplementary Table 3: Information of genes from the plastid inverted repeat regions.

Gene	Length (bp)	PI_sites (bp)	PI_percentage (%)
trnI.GAU	72	2	2.8
trnR.AGC	74	3	4.1
trnA.UGC	73	3	4.1
trnL.CAA	81	5	6.2
trnV.GAC	72	5	6.9
rrn16	1491	167	11.2
rrn23	2787	316	11.3
trnI.CAU	74	9	12.2
trnN.GUU	72	9	12.5
rrn5	121	17	14.0
rrn4.5	103	18	17.5
rps12	255	47	18.4
ndhB	1530	307	20.1
trnA.UGC.intron	761	162	21.3
rps12.intron	519	113	21.8
ndhB.intron	694	153	22.0
trnI.GAU.intron	892	215	24.1
rpl2.intron	608	159	26.2
rps7	465	145	31.2
rrn4.5.rrn5.spacer	222	74	33.3
rpl2.rpl23.spacer	18	6	33.3
ycf2.trnL.spacer	917	326	35.6
rpl2	822	315	38.3
trnA.rrn23.spacer	143	55	38.5
ndhB.rps7.spacer	304	117	38.5
trnV.rrn16.spacer	197	78	39.6
rps7.rps12.spacer	49	20	40.8
trnL.ndhB.spacer	550	234	42.5
rrn23.rrn4.5.spacer	95	41	43.2
rpl23.trnI.spacer	158	70	44.3
rrn16.trnI.spacer	240	107	44.6
rps12.trnV.spacer	1647	736	44.7
rpl23	264	120	45.5
trnI.trnA.spacer	63	29	46.0
trnN.ycf1.spacer	271	128	47.2
trnR.trnN.spacer	444	212	47.7
rrn5.trnR.spacer	177	85	48.0
ycf2	6606	3531	53.5
trnI.ycf2.spacer	60	37	61.7
ycf1	711	457	64.3
Total	24702	8633	34.9

Supplementary Table 4: Information of the 83 plastid genes. Genes from the inverted repeat regions are highlighted in bold.

Gene	Length (bp)	PI_sites (bp)	PI_percentage (%)
rrn16	1491	167	11.2
rrn23	2786	319	11.5
rrn5	121	17	14.0
rrn4.5	102	17	16.7
ndhB	1530	302	19.7
rps12	369	94	25.5
psbD	1059	288	27.2
petN	87	25	28.7
psbL	114	33	28.9
psbA	1059	307	29.0
psbF	117	34	29.1
petB	645	195	30.2
atpH	243	75	30.9
rps7	465	146	31.4
psbC	1369	434	31.7
petD	480	153	31.9
psbE	249	80	32.1
psaB	2202	709	32.2
psaA	2250	732	32.5
psbN	129	43	33.3
psbB	1524	523	34.3
ycf3	501	176	35.1
psbJ	120	43	35.8
psaC	243	88	36.2
rbcL	1425	526	36.9
petG	111	41	36.9
rpl2	816	310	38.0
atpI	741	288	38.9
psbI	108	42	38.9
atpB	1494	588	39.4
psbT	99	39	39.4
psbM	96	38	39.6
psbZ	186	74	39.8
ndhC	360	144	40.0
rpl23	264	110	41.7
ndhH	1179	500	42.4
atpA	1518	648	42.7
ndhI	474	211	44.5
ndhE	303	139	45.9
ndhK	675	314	46.5
petA	960	447	46.6
psbH	219	102	46.6
ndhJ	474	222	46.8
psaJ	132	62	47.0
rps16	231	109	47.2

rpl16	402	199	49.5
rpl14	366	185	50.5
ndhA	1089	552	50.7
rpoB	3213	1654	51.5
rpl36	111	58	52.3
ycf2	6768	3587	53.0
rpoC1	1980	1050	53.0
ndhD	1488	793	53.3
psaI	108	58	53.7
rps4	591	318	53.8
psbK	183	101	55.2
ndhG	528	292	55.3
rps14	300	166	55.3
rps18	285	159	55.8
infA	231	129	55.8
rps2	708	399	56.4
atpE	398	228	57.3
petL	93	54	58.1
ycf4	546	318	58.2
rps19	276	163	59.1
atpF	552	330	59.8
ndhF	1956	1176	60.1
rps11	414	251	60.6
rps8	399	243	60.9
rpl33	192	119	62.0
ccsA	807	502	62.2
rpoA	999	624	62.5
accD	1452	914	62.9
rpoC2	4104	2618	63.8
ycf1	714	458	64.1
cemA	675	444	65.8
rps3	639	421	65.9
rpl20	342	228	66.7
clpP	585	392	67.0
rpl32	141	95	67.4
rps15	249	168	67.5
rpl22	321	222	69.2
matK	1416	1094	77.3
Total	66741	30716	46

Supplementary Table 5: Information of trees reconstructed by using IR genes with different evolutionary rates. The dash indicates incongruent topology.

matrix	gene number	length (bp)	PI (%)	PI_sites (bp)	CC (bootstrap)	CCE (bootstrap)	Mesodicots (bootstrap)	Mesangiospermae (bootstrap)
IR_less_30	18	10,279	16.6	1,710	25%	26%	34%	100%
IR_greater_30	22	14,423	47.9	6,923	88%	58%	62%	100%
IR_less_40	26	13,367	21.1	2,826	63%	65%	--	100%
IR_greater_40	14	11,335	51.2	5,807	70%	53%	64%	96%
IR_less_50	37	17,325	26.6	4,608	70%	49%	55%	100%
IR_greater_50	3	7,377	54.6	4,025	71%	73%	46%	99%
IR_all_genes	40	24,702	34.9	8,633	86%	83%	80%	100%

CC: (Ceratophyllales, Chloranthales);

CCE: ((Ceratophyllales, Chloranthales), Eudicots);

Supplementary Table 6: Divergence times of major nodes estimated by r8s and BEAST using 1st codon position, 1st + 2nd codon positions, and all codon positions, respectively, with the eudicot calibration (125 Myr) being treated as fixed or the minimum. Ranges correspond to 95% highest posterior density (HPD).

	with eudicot calibration being the minimum (r8s)			with eudicot calibration being fixed (r8s)			with eudicot calibration being fixed (BEAST)		
	1st codon	1st + 2nd codons	All codons	1st codon	1st + 2nd codons	All codons	1st codon	1st + 2nd codons	All codons
Angiospermae	239 (227-252)	237 (227-249)	240 (233-248)	227 (216-241)	225 (216-240)	225 (216-232)	231 (191-280)	221 (193-261)	209 (186-246)
Nymphaeales + Austrobaileyales + Mesangiospermae	222 (214-230)	222 (215-229)	227 (222-233)	208 (200-216)	208 (202-214)	210 (205-214)	205 (173-266)	200 (177-240)	195 (175-218)
Austrobaileyales + Mesangiospermae	209 (203-218)	208 (201-214)	211 (205-217)	193 (187-200)	192 (187-197)	190 (187-194)	186 (158-250)	181 (156-216)	180 (160-195)
Mesangiospermae	189 (182-197)	187 (181-195)	191 (185-196)	171 (166-176)	170 (166-175)	167 (164-170)	161 (141-216)	154 (142-176)	170 (150-171)
Mesodicots	185 (178-192)	182 (177-189)	187 (182-192)	165 (162-170)	163 (160-168)	162 (159-165)	152 (128-180)	148 (138-167)	155 (143-167)
Chloranthaceae + Ceratophyllaceae + eudicots	179 (173-186)	177 (171-184)	182 (177-187)	158 (155-163)	157 (154-161)	157 (154-159)	144 (132-165)	141 (133-156)	149 (140-160)
Chloranthaceae + Ceratophyllaceae	165 (158-173)	165 (158-172)	170 (164-174)	146 (142-151)	146 (142-151)	146 (143-149)	126 (71-151)	126 (104-142)	133 (106-147)
magnoliids	172 (165-179)	169 (161-174)	175 (169-180)	154 (149-161)	151 (147-155)	152 (149-155)	131 (34-163)	136 (115-151)	136 (122-150)
monocots	166 (160-173)	166 (160-171)	169 (165-174)	151 (145-156)	151 (145-156)	149 (147-152)	143 (119-193)	134 (120-158)	138 (127-149)
eudicots	151 (146-157)	149 (146-155)	156 (151-161)	125	125	125	125	125 (125-128)	126 (115-126)
